# Asymmetric α-spirocyclopropanation of oxindoles and benzofuranones via dynamic kinetic resolution

**DOI:** 10.1038/s42004-022-00695-3

**Published:** 2022-09-06

**Authors:** Yang Hu, Jie Yuan, Zheyao Li, Lin Zhao, Jianhong Zhao, Xinhong Yu

**Affiliations:** 1 Engineering Research Center of Pharmaceutical Process Chemistry, Ministry of Education, Shanghai Key Laboratory of New Drug Design, School of Pharmacy, East China University of Science & Technology, Shanghai, People’s Republic of China; 2 State Key Laboratory of Bioengineering Reactors, East China University of Science & Technology, Shanghai, 200237 People’s Republic of China

**Keywords:** Asymmetric synthesis, Synthetic chemistry methodology

## Abstract

Chiral benzo five-membered heterocyclic spirocyclopropanes are an important class of parent core structures with pharmacological activity. A novel organocatalytic one-pot cascade ether oxidation iminium-ion activation strategy for the asymmetric spirocyclopropylation of benzofuran-2-ones and indolin-2-ones from allyl tert-butyl ethers/ pent-2,4-dienyl ethyl ethers with excellent enantioselectivity (ee% up to > 99) and diastereoselectivity(dr.% up to 91:9) has been developed. This process involves the successful dynamic kinetic resolution of racemic 3-bromobenzofuran-2-ones or 3-bromoindolin-2-ones. Its synthetic application will provide a new aminocatalytic cascade tool for the efficient synthesis of complex molecules.

## Abstract (web summary)

Chiral benzo five-membered heterocyclic spirocyclopropanes are pharmacologically active scaffolds, but access to spiral rings with multiple stereocenters is challenging. Here, the authors report an organocatalytic one-pot cascade ether oxidation iminium-ion activation strategy for the asymmetric spirocyclopropylation of benzofuran-2-ones and indolin-2-ones with excellent enantio- and diastereoselectivity.

## Introduction

Organocatalytic enal-derived iminium-enamine activated asymmetric transformations have been demonstrated as powerful approaches for constructing enantiomerically enriched and functionalized aldehydes^1–11^. Oxidative cascade strategies, in which the iminium cation is generated in situ, are an attractive alternative to previous investigations in iminium catalysis and have been what developed because of its convenient one-pot-access, decreasing time and cost^12,13^.

In this context, oxidative cascade procedures involving the release of unstable enals in situ have been developed. In 2011, Rueping and co-workers reported enantioselective oxidative iminium activation for the functionalization of aldehydes from allylic alcohols^14^. Hayashi and Wang have independently described a new transformation, oxidative enamine catalysis for the direct asymmetric β-functionalization of simple aldehydes, in which direct oxidation of an enamine occurs to generate an iminium species followed by subsequent reaction with a nucleophile^15,16^.

Ethers are ubiquitous in nature and synthetic chemistry, and importantly, the stability of the C-O bond of ethers renders it a starting material that is easy to store. In particular, the oxidative cleavage of ether bonds to generate chemically reactive aldehydes or ketones should be useful for organic synthesis because it occurs under neutral and mild conditions and remains a topic of interest^17–19^.

**Fig. 1:**
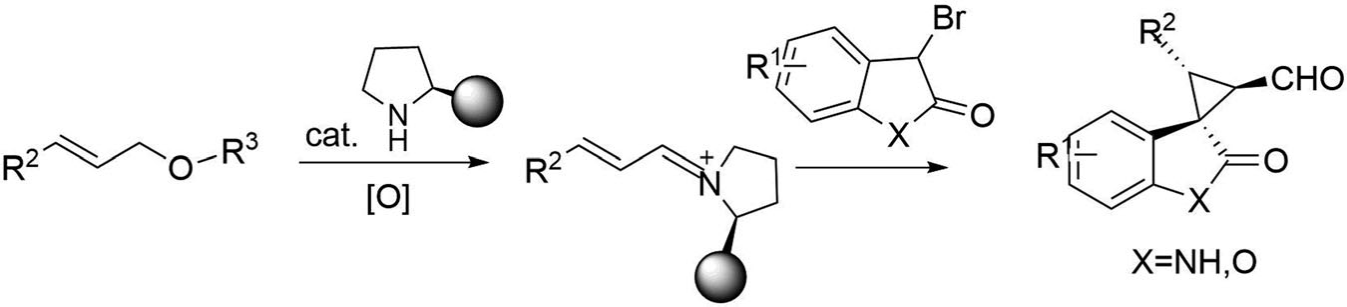
Oxidative iminium-ion activation. A strategic approach to amino catalysis using ethers as substrates envisaged in this paper.

To our knowledge, the development of the oxidative cleavage of cinnamyl alkyl ethers to access enals-derived iminium ions should be an attractive synthetic strategy in which ethers can be used as “masked” aldehydes for further iminium catalysis. Therefore, we wondered whether an amino-catalyzed strategy using ether as a substrate could be devised for the cationic activation of ether oxidation of unsaturated imines for oxidative cascade processes (Fig. 1).

**Fig. 2:**
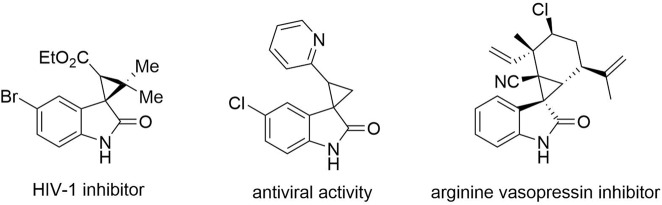
Bioactive spirocyclopropyloxindoles. Three spirocyclopropyl oxindole compounds that have been shown to be biologically active.

**Fig. 3:**
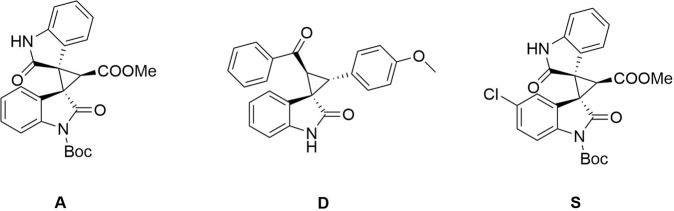
Related compounds with potential antitumor activity. Three compounds with potential biological activity that can be synthesized by the methods of this manuscript.

3-spirocyclopropyl -oxindole and -benzofuranone derivatives bearing a quaternary stereogenic center at the 3-position are known to exhibit remarkable biological and pharmaceutical activities^20–27^ and interest in synthetic methodologies for construction of these frameworks remains undiminished (Figs. 2 and 3)^23–27^.

Although some catalytic asymmetric methods can be used for the chemical synthesis of these scaffolds, the development of spiral rings with multiple stereocenters provided by ether-oxidation cascade strategies has not been realized. Motivated by the work of Wang and co-workers, who published a preliminary demonstration that organocatalytic asymmetric cascade reactions of enals with bromomalonates serve as a powerful approach to the preparation of highly enantio- and dia-stereoselective cyclopropanes^28^, we hypothesized that if racemic 3-bromobenzofuran-2-ones and 3-bromoindolin-2-ones^29–31^ with both electrophilicity and nucleophilicity could be successfully applied the interconversion between enantiomers properties in novel ether oxidation following asymmetric Michael addition initial cascade reaction, then stereoconvergent construction of 3-spirocyclopropyl-oxindoles and -benzofuranones could be accessed via the successful dynamic kinetic resolution of racemic 3-bromobenzo-five-membered-lactone/-lactam.

## Results and discussion

In order to explore the potential of the disclosed oxidative cascade methodology, we initiated our work by investigating the reaction of 3-bromooxindole (1a) and cinnamyl alkyl ether (2, *n* = 1) using 2,3-dichloro-5,6-dicyano-1, 4-benzoquinone (DDQ) as an oxidant in the presence of a secondary amine catalyst for promoting conversion of allyl ethers to α, β-spirocyclopropyl enals (Table 1, entries 1-16). Although DDQ has been shown to be an effective oxidant for the cleavage of ether bonds, a certain amount of water was necessary for the hydrolysis of the oxocarbenium-ion intermediate to complete the transformation to access aldehydes or ketones^32,33^

**Table 1:** Optimization of reaction conditions.


Entry	Cat.	R	n	Solvent	Oxidant	Base	Yield(%)^a^	ee(%)^b^	dr^c^	By-product(%)
1	**I**	Me	1	CHCl_3_	DDQ	NaOAc	31	98	88:12	
2	**I**	Et	1	CHCl_3_	DDQ	NaOAc	30	98	88:12	
3	**I**	Pr^i^	1	CHCl_3_	DDQ	NaOAc	39	99	90:10	
4	**I**	Bu^t^	1	CHCl_3_	DDQ	NaOAc	65	99	90:10	ND
5	**I**	Bu^t^	1	CHCl_3_	IBX	NaOAc	ND	ND	ND	ND
6	**I**	Bu^t^	1	CHCl_3_	MnO_2_	NaOAc	ND	ND	ND	ND
7	**I**	Bu^t^	1	CHCl_3_	TEMPO	NaOAc	ND	ND	ND	ND
8	**II**	Bu^t^	1	CHCl_3_	DDQ	NaOAc	62	99	89:19	ND
9	**III**	Bu^t^	1	CHCl_3_	DDQ	NaOAc	56	99	90:10	ND
10	**IV**	Bu^t^	1	CHCl_3_	DDQ	NaOAc	42	74	76:24	ND
11	**V**	Bu^t^	1	CHCl_3_	DDQ	NaOAc	42	−20	ND	ND
12	**VI**	Bu^t^	1	CHCl_3_	DDQ	NaOAc	30	33	ND	ND
13	**I**	Bu^t^	1	CHCl_3_	DDQ	NaHCO_3_	62	99	90:10	ND
14	**I**	Bu^t^	1	CHCl_3_	DDQ	Na_2_CO_3_	49	98	90:10	ND
15	**I**	Bu^t^	1	DMF	DDQ	NaOAc	ND	ND	ND	ND
16	**I**	Bu^t^	1	EtOH	DDQ	NaOAc	ND	ND	ND	ND
17	**I**	Me	2	CHCl_3_	DDQ	NaOAc	42	97	80:20	
18	**I**	Et	2	CHCl_3_	DDQ	NaOAc	55	98	85:15	ND
19	**I**	Pr^i^	2	CHCl_3_	DDQ	NaOAc	48	99	85:15	ND
20	**I**	Bu^t^	2	CHCl_3_	DDQ	NaOAc	55	98	80:20	ND

Unless stated otherwise, to a solution of **1a** (1.0 mmol) in the solvent was added of **2** (1.2 mmol), oxidant (1.2 mmol), base (2.0 mmol) and catalyst (0.2 mmol) and the reaction was stirred at RT for 24 h. ^a^ Yield of isolated product. ^b^ Determined by chiral HPLC analysis. ^c^ Determined by ^1^H NMR analysis of the crude products purified on a thin pad of silica gel.

To our delight, after the introduction of a tert-butyl ether, an excellent yield and stereoselectivity could be achieved in the presence of DDQ and TMS-protected diphenyl prolinol catalyst (**I**) (entry 4). Other exploited alkyl ethers such as methyl, ethyl, and iso-propyl ethers were obtained with poor yields owing to the occurrence of excessive oxidation of the ether (entries 1-3). It was found that some oxidants such as IBX, MnO_2_ and TEMPO are ineffective for the ether oxidation process (entries 5-7). None of the chiral secondary amines **IV-VI** were suitable for this reaction in CHCl_3_ while II and III were found to be effective with good stereoselectivity although gave lower yields (entries 8-12). The effect of bases on the processes is evaluated next. In general, the reaction was found to be promoted by bases to furnish the products with good results, whereas NaOAc was the best choice as base for affording the product in the best result (entries 4, 13 and 14). It is also found that reactions performed well in less polar solvents such as CHCl_3_ rather than EtOH and DMF (entries 4 vs 15 and 16). This precedent pointed us toward the possibility of the Michael acceptor pent-2, 4-dienyl ethers (2, *n* = 2) for the oxidative vinylogous iminium activation strategy (entries 17–20). In contrast to cinnamyl tert-butyl ether, the unsaturated iminium ion intermediates were effectively generated in situ from pent-2, 4-dienyl ethyl ether without excessive oxidation by-products (entry 18). Typically, the π-orbital calculation of the LUMO of deconjugated iminium-ion mediated activation indicates that the β-C undergoing a nucleophilic attack is favoured over δ-C enabling 1, 4-addition instead of 1, 6-addition.

Subsequently, some of 3-spirocyclopropyloxindoles and 3-spirocyclopropyl-2-coumaranone **3** were prepared by the general method described above. As revealed in Table 2, remarkably, significant structural variation of allyl ethers, 3-bromooxindoles and 3-bromo-2-coumaranone could be applicable to the powerful one-pot oxidative cascade process to furnish the highly functionalized chiral cyclopropanes 3 in good yields with high levels of enantioselectivity and excellent diastereoselectivity. The electronic nature of the substituents of aromatic systems of **2** has some influence on the outcome. When it comes to cinnamyl tert-butyl ethers, higher reaction yields could be achieved for those with neutral and electron-withdrawing groups than heterocyclic and electron-donating groups (**3a**-**3c**, **3d**-**3e**, **3g**-**3j** and **3k**-**3l**). Furthermore, when the reactant 3-bromooxindoles were changed to 3-bromo-2-coumaranone, the slightly lower reaction yields were observed but with good enantioselectivities (**3a**-**3f**, **3g**-**3l**, **3m**-**3t** and **3** **u**).

**Table 2:** Synthesis of 3-spirocyclopropyl-oxindoles and -benzofuranones.


Entry	X; R^1^; R^2^	n	3	Yield (%)^*b*^	ee(%)^*c*^	dr^*d*^
1	NH/H/Ph	0	**3a**	78	99	90:10
2	NH/H/4-Cl-C_6_H_4_	0	**3b**	76	99	85:15
3	NH/H/4-Br-C_6_H_4_	0	**3c**	82	97	88:12
4	NH/H/4-MeO-C_6_H_4_	0	**3d**	68	99	80:20
5	NH/H/furan-2-yl	0	**3e**	62	99	78:22
6	NH/5-Cl/Ph	0	**3** **f**	70	>99	86:14
7	O/H/Ph	0	**3** **g**	59	92	72:28
8	O/H/4-F-C_6_H_4_	0	**3** **h**	55	97	88:12
9	O/H/4-Cl-C_6_H_4_	0	**3i**	64	98	85:15
10	O/H/4-Br-C_6_H_4_	0	**3j**	65	97	85:15
11	O/H/4-MeO-C_6_H_4_	0	**3k**	53	98	65:35
12	O/H/furan-2-yl	0	**3** **l**	51	98	81:19
13	NH/H/Ph	1	**3** **m**	55	98	88:12
14	NH/H/4-Cl-C_6_H_4_	1	**3n**	64	98	88:12
15	NH/H/4-Br-C_6_H_4_	1	**3o**	58	98	91:9
16	NH/H/4-MeO-C_6_H_4_	1	**3p**	59	98	82:18
17	NH/H/furan-2-yl	1	**3q**	60	99	90:10
18	NH/H/Me	1	**3r**	53	98	88:12
19	NH/5-Cl/Ph	1	**3** **s**	55	97	85:15
20	NH/6-Cl/Ph	1	**3t**	51	99	87:13
21	O/H/Ph	1	**3** **u**	50	99	75:25

Unless stated otherwise, a solution of **1** (1.0 mmol) in CHCl_3_ (6 mL) was added of **2** (1.2 mmol), DDQ (1.2 mmol), NaOAc (2.0 mmol) and catalyst **I** (0.2 mmol) were added following stirred at RT for 24 h. ^b^ Yield of isolated product. ^c^ Determined by chiral HPLC analysis. ^d^ Determined by NMR analyses.

In this paper, the use of ethers as raw materials for the cyclopropanation has the dual advantages of reaction conditions and yields. Wang and co-workers^28^ used aldehyde compounds to realize the cyclopropanation. We found that the yield difference of this type reaction with ether or aldehyde as the substrate is not obvious, because the ether must be oxidized to aldehyde in the reaction process to participate in the reaction. Aldehydes are more reactive and less stable, and the reaction needs to be carried out at 0 °C. The method in this paper can complete the reaction at room temperature. The method of Chen and co-workers^27^ uses α, β-unsaturated acylphosphonates to complete the organocatalytic cyclopropanation reaction. The experimental results show that the acylphosphonates need a higher temperature (90 °C) to participate in the similar cyclopropanation, and the yield of this method is higher when the spiro structures of compounds **3a**, **3c**, **3d**, and **3** **h** are synthesized.

**Fig. 4:**
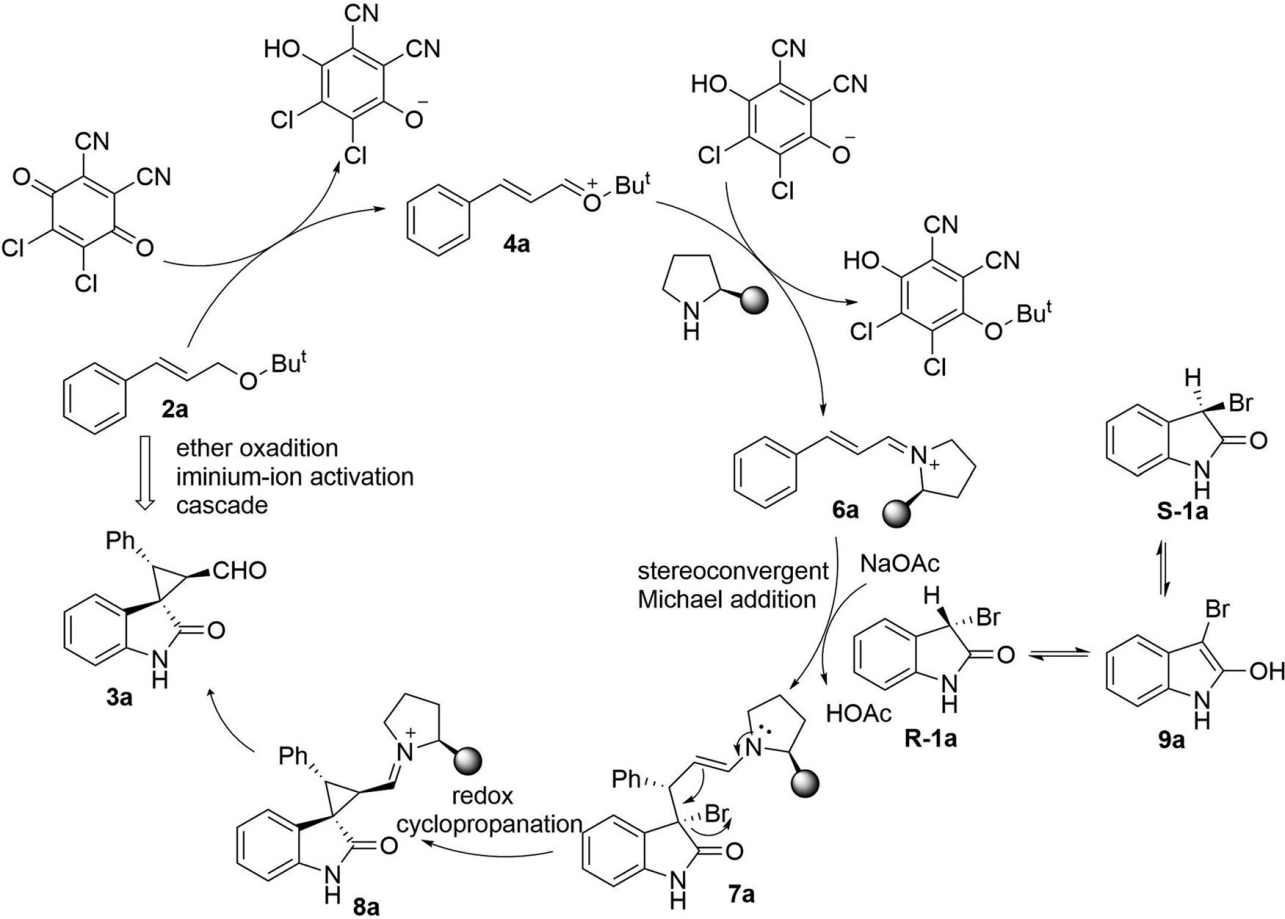
Proposed mechanism. Proposed mechanism about the synthetic strategy of this manuscript and the product exhibiting a single configuration.

A plausible reaction mechanism of the amino catalyst catalysed α, β-spirocyclopropanation reactions of alkyl allyl ether is illustrated in Fig. 4. The formation of oxocarbenium ion **4a** can be initiated by the DDQ-mediated ether oxidation from **2a**, we reason that the generated oxocarbenium ion was active enough and enal can be generated in situ to form the iminium ion intermediate **6a** promoted by aminocatalysis. Then, intermolecular Michael addition happens, then the enamine intermediate **7a** undergoes an intramolecular S_N_2 α-alkylation to produce α, β-spriocyclopropane **3a**.

The isomers **R-1a** and **S-1a** of feedstock **1a** can be interconverted by the enol tautomer **9a** of **1a**. Meanwhile, **R-1a** is the dominant isomer in the reaction with **6a**, and the reaction between **R-1a** and **6a** will lead to the continuous transformation of **S-1a** to **R-1a**, so as to generate the product **3a** with a single configuration (Fig. 4).

In conclusion, we have developed an umpolung ether oxidation iminium-ion activation process to afford spirocyclopropyl benzo-five-membered -lactone and -lactam cores with three adjacent stereo centres and an active aldehyde group which can be transferred to bioactive molecules with warhead targeting to the special drug targets, from 3-bromooxindoles or 3-bromo-2-coumaranone with allyl tert-butyl ethers or pent-2, 4-dienyl ethyl ethers. Compared with the similar methods in the previous literature using aldehyde and acyl phosphonate as the substrate, this method uses ether as the substrate, the reaction conditions are milder, and the synthesis of some compounds has an advantage in yield. And put forward a hypothesis that the product is almost a single configuration. This hypothesis assumes that the racemic benzo five-membered heterocycle undergoes enol tautomerization under the induction of a chiral secondary amine resulting in a single configuration of the product. This study and synthetic application of this strategy provides a new amino catalytic cascade strategy tool for the efficient synthesis of complex drug molecules.

## Methods

### General procedure for synthesis of 3a-3u

A solution of **1** (1.0 mmol) in CHCl_3_ (6 mL) was add of **2** (1.2 mmol), DDQ (1.2 mmol), NaOAc (2.0 mmol) and (*S*)-diphenyl prolinol trimethylsilyl ether (0.2 mmol) following stirred at rt for 24 h. After the reaction completed, the mixture was filtered and the filtrate was removed by vacuum distillation. The crude product was purified by silica gel chromatography to obtain **3a-3u**. See Supplementary Notes 1, 2 for experimental details and compound characterization data. See Supplementary Figs. 1–45 for 1H NMR and 13 C NMR and Supplementary Figs. 46–87 for HPLC spectra.

## Supplementary information

Supplementary Information

## Data Availability

The authors declare that the data supporting the findings of this study are available within the paper and its Supplementary Information file.
