# Supplementary material for: Asymmetric α-spirocyclopropanation of oxindoles and benzofuranones via dynamic kinetic resolution
Source: Commun Chem. 2022 Sep 6;5:106. doi: 10.1038/s42004-022-00695-3 (PMC9814566; doi:10.1038/s42004-022-00695-3)
Supplement: Supplementary file 1 — Supplementary Information [file 42004_2022_695_MOESM1_ESM.pdf]

Supporting Information for

Asymmetric α-Spirocyclopropanation of Oxindoles and Benzofuranones via Dynamic Kinetic Resolution

Yang Hu[a],Jie Yuan[a], Zheyao Li[a], Lin Zhao[a], Xinhong Yu[*a], and Hao Li[*a], Jianhong Zhao[*a]

[a]Shanghai Key Laboratory of New Drug Design and School of Pharmacy and State Key Laboratory of Bioengineering Reactors, East China University of Science & Technology, 200237 Shanghai, China.

Email: xhyu@ecust.edu.cn

Table of Contents

1. Supplementary Note 1 .... S2

2. Supplementary Note 2 .... S3

2.1 Synthesis of starting materials S3

2.2 General procedure for 3 .... S3

2.3 Characterization of products S4

2.4 NMR Spectra S12

2.5 HPLC Spectra S33

Supplementary References S54

1. Supplementary Note 1

All reagents were purchased from commercial suppliers and used without further purification. Nuclear Magnetic Resonance (NMR) spectra were acquired on a Bruker Avance spectrometer, running at 400 MHz for ^{1}H and 100 MHz for ^{13}C, respectively, and chemical shifts are reported in parts permillion (ppm) downfield from TMS, using residual $CDCl_3$ or DMSO-d_6 as an internal standard. Data for ^{1}H are reported as follows: chemical shift (ppm), multiplicity (app = apparent, s = singlet, d = doublet, t = triplet, q = quartet, m = multiplet, comp = complex, br = broad), coupling constant(s) in Hz and integration. Data for ^{13}C NMR are reported as ppm. The number of protons/ carbons given in the parenthesis is the sum over both diastereomers. Mass Spectra were obtained from East China University of Science & Technology mass spectral facility. Reactions were run under an atmosphere of nitrogen unless mentioned otherwise. Purification of the reaction products was carried out column chromatography using silica gel (200-300 mesh). Analytical thin layer chromatography was performed on glass-backed silica gel plates containing ultraviolet-active phosphor and the compounds were visualized either by UV illumination (254 nm), or by means of lime or ink powder.

2. Supplementary Note 2：

2.1 Synthesis of starting materials

3-Bromooxindoles were prepared according to following procedure:

To a stirred solution of oxindoles (10 mmol, 1.0 equiv.) in 30 mL MeCN was added $CuBr_2$ (12 mmol, 1.2 equiv.). After 72h of stirring at rt, the mixture was filtered and filtrate was treated with saturated aqueous $NaHCO_3$ (20mL). The organic material was extracted with EtOAc and dried over $NaSO_4$, and concentrated in vacuo after filtration. Purification by column chromatography on silica gel (petroleum ether/ethylacetate 10/1 to 4/1) to afford pure 3-bromooxindoles.

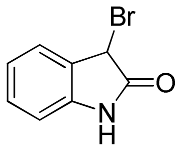

3-Bromooxindole (1a). ^{1}H NMR (400 MHz, $CDCl_3$): δ 9.07 (br, 1H), 7.41 (d, *J* = 7.5 Hz, 1H), 7.31 (t, *J* = 7.6 Hz, 1H), 7.11 (dt, *J* = 7.6, 0.8 Hz, 1H), 6.95 (d, *J* = 7.8 Hz, 1H), 5.32 (s, 1H). ^{13}C NMR (101 MHz, $CDCl_3$): δ 175.0, 141.1, 130.5, 126.7, 126.3, 123.4, 110.7, 39.1. HRMS (EI+) *m/z* calcd. for C_8H_6BrNO: 210.9633; found: 210.9634.

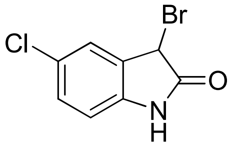

5-Chloro-3-bromooxindole (1b). ^{1}H NMR (400 MHz, $CDCl_3$): *δ* 8.71 (s, 1H), 7.31 (s, 1H), 6.80 (d, *J* = 8.3 Hz, 1H), 7.20 (d, *J* = 8.4 Hz, 1H), 5.19 (s, 1H). ^{13}C NMR (101 MHz, $CDCl_3$): *δ* 174.2, 139.4, 130.5, 128.9, 128.3, 126.7, 111.6, 38.1. HRMS (EI+) *m/z* calcd. for $C_8H_5BrClNO$: 244.9243; found: 244.9246.

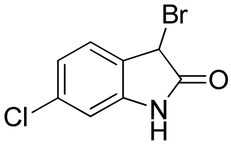

6-Chloro-3-bromooxindole (1c). ^{1}H NMR (400 MHz, $CDCl_3$): *δ* 8.94 (br, 1H), 7.33 (d, *J* = 8.1 Hz, 1H), 7.10 (dd, *J* = 8.1, 1.8 Hz, 1H), 6.98 (d, *J* = 1.7 Hz, 1H), 5.28 (s, 1H). ^{13}C NMR (101 MHz, $CDCl_3$): *δ* 174.8, 142.0, 136.3, 127.2, 125.0, 123.6, 111.4, 38.1. HRMS (EI+) *m/z* calcd. for $C_8H_5BrClNO$: 244.9243; found: 244.9244.

3-Bromocoumaran-2-one **1d** was prepared following literature procedure.[1]

2.2 General procedure for 3

A solution of **1** (1.0 mmol) in $CHCl_3$ (6mL) was add of **2** (1.2 mmol), DDQ (1.2mmol), NaOAc (2.0 mmol) and (*S*)-diphenyl prolinol trimethylsilyl ether (0.2 mmol) following stirred at rt for 24 h. After the reaction completed, the mixture was filtered and the filtrate was removed by vacuum distillation. The crude product was purified by column chromatography on silica gel.

2.3 Characterization of products

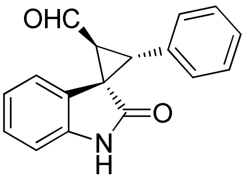

(1*S*,2*S*,3*R*)-2'-oxo-3-phenylspiro[cyclopropane-1,3'-indoline]-2-carbaldehyde 3a. Following the general procedure, **3a** was obtained after silica gel column chromatography (petroleum ether/ethylacetate 5/1) as a white solid in 78% yield (204 mg) with d.r. 90/10 and ee 99% (HPLC: Chiralpak AD-3, *i*-PrOH/hexane = 25/75, flow rate = 1.0 mL/min, λ= 254 nm; t_{major} = 11.5 min and t_{minor} = 10.22 min). ^{1}H NMR ($CDCl_3$, 400 MHz): δ 9.87 (d, *J* = 3.0 Hz, 1H), 8.81 (br, 1H), 7.29-7.28 (m, 4H), 7.27-7.24 (m, 2H), 7.21 (d, *J* = 7.7 Hz, 1H), 7.03 (t, *J* = 7.7 Hz, 1H), 6.80 (d, *J* = 7.8 Hz, 1H), 3.95 (d, *J* = 8.2 Hz, 1H), 3.55 (dd, *J* = 3.0, 8.2 Hz, 1H). ^{13}C NMR ($CDCl_3$, 101 MHz): δ195.4, 173.5, 141.3, 132.4, 129.2, 128.2, 127.9, 127.7, 126.1, 122.4, 122.3, 110.2, 44.2, 41.9, 39.6. HRMS (EI+) m/z calcd. for $C_{17}H_{13}NO_2$: 263.0946; found: 263.09455.

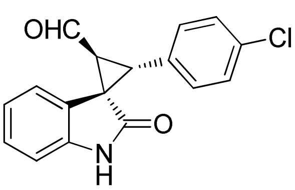

(1*S*,2*R*,3*S*)-2-(4-chlorophenyl)-2'-oxospiro[cyclopropane-1, 3'-indoline]-3-carbaldehyde 3b. Following the general procedure, **3b** was obtained after silica gel column chromatography (petroleum ether/ethylacetate 5/1) as a white solid in 76% yield (225 mg) with d.r. 85/15 and ee >99% (HPLC: Chiralpak AD-3, *i*-PrOH/hexane = 25/75, flow rate = 1.0 mL/min, λ= 254 nm; t_{major} = 12.15 min and t_{minor} = 13.37 min). ^{1}H NMR ($CDCl_3$, 400 MHz): δ 9.89 (d, *J* = 2.7 Hz, 1H), 8.14 (br, 1H), 7.27-7.22 (m, 6H), 7.06 (t, *J* = 7.6 Hz, 1H), 6.87 (d, *J* = 7.9 Hz, 1H), 3.91 (d, *J* = 8.2 Hz, 1H), 3.55 (dd, *J* = 2.6, 8.1 Hz, 1H). ^{13}C NMR ($CDCl_3$, 101 MHz): δ 195.1, 172.8, 140.8, 133.7, 130.9, 130.5, 128.4, 128.2, 125.8, 122.5, 110.1, 44.0, 41.8, 38.6. HRMS (EI+) m/z calcd. for $C_{17}H_{12}ClNO_2$: 297.0557; found: 297.0559.

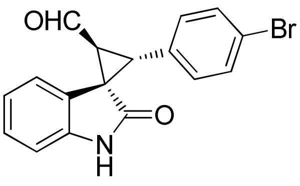

(1*S*,2*R*,3*S*)-2-(4-bromophenyl)-2'-oxospiro[cyclopropane-1, 3'-indoline]-3-carbaldehyde 3c. Following the general procedure, **3c** was obtained after silica gel column chromatography (petroleum ether/ethylacetate 5/1) as a white solid in 82% yield (278 mg) with d.r. 88/12 and ee 97% (HPLC: Chiralpak AD-3, *i*-PrOH/hexane = 25/75, flow rate = 1.0 mL/min, λ= 254 nm; t_{major} = 12.62 min and t_{minor} = 13.54 min). ^{1}H NMR ($CDCl_3$, 400 MHz): δ 9.89 (d, *J* = 2.6 Hz, 1H), 8.21 (br, 1H), 7.27-7.25 (m, 2H), 7.43 (d, *J* = 8.3 Hz, 2H), 7.18 (d, *J* = 8.3 Hz, 2H), 7.06 (t, *J* = 7.6 Hz, 1H), 6.87 (d, *J* = 7.7 Hz, 1H), 3.89 (d, *J* = 8.2 Hz, 1H), 3.55 (dd, *J* = 8.2, 2.6 Hz, 1H). ^{13}C NMR ($CDCl_3$, 101 MHz): δ 195.1, 172.9, 140.8, 131.4, 131.3, 130.9, 128.2, 125.7, 122.5, 121.9, 110.1, 43.9, 41.7, 38.7. HRMS (EI+) m/z calcd. for $C_{17}H_{12}BrNO_2$: 341.0051; found: 341.0050.

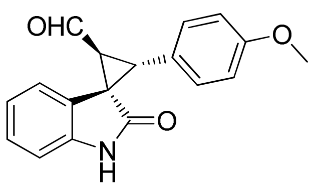

(1*S*,2*S*,3*R*)-3-(4-methoxyphenyl)-2'-oxospiro[cyclopropan e-1,3'-indoline]-2-carbaldehyde 3d. Following the general procedure, **3d** was obtained after silica gel column chromatography (petroleum ether/ethylacetate 4/1) as a white solid in 68% yield (199 mg) with d.r. 80/20 and ee 99% (HPLC: Chiralpak AD-3, *i*-PrOH/hexane = 25/75, flow rate =

1.0 mL/min, λ= 254 nm; t_{major} = 18.59 min and t_{minor} = 21.37 min). ^{1}H NMR ($CDCl_3$, 400 MHz): δ 9.80 (d, *J* = 3.1 Hz, 1H), 8.54 (br, 1H), 7.19-7.13 (m, 4H), 6.97 (dt, *J* = 7.6, 0.6 Hz, 1H), 6.78-6.75 (m, 3H), 3.84 (d, *J* = 8.2 Hz, 1H), 3.69 (s, 3H), 3.46 (dd, *J* = 8.2, 3.1 Hz, 1H).□^{13}C NMR ($CDCl_3$, 101 MHz): δ 195.6, 159.1, 141.0, 130.9, 130.3, 127.9, 126.2, 124.2, 122.3, 114.1, 113.6, 110.2, 55.2, 44.4, 41.9, 39.3. HRMS (EI+) m/z calcd. for $C_{18}H_{15}NO_3$: 293.1052; found: 293.1054.

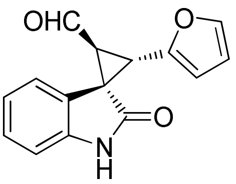

(1*S*,2*S*,3*S*)-3-(furan-2-yl)-2'-oxospiro[cyclopropane-1,3'-indoline]-2-carbaldehyde 3e. Following the general procedure, **3e** was obtained after silica gel column chromatography (petroleum ether/ethylacetate 6/1) as a white solid in 62% yield (156 mg) with d.r. 78/22 and ee 96% (HPLC: Chiralpak AD-3, *i*-PrOH/hexane = 20/80, flow rate = 1.0 mL/min, λ= 254 nm; t_{major} = 10.72 min and t_{minor} = 9.32 min). ^{1}H NMR ($CDCl_3$, 400 MHz): δ 9.86 (d, *J* = 2.5 Hz, 1H), 8.13 (br, 1H), 7.34 (s, 1H), 7.25-7.21 (m, 2H), 7.04 (t, *J* = 7.7 Hz, 1H), 6.91 (d, *J* = 7.8 Hz, 1H), 6.37 (s, 2H), 3.81 (d, *J* =8.0 Hz, 1H), 3.57 (dd, *J* = 8.0, 2.4 Hz, 1H).□^{13}C NMR ($CDCl_3$, 101 MHz): δ 194.5, 172.5, 146.8, 142.3, 140.8, 128.2, 125.3, 122.5, 122.4, 110.7, 110.0, 109.3, 43.0, 40.8, 31.7. HRMS (EI+) m/z calcd. for $C_{15}H_{11}NO_3$: 253.0739; found: 253.0740.

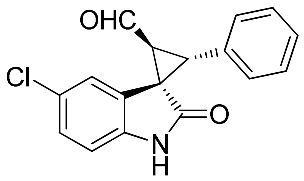

(1*S*,2*S*,3*R*)-5'-chloro-2'-oxo-3-phenylspiro[cyclopropane-1,3'-indoline]-2-carbaldehyde 3f. Following the general procedure, **3f** was obtained after silica gel column chromatography (petroleum ether/ethylacetate 4/1) as a white solid in 70% yield (208 mg) with d.r. 86/22 and ee >99% (HPLC: Chiralpak AD-3, *i*-PrOH/hexane = 25/75, flow rate = 1.0 mL/min, λ= 254 nm; t_{major} = 9.28 min and t_{minor} = 10.01 min). ^{1}H NMR ($CDCl_3$, 400 MHz): δ 9.92 (d, *J* = 2.5 Hz, 1H), 8.53 (br, 1H), 7.36-7.34 (m, 1H), 7.29-7.26 (m, 5H), 7.20-7.19 (1H), 6.78 (d, *J* = 8.3 Hz, 1H), 3.94 (d, *J* = 8.3 Hz, 1H), 3.62 (dd, *J* = 8.3, 2.5 Hz, 1H).□^{13}C NMR ($CDCl_3$, 101 MHz): δ 195.0, 172.6, 139.4, 131.9, 129.1, 128.3, 128.0, 127.9, 127.8, 127.7, 123.0, 110.8, 44.0, 41.9, 40.1. HRMS (EI+) m/z calcd. for $C_{17}H_{12}ClNO_2$: 297.0557; found: 297.0561.

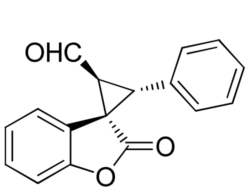

(1'*S*,2'*S*,3'*R*)-2-oxo-3'-phenyl-2H-spiro[benzofuran-3,1'-cyclopropane]-2'-carbaldehyde 3g. Following the general procedure, **3g** was obtained after silica gel column chromatography (petroleum ether/ethylacetate 10/1) as a yellow solid in 59% yield (155 mg) with d.r. 72/28 and ee >99% (HPLC: Chiralpak AD-3, *i*-PrOH/hexane = 20/80, flow rate = 1.0 mL/min, λ= 254 nm; t_{major} = 11.14 min and t_{minor} = 10.65 min). ^{1}H NMR (400 MHz, CDCl3) δ 9.84 (d, *J* = 2.4 Hz, 1H), 7.30 (dd, *J* = 14.3, 6.2 Hz, 7H), 7.15 – 7.07 (m, 2H), 3.96 (d, *J* = 8.4 Hz, 1H), 3.63 (dd, *J* = 8.4, 2.4 Hz, 1H). ^{13}C NMR ($CDCl_3$, 101 MHz): δ 194.3, 171.3, 153.8, 131.1, 129.6, 129.0, 128.9, 128.5, 128.3, 124.3, 122.7, 110.9, 44.3, 40.5, 39.6. HRMS (EI+) m/z calcd. for $C_{17}H_{12}O_3$: 264.0786; found: 264.0787.

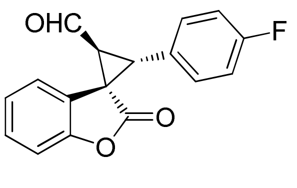

(1'*S*,2'*R*,3'*S*)-2'-(4-fluorophenyl)-2-oxo-2H-spiro[benzofuran-3,1'-cyclopropane]-3'-carbaldehyde 3h. Following the general procedure, **3h** was obtained after silica gel column chromatography (petroleum ether/ethylacetate 10/1) as a

yellow solid in 55% yield (156 mg) with d.r. 88/12 and ee 98% (HPLC: Chiralpak AD-3, *i*-PrOH/hexane = 20/80, flow rate = 1.0 mL/min, λ= 254 nm; t_{major} = 10.57 min and t_{minor} = 12.10 min). ^{1}H NMR (400 MHz, $CDCl_3$) δ 9.91 (d, *J* = 2.3 Hz, 1H), 7.32 (d, *J* = 7.5 Hz, 2H), 7.29 – 7.24 (m, 3H), 7.18 – 7.13 (m, 3H), 7.02 (t, *J* = 8.7 Hz, 3H), 3.95 (d, *J* = 8.3 Hz, 1H), 3.63 (dd, *J* = 8.3, 2.3 Hz, 1H). ^{13}C NMR ($CDCl_3$, 101 MHz): δ 194.05, 171.31, 162.54 (d, $^{1}J_{CF}$ = 246 Hz), 153.80, 130.72 (d, $^{3}J_{CF}$ = 8 Hz) 128.94, 126.93 (d, $^{4}J_{CF}$ = 3 Hz), 124.36, 124.21, 122.66, 115.51 (d, $^{2}J_{CF}$ = 21 Hz), 110.94, 53.38, 44.42, 39.51. HRMS (EI+) m/z calcd. for $C_{17}H_{11}FO_3$: 282.0692; found: 282.0695.

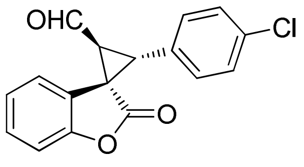

(1'*S*,2'*R*,3'*S*)-2'-(4-chlorophenyl)-2-oxo-2H-spiro[benzofuran-3,1'-cyclopropane]-3'-carbaldehyde 3i. Following the general procedure, **3i** was obtained after silica gel column chromatography (petroleum ether/ethylacetate 10/1) as a yellow solid in 64% yield (191 mg) with d.r. 85/15 and ee 98% (HPLC: Chiralpak AD-3, *i*-PrOH/hexane = 20/80, flow rate = 1.0 mL/min, λ= 254 nm; t_{major} = 10.69 min and t_{minor} = 13.30 min). ^{1}H NMR ($CDCl_3$, 400 MHz): δ 9.92 (s, 1H), 7.36-7.31 (m, 4H), 7.25-7.23 (m, 2H), 7.20-7.16 (m, 2H), 3.94 (d, *J* = 8.1 Hz, 1H), 3.64 (d, *J* = 8.2 Hz, 1H). ^{13}C NMR ($CDCl_3$, 101 MHz): δ 194.0, 171.3, 153.8, 134.2, 130.4, 129.7, 129.0, 128.7, 124.4, 124.1, 122.7, 111.0, 44.2, 39.5, 39.4. HRMS (EI+) m/z calcd. for $C_{17}H_{11}ClO_3$: 298.0397; found: 298.0396.

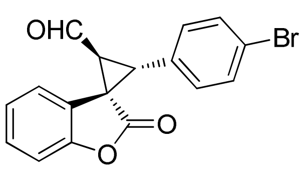

(1'*S*,2'*R*,3'*S*)-2'-(4-bromophenyl)-2-oxo-2H-spiro[benzofuran-3,1'-cyclopropane]-3'-carbaldehyde 3j. Following the general procedure, **3j** was obtained after silica gel column chromatography (petroleum ether/ethylacetate 9/1) as a yellow solid in 65% yield (221 mg) with d.r. 85/15 and ee 97% (HPLC: Chiralpak AD-3, *i*-PrOH/hexane = 20/80, flow rate = 1.0 mL/min, λ= 254 nm; t_{major} = 15.65 min and t_{minor} = 11.45 min). ^{1}H NMR ($CDCl_3$, 400 MHz): δ 9.92 (d, *J* = 2.2 Hz, 1H), 7.48-7.44 (m, 2H), 7.37-7.31 (m, 2H), 7.20-7.15 (m, 4H), 3.92 (d, *J* = 8.3 Hz, 1H), 3.64 (dd, *J* = 8.3, 2.2 Hz, 1H). ^{13}C NMR ($CDCl_3$, 101 MHz): δ 193.6, 171.8, 154.1, 149.1, 133.5, 131.9, 129.4, 129.2, 127.7, 125.3, 124.5, 123.9, 122.9, 111.0, 44.2, 40.0, 37.0. HRMS (EI+) m/z calcd. for $C_{17}H_{11}BrO_3$: 341.9892; found: 341.9895.

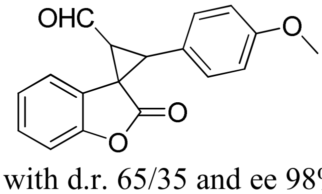

3'-(4-methoxyphenyl)-2-oxo-2H-spiro[benzofuran-3,1'-cyclopropane]-2'-carbaldehyde [(1'*S*,2'*S*,3'*R*)/(1'*R*,2'*S*,3'*R*) 65/35 mixture] 3k. Following the general procedure, **3k** was obtained after silica gel column chromatography (petroleum ether/ethylacetate 8/1) as a yellow oil in 53% yield (161 mg) with d.r. 65/35 and ee 98% (HPLC: Chiralpak AD-3, *i*-PrOH/hexane = 20/80, flow rate = 1.0 mL/min, λ= 254 nm; t_{major} = 18.08 min and t_{minor} = 16.38 min). ^{1}H NMR (400 MHz, $CDCl_3$) δ 9.92 (d, *J* = 2.4 Hz, 1H), 7.33 (t, *J* = 6.9 Hz, 2H), 7.22 (d, *J* = 8.7 Hz, 2H), 7.18 – 7.11 (m, 2H), 6.86 (d, *J* = 8.6 Hz, 2H), 3.94 (d, *J* = 8.3 Hz, 1H), 3.80 (s, 3H), 3.63 (dd, *J* = 8.3, 2.4 Hz, 1H). ^{13}C NMR ($CDCl_3$, 101 MHz): δ 196.7, 194.4, 173.9, 171.4, 159.5, 153.7, 130.8, 130.1, 128.7, 124.6, 124.3, 124.0, 123.7, 123.0, 122.8, 122.6, 121.5, 114.3, 113.9, 110.9, 110.8, 45.0, 44.6, 40.3, 39.6, 39.5, 38.2. HRMS (EI+) m/z calcd. for $C_{18}H_{14}O_4$: 294.0892; found: 294.0893.

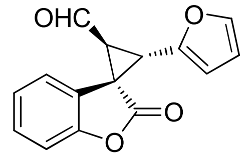

(1'*S*,2'*S*,3'*S*)-3'-(furan-2-yl)-2-oxo-2H-spiro[benzofuran-3,1'-cyclopropane]-2'-carbaldehyde 3l. Following the general procedure, **3l** was obtained after silica gel column chromatography (petroleum ether/ethylacetate 12/1) as a yellow solid in 51% yield (129 mg) with d.r. 81/19 and ee 98% (HPLC: Chiralpak AD-3, *i*-PrOH/hexane = 25/75, flow rate = 1.0 mL/min, λ= 254 nm; t_{major} = 10.87 min and t_{minor} = 9.93 min). ^{1}H NMR (400 MHz, $CDCl_3$) δ 9.90 (d, *J* = 2.0 Hz, 1H), 7.37 (s, 1H), 7.34 (s, 1H), 7.30 (d, *J* = 13.6 Hz, 1H), 7.20 – 7.13 (m, 2H), 6.48 – 6.32 (m, 2H), 3.86 (d, *J* = 8.1 Hz, 1H), 3.66 (dd, *J* = 8.1, 2.0 Hz, 1H). ^{13}C NMR ($CDCl_3$, 101 MHz): δ 193.4, 170.9, 153.8, 145.7, 142.8, 129.0, 124.3, 123.6, 122.7, 110.9, 110.8, 109.8, 43.4, 38.6, 32.7. HRMS (EI+) m/z calcd. for $C_{15}H_{10}O_4$: 254.0579; found: 254.0580.

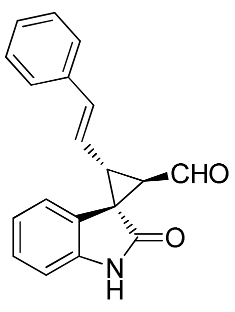

(1*R*,2*R*,3*R*)-2'-oxo-3-((*E*)-styryl)spiro[cyclopropane-1,3'-indolin]-2-carbaldehyde 3m. Following the general procedure, **3m** was obtained after silica gel column chromatography (petroleum ether/ethylacetate 4/1) as a white solid in 55% yield (158 mg) with d.r. 88/12 and ee 98% (HPLC: Chiralpak AD-3, *i*-PrOH/hexane = 30/70, flow rate = 1.0 mL/min, λ= 254 nm; t_{major} = 31.93 min and t_{minor} = 12.70 min). ^{1}H NMR (DMSO-d_6, 400 MHz) δ 10.85 (s, 1H), 9.70 (d, *J* = 4.5 Hz, 1H), 7.29-7.38 (m, 5H), 7.19-7.25 (m, 2H), 6.97 (t, *J* = 7.5 Hz, 1H), 6.91 (d, *J* = 7.7 Hz, 1H), 6.79 (d, *J* = 16.0 Hz, 1H), 6.61 (dd, *J* = 16.0, 9.2 Hz, 1H), 3.62-3.66 (m, 1H), 3.03 (dd, *J* = 7.0, 4.7 Hz, 1H). ^{13}C NMR ($CDCl_3$, 101 MHz): δ 195.0, 174.2, 140.9, 136.5, 134.2, 128.6, 128.0, 127.8, 126.3, 125.7, 122.5, 122.4, 122.3, 110.2, 46.0, 42.0, 39.2. HRMS (ESI+) *m/z* calcd. for $C_{19}H_{15}NO_2$ $[M+H]^+$: 289.1103; found: 289.1099.

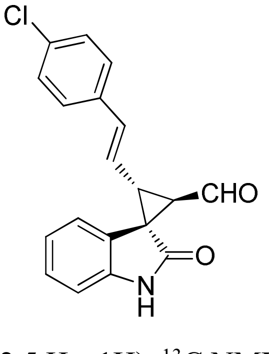

(1*R*,2*R*,3*R*)-2'-oxo-3-((*E*)-4-chlorostyryl)spiro[cyclopropane-1,3'-indolin]-2-carbaldehyde 3n. Following the general procedure, **3n** was obtained after silica gel column chromatography (petroleum ether/ethylacetate 3/1) as a white solid in 64% yield (206 mg) with d.r. 88/12 and ee 98% (HPLC: Chiralpak AD-3, *i*-PrOH/hexane = 30/70, flow rate = 1.0 mL/min, λ= 254 nm; t_{minor} = 13.72 min and t_{major} = 37.77 min). ^{1}H NMR (400 MHz, $CDCl_3$) δ ppm 9.74 (d, *J* = 2.7 Hz, 1H), 8.50 (br, 1H), 7.19-7.14 (m, 6H), 6.97 (t, *J* = 7.6 Hz, 1H), 6.87 (d, *J* = 7.7 Hz, 1H), 6.69-6.57 (m, 2H), 3.33 (t, *J* = 7.4 Hz, 1H), 3.23 (dd, *J* = 7.3, 2.5 Hz, 1H). ^{13}C NMR (101MHz, $CDCl_3$) δ ppm 193.8, 173.1, 139.8, 134.0, 132.4, 131.9, 127.7, 127.0, 126.5, 124.6, 122.0, 121.6, 121.5, 109.1, 44.8, 40.9, 37.9. HRMS (EI+) *m/z* calcd. for $C_{19}H_{14}ClNO_2$: 323.0713; found: 323.0712.

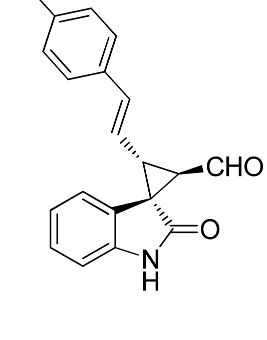

(1*R*,2*R*,3*R*)-2'-oxo-3-((*E*)-4-bromostyryl)spiro[cyclopropane-1,3'-indolin]-2-carbaldehyde 3o. Following the general procedure (12h), **3o** was obtained after silica gel column chromatography (petroleum ether/ethylacetate 3/1) as a white solid in 58% yield (213 mg) with d.r. 91/9 and ee 98% (HPLC: Chiralpak AD-3, *i*-PrOH/hexane = 30/70, flow rate = 1.0 mL/min, λ= 254 nm; t_{minor} = 14.21 min and t_{major} = 46.64 min).

^{1}H NMR (400 MHz, $CDCl_3$) δ ppm 9.79 (d, *J* = 3.1 Hz, 1H), 9.11 (br, 1H), 7.37 (t, *J* = 7.2 Hz, 2H), 7.26-7.22 (m, 2H), 7.19 (d, *J* =7.1 Hz, 2H), 7.04 (t, *J* = 7.6 Hz, 1H), 6.95 (d, *J* = 7.7 Hz, 1H), 6.68 (dd, *J* = 15.9, 8.7 Hz, 1H), 6.60 (d, *J* = 16.0 Hz, 1H), 3.43-3.37 (m, 1H), 3.29 (dd, *J* = 7.4, 3.1 Hz, 1H). ^{13}C NMR (101MHz, $CDCl_3$) δ ppm 194.8, 174.4, 141.0, 135.5, 132.9, 131.7, 128.0, 127.8, 125.6, 123.2, 122.5, 122.4, 121.5, 110.2, 45.8, 41.9, 38.9. HRMS (EI+) *m/z* calcd. for $C_{19}H_{14}BrNO_2$: 367.0208; found: 367.0205.

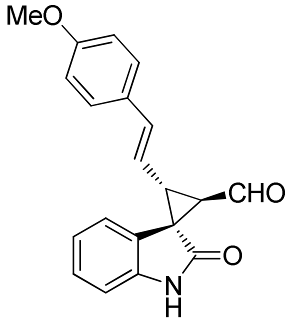

(1*R*,2*R*,3*R*)-2'-oxo-3-((*E*)-4-methoxystyryl)spiro[cyclopropane-1,3'-indolin]-2-carbaldehyde 3p. Following the general procedure (18h), **3p** was obtained after silica gel column chromatography (petroleum ether/ethylacetate 5/1) as a white solid in 59% yield (188 mg) with d.r. 82/18 and ee 98% (HPLC: Chiralpak AD-3, *i*-PrOH/hexane = 30/70, flow rate = 1.0 mL/min, λ= 254 nm; t_{minor} = 20.87 min and t_{major} = 47.62 min). ^{1}H NMR (400 MHz, $CDCl_3$) δ ppm 9.82 (d, *J* = 3.3 Hz, 1H), 9.06 (br, 1H), 7.31 (d, *J* = 8.7 Hz, 2H), 7.23 (dt, *J* = 7.9, 1.3 Hz, 2H), 7.08-7.03 (m, 1H), 6.97 (d, *J* = 7.7 Hz, 1H), 6.84 (d, *J* = 8.7 Hz, 2H), 6.65 (d, *J* = 15.9 Hz, 1H), 6.56 (dd, *J* = 15.9, 8.8 Hz, 1H), 3.81 (s, 3H), 3.43 (dd, *J* = 8.7, 7.6 Hz, 1H), 3.30 (dd, *J* = 7.5, 3.3 Hz, 1H). ^{13}C NMR (101 MHz, $CDCl_3$) δ ppm 195.1, 174.5, 159.4, 141.0, 133.7, 129.4, 127.9, 127.6, 125.9, 122.5, 122.4, 119.9, 114.0, 110.2, 55.3, 46.1, 42.1, 39.5. HRMS (EI+) *m/z* calcd. for $C_{20}H_{17}NO_3$: 319.1208; found: 319.1209.

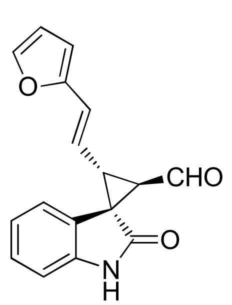

(1*R*,2*R*,3*R*)-2'-oxo-3-((*E*)-2-(furan-2-yl)vinyl)spiro[cyclopropane-1,3'-indolin]-2-carbaldehyde 3q. Following the general procedure (12h), **3q** was obtained after silica gel column chromatography (petroleum ether/ethylacetate 5/1) as a white solid in 60% yield (166 mg) with d.r. 90/10 and ee >99% (HPLC: Chiralpak AD-3, *i*-PrOH/hexane = 30/70, flow rate = 1.0 mL/min, λ= 254 nm; t_{minor} = 11.31 min and t_{major} = 32.08 min). ^{1}H NMR (400 MHz, $CDCl_3$) δ ppm 9.83 (d, *J* = 1.3 Hz, 1H), 8.39 (br, 1H), 7.35 (s, 1H), 7.27-7.23 (m, 2H), 7.06 (t, *J* = 7.6, 1H), 6.96 (d, *J* = 7.8 Hz, 1H), 6.62 (dd, *J* = 15.8, 9.0 Hz, 1H), 6.51 (d, *J* = 15.9 Hz, 1H), 6.37 (s, 1H), 6.25 (s, 1H), 3.37 (t, *J* = 8.2 Hz, 1H), 3.32 (d, *J* = 7.3 Hz, 1H). ^{13}C NMR (101 MHz, $CDCl_3$) δ ppm 194.9, 173.8, 152.2, 142.2, 140.8, 128.0, 125.7, 122.6, 122.4, 122.3, 120.8, 111.3, 110.1, 108.0, 45.9, 42.1, 39.0. HRMS (EI+) *m/z* calcd. for $C_{17}H_{13}NO_3$: 279.0895; found: 279.0896.

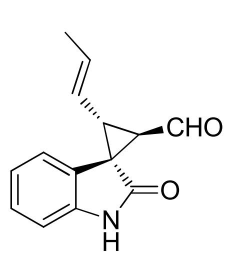

(1*R*,2*R*,3*R*)-2'-oxo-3-((*E*)-prop-1-en-1-yl)spiro[cyclopropane-1,3'-indolin]-2-carbaldehyde 3r. Following the general procedure (12h), **3r** was obtained after silica gel column chromatography (petroleum ether/ethylacetate 4/1) as a light yellow oil in 53% yield (120 mg) with d.r. 88/12 and ee 98% (HPLC: Chiralpak AD-3, *i*-PrOH/hexane = 20/80, flow rate = 1.0 mL/min, λ= 254 nm; t_{minor} = 7.16 min and t_{major} = 9.52 min). ^{1}H NMR (400 MHz, DMSO-*d*$_6$) δ ppm 10.81 (s, 1H), 9.65 (d, *J* = 4.8 Hz, 1H), 7.27 (d, *J* = 7.6 Hz, 1H), 7.20 (t, *J* = 7.7 Hz, 1H), 6.96 (t, *J* = 7.6 Hz, 1H), 6.91 (d, *J* = 7.7 Hz, 1H), 5.90-5.77 (m, 2H), 3.46-3.40 (m, 1H), 2.82 (dd, *J* = 7.2, 4.8 Hz,

1H), 1.67 (d, J = 4.2 Hz, 3H). ^{13}C NMR (101 MHz, DMSO-d_6) δ ppm 197.7, 173.6, 142.4, 130.0, 128.1, 126.3, 124.3, 122.7, 121.7, 110.2, 45.9, 40.7, 37.8, 18.3. HRMS (EI+) *m/z* calcd. for $C_{17}H_{13}NO_3$: 227.0946; found: 227.0947.

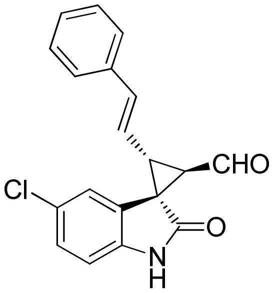

(1*R*,2*R*,3*R*)-5'-chloro-2'-oxo-3-((*E*)-styryl)spiro[cyclopropane-1,3'-indolin]-2-carbaldehyde 3s. Following the general procedure (12h), **3s** was obtained after silica gel column chromatography (petroleum ether/ethylacetate 5/1) as a white solid in 55% yield (178 mg) with d.r. 85/15 and ee 97% (HPLC: Chiralpak AD-3, *i*-PrOH/hexane = 30/70, flow rate = 1.0 mL/min, λ= 254 nm; t_{minor} = 9.36 min and t_{major} = 18.08 min). ^{1}H NMR (500 MHz, DMSO-d_6) δ ppm 10.99 (s, 1H), 9.73 (d, J = 4.0 Hz, 1H), 7.40 (d, J = 1.8 Hz, 1H), 7.36-7.34 (m, 4H), 7.30-7.29 (m, 1H), 7.26 (d, J = 2.2 Hz, 1H), 6.92 (d, J = 8.3 Hz, 1H), 6.82 (d, J = 16.1 Hz, 1H), 6.59 (dd, J = 16.0, 9.1 Hz, 1H), 3.69 (dd, J = 9.1, 7.6 Hz, 1H), 3.13 (dd, J = 7.4, 4.1 Hz, 1H). ^{13}C NMR (101 MHz, DMSO-d_6) δ ppm 197.5, 173.4, 141.5, 136.9, 133.9, 129.2, 128.3, 128.2, 127.9, 126.5, 125.9, 123.4, 123.0, 111.6, 46.0, 41.5, 38.5. HRMS (EI+) *m/z* calcd. for $C_{19}H_{14}ClNO_2$: 323.0713; found: 323.0712.

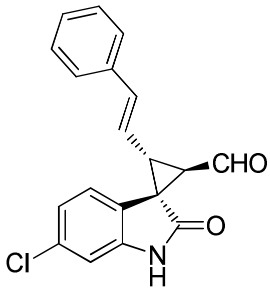

(1*S*,2*R*,3*S*)-6'-chloro-2'-oxo-3-((*E*)-styryl)spiro[cyclopropane-1,3'-indolin]-2-carbaldehyde 3t. Following the general procedure, **3t** was obtained after silica gel column chromatography (petroleum ether/ethylacetate 5/1) as a white solid in 51% yield (165 mg) with d.r. 85/15 and ee >99% (HPLC: Chiralpak AD-3, *i*-PrOH/hexane = 30/70, flow rate = 1.0 mL/min, λ= 254 nm; t_{minor} = 12.58 min and t_{major} = 20.84 min). ^{1}H NMR (400 MHz, $CDCl_3$) δ ppm 9.85 (d, J = 2.2 Hz, 1H), 8.98 (br, 1H), 7.38 (d, J = 7.9 Hz, 2H), 7.32 (t, J = 7.4 Hz, 2H), 7.24 (t, J = 8.1 Hz, 2H), 7.04 (d, J = 8.2 Hz, 1H), 7.00(s, 1H), 6.70-6.68 (m, 2H), 3.43 (t, J = 7.7 Hz, 1H), 3.37 (dd, J = 7.5, 2.1 Hz, 1H). ^{13}C NMR (101 MHz, $CDCl_3$) δ ppm 194.9, 174.5, 141.9, 136.4, 134.5, 133.8, 128.7, 127.9, 126.3, 124.1, 123.7, 122.5, 121.8, 110.8, 45.8, 42.0, 39.5. HRMS (EI+) *m/z* calcd. for $C_{19}H_{14}ClNO_2$: 323.0713; found: 323.0715.

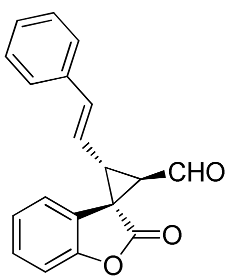

(1'*R*,2'*R*,3'*R*)-2-oxo-3'-((*E*)-styryl)-2H-spiro[benzofuran-3,1'-cyclopropane]-2'-carbaldehyde 3u. Following the general procedure, **3u** was obtained after silica gel column chromatography (petroleum ether/ethylacetate 10/1) as a white solid in 55% yield (159 mg) with d.r. 85/15 and ee 99% (HPLC: Chiralpak AD-3, *i*-PrOH/hexane = 20/80, flow rate = 1.0 mL/min, λ= 254 nm; t_{minor} = 12.60 min and t_{major} = 13.28 min). ^{1}H NMR (400 MHz, $CDCl_3$) δ ppm 9.84 (d, J = 2.3 Hz, 1H), 7.38-7.35 (m, 2H), 7.34-7.28 (m, 4H), 7.27-7.23 (m, 1H), 7.18-7.13 (m, 2H), 6.74 (d, J = 15.9 Hz, 1H), 6.54 (dd, J = 15.9, 9.2 Hz, 1H), 3.45 (dd, J = 9.1, 7.7 Hz, 1H), 3.37 (dd, J = 7.6, 2.3 Hz, 1H). ^{13}C NMR (101 MHz, $CDCl_3$) δ ppm 193.9, 172.5, 153.8, 136.2, 135.4, 128.8, 128.7, 128.1, 126.4, 124.3, 124.1, 122.8, 120.9, 110.9, 46.2, 39.8, 39.6. HRMS (EI+) *m/z* calcd. for $C_{19}H_{14}O_3$: 290.0943; found: 290.0942.

Relative stereochemistry of spirocyclopropyloxindole 3c was determined by NOE analysis. The fallowing results were obtained:

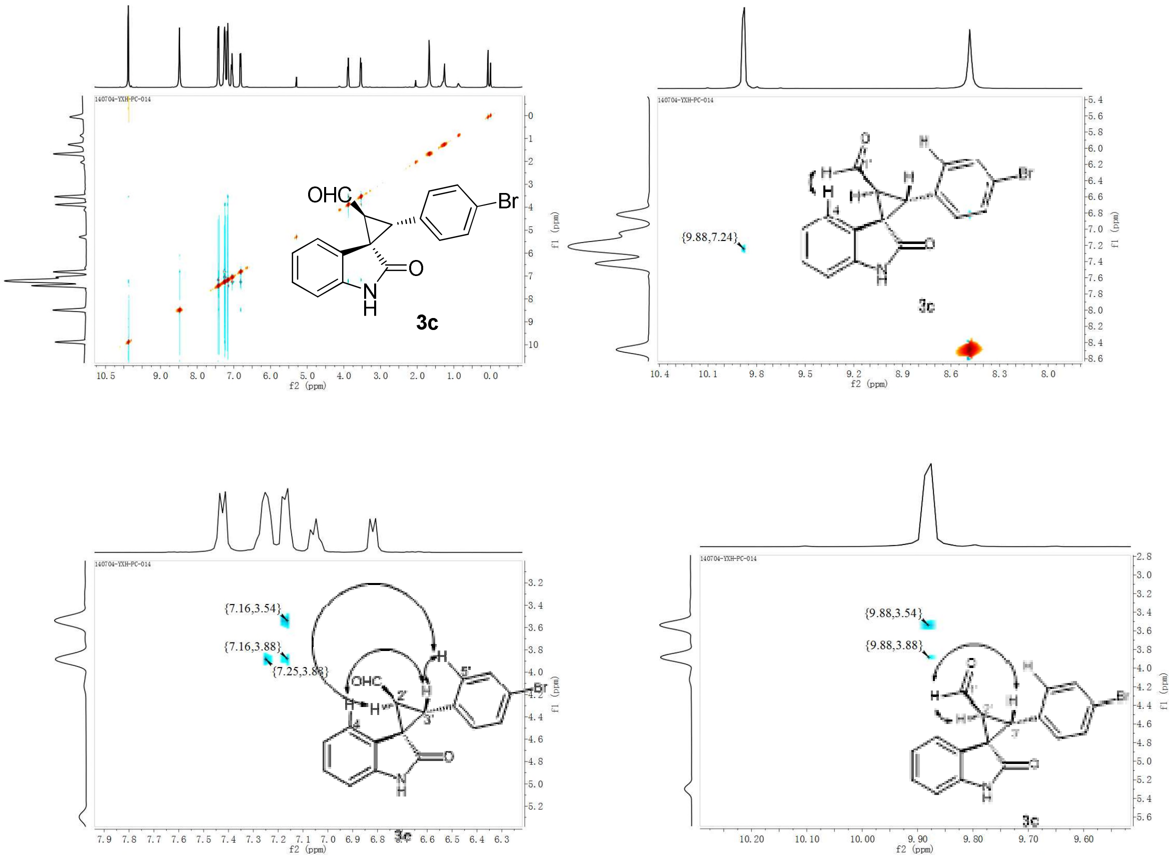

Supplementary Figure 1. NOE analysis for 3c

Relative stereochemistry of spirocyclopropyloxindole 3m was determined by NOE analysis. The fallowing results were obtained:

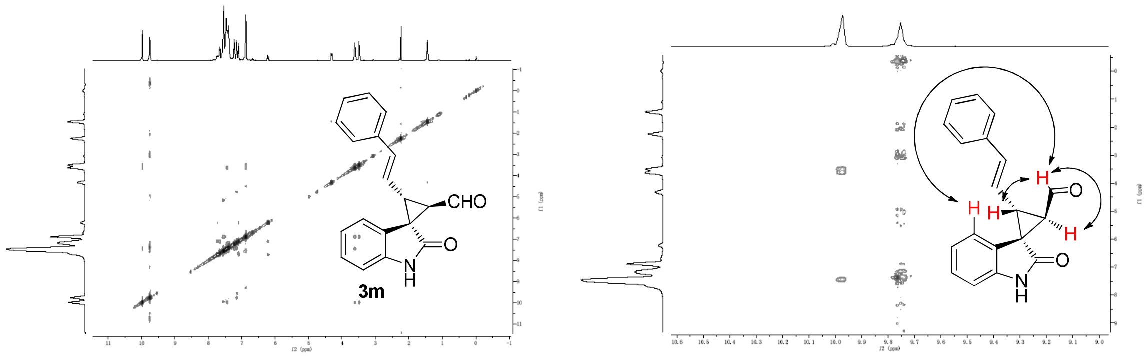

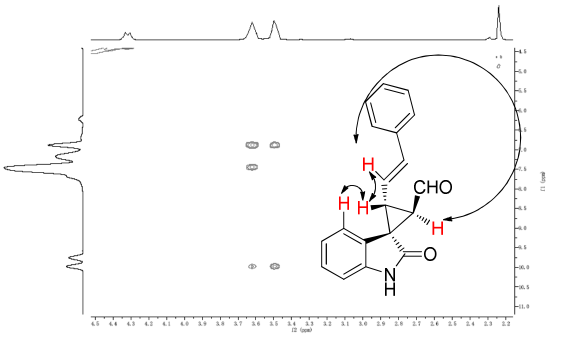

Supplementary Figure 2. NOE analysis for 3m

The dr value of 3a was obtained by analyzing the hydrogen spectrum splitting of the aldehyde hydrogens on the diastereomers. The fallowing results were obtained: dr$_{3a}$ =0.11:1.00 =10:90

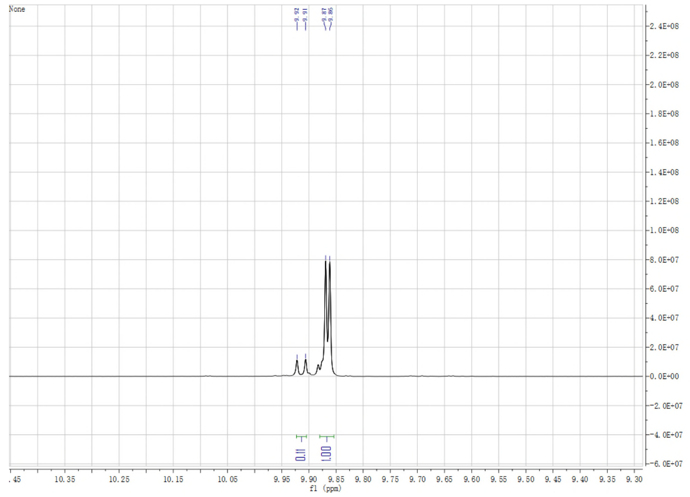

Supplementary Figure 3. hydrogen spectrum splitting analysis for 3a

2.4 NMR Spectra

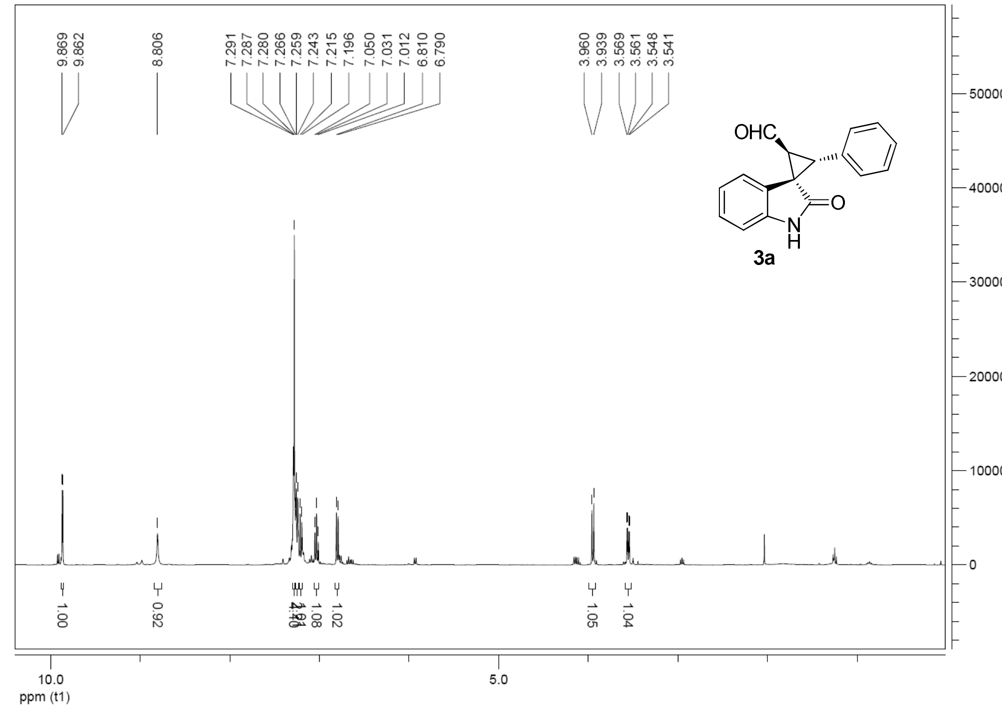

Supplementary Figure 4. ^{1}H NMR spectra of 3a

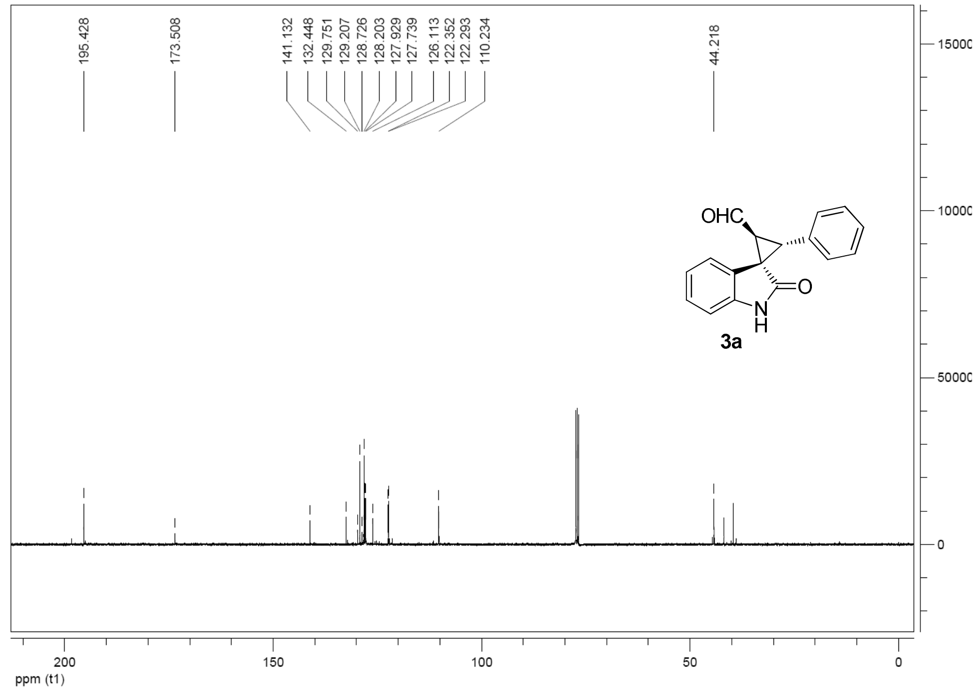

Supplementary Figure 5. ^{13}C NMR spectra of 3a

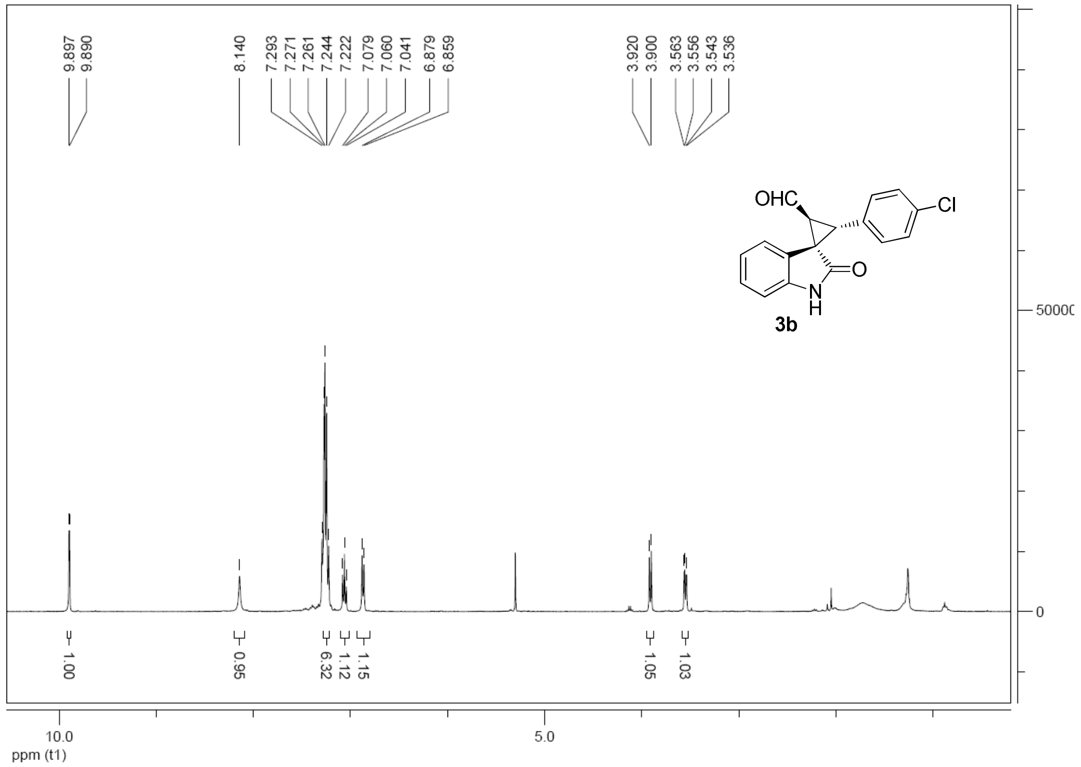

Supplementary Figure 6. ^{1}H NMR spectra of 3b

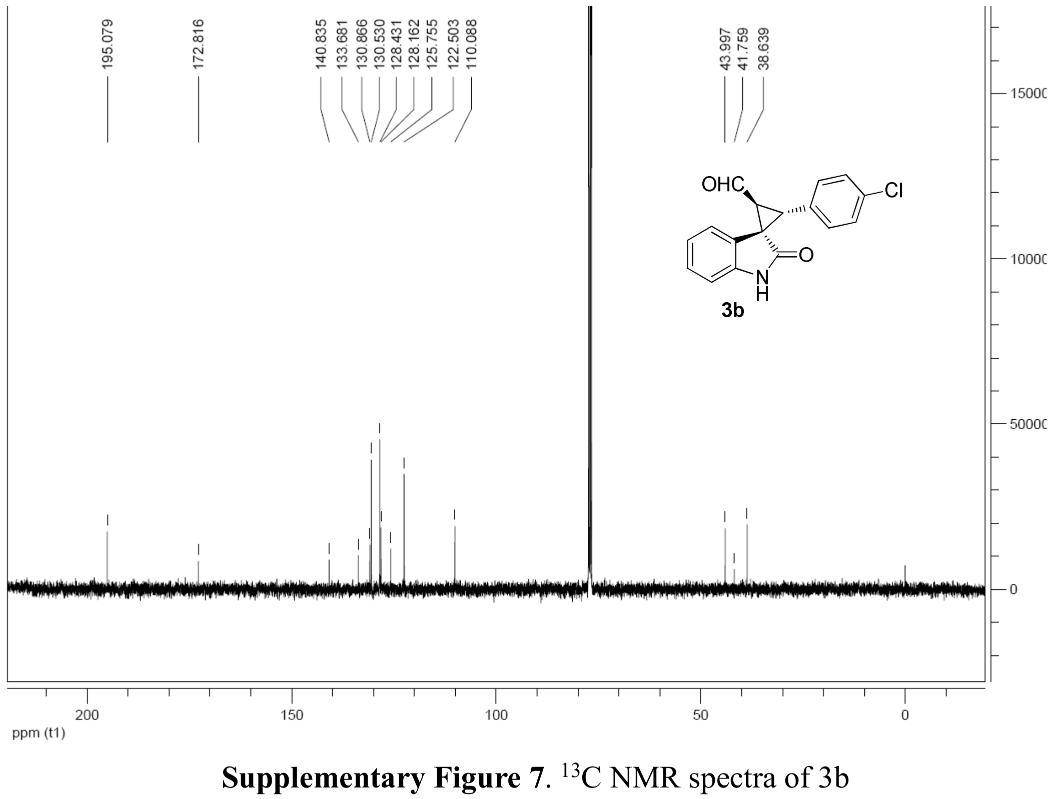

Supplementary Figure 7. ^{13}C NMR spectra of 3b

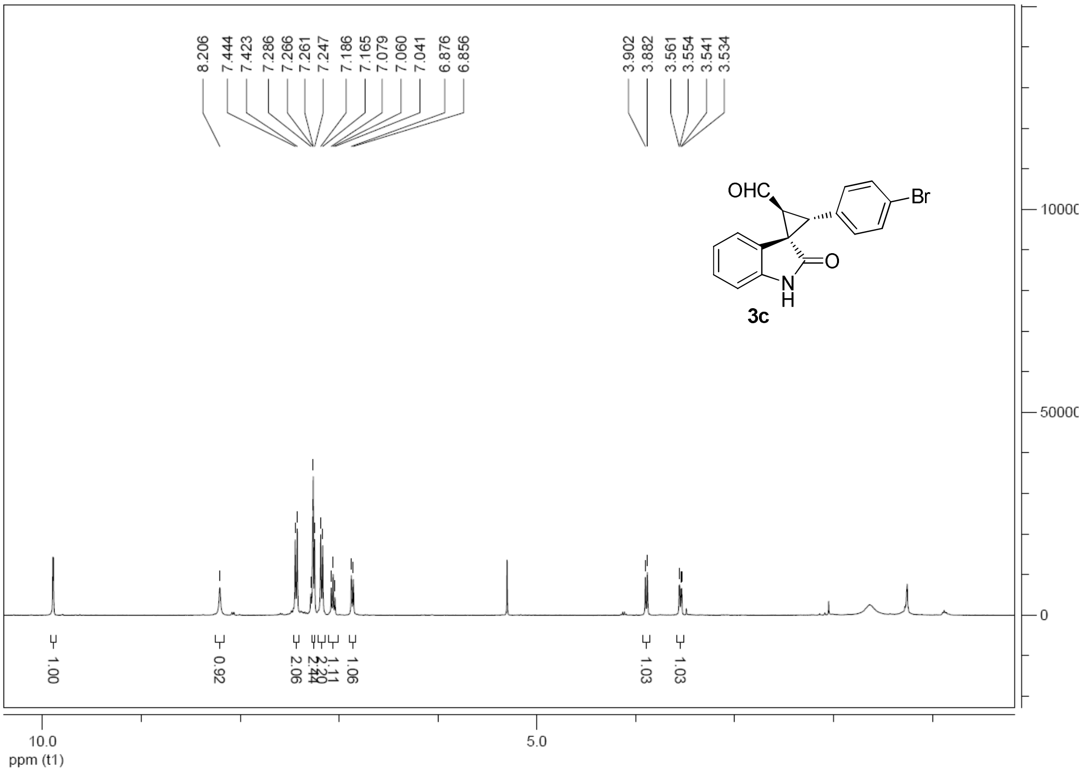

Supplementary Figure 8. ^{1}H NMR spectra of 3c

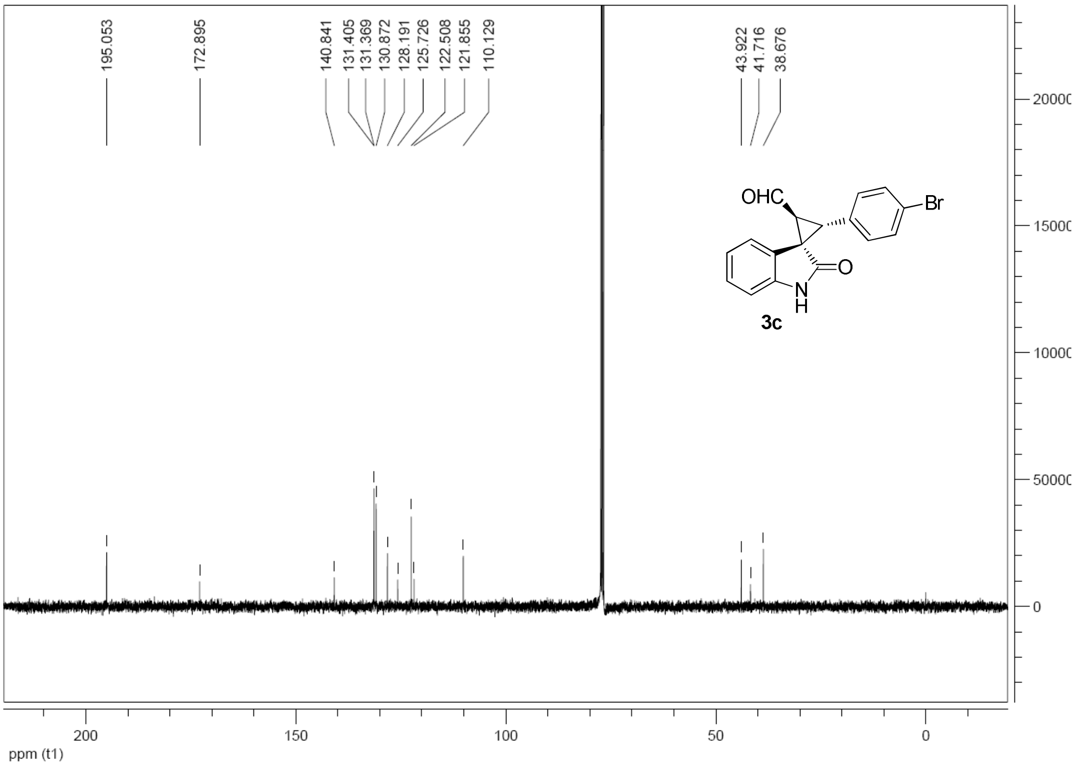

Supplementary Figure 9. ^{13}C NMR spectra of 3c

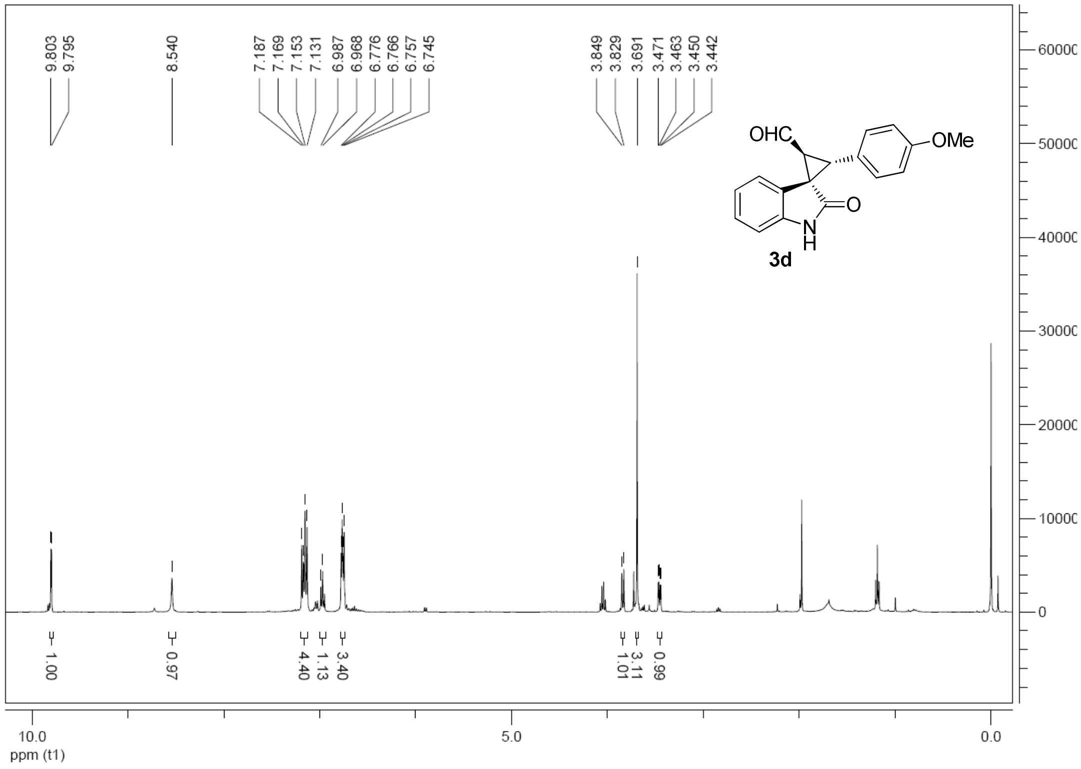

Supplementary Figure 10. ^{1}H NMR spectra of 3d

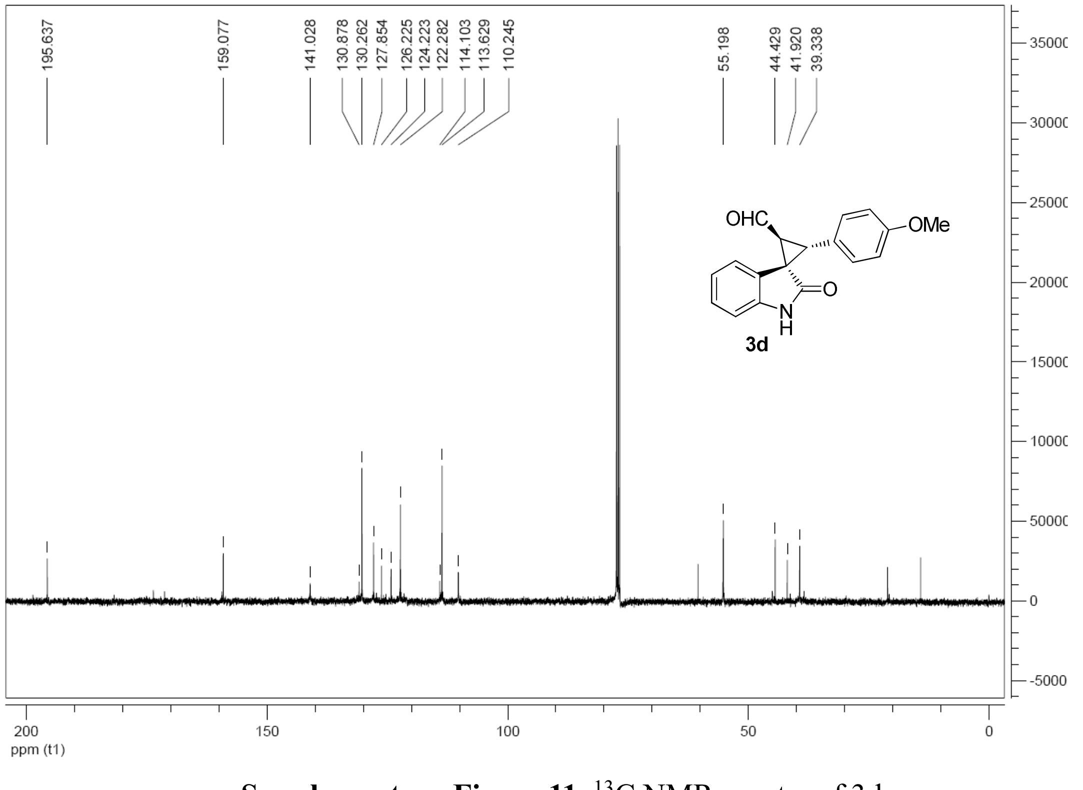

Supplementary Figure 11. ^{13}C NMR spectra of 3d

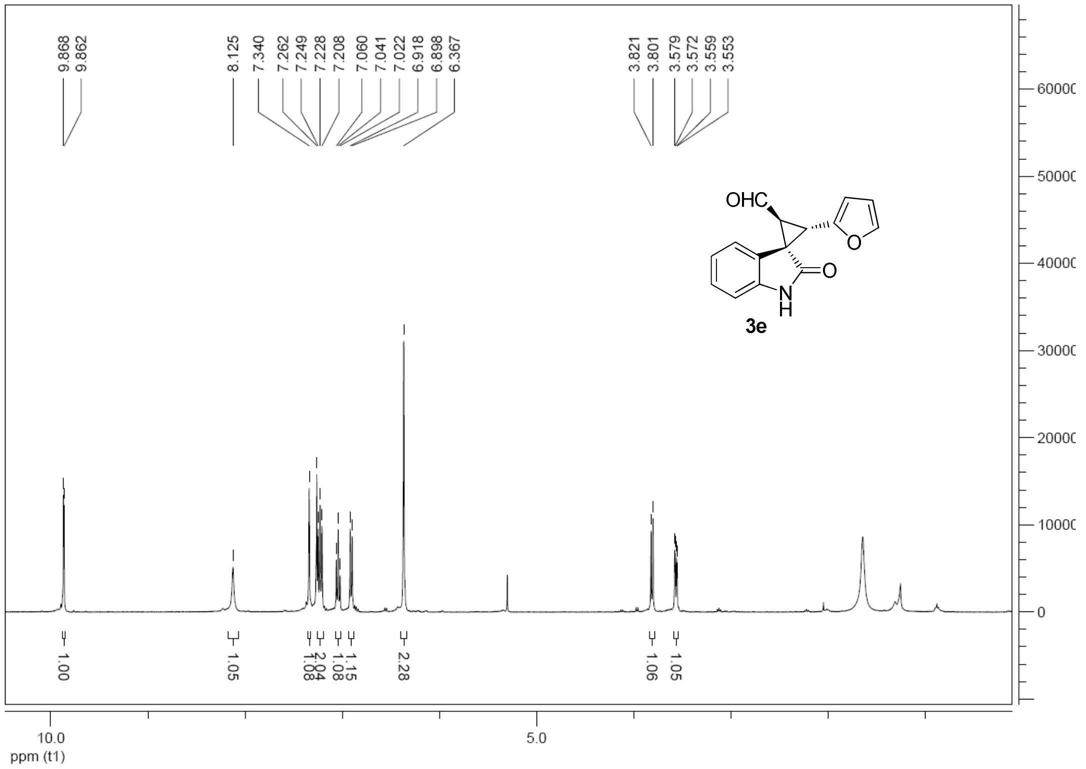

Supplementary Figure 12. ^{1}H NMR spectra of 3e

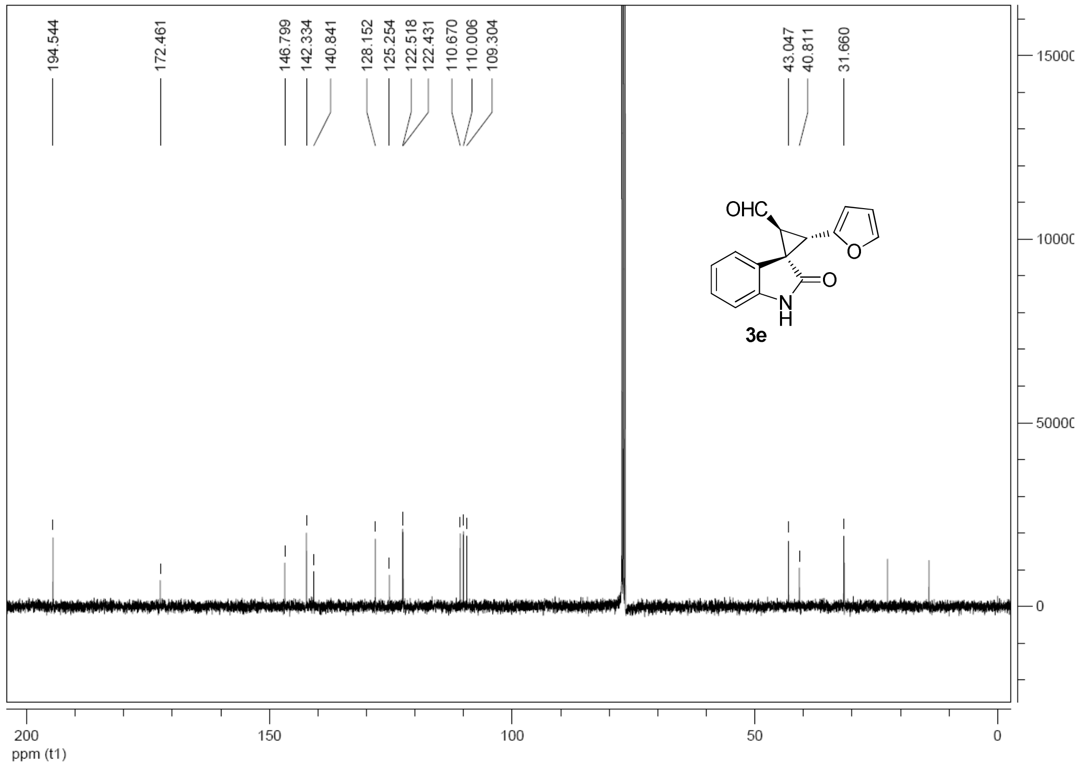

Supplementary Figure 13. ^{13}C NMR spectra of 3e

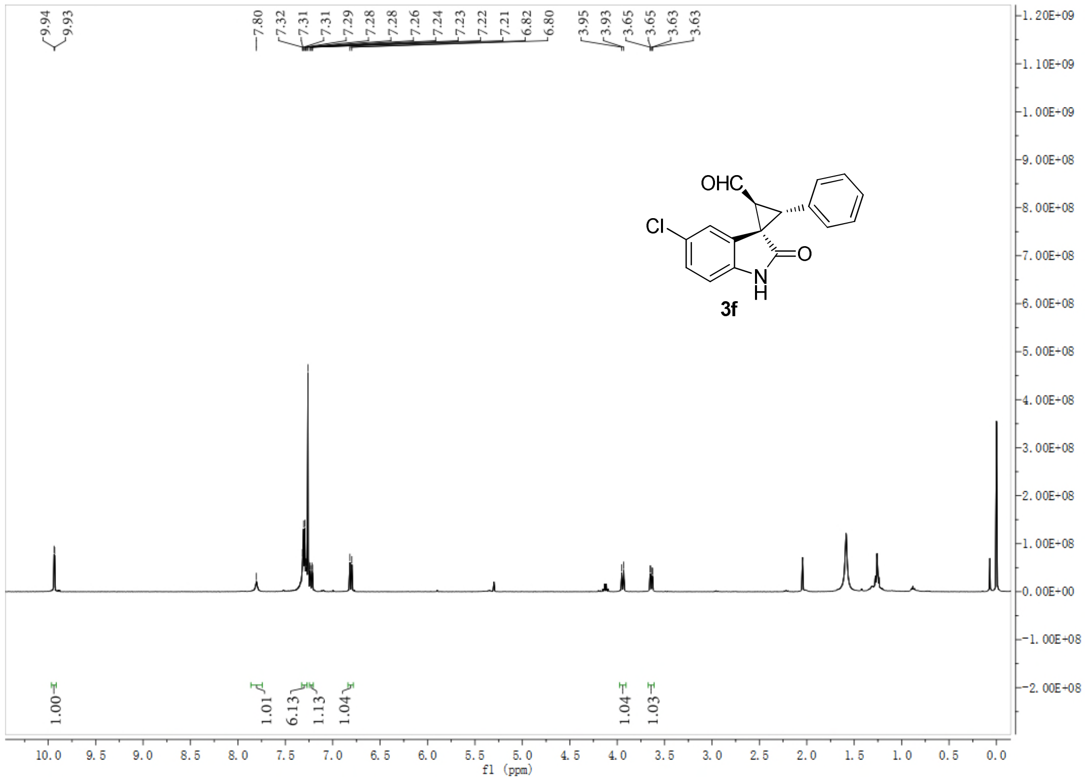

Supplementary Figure 14. ^{1}H NMR spectra of 3f

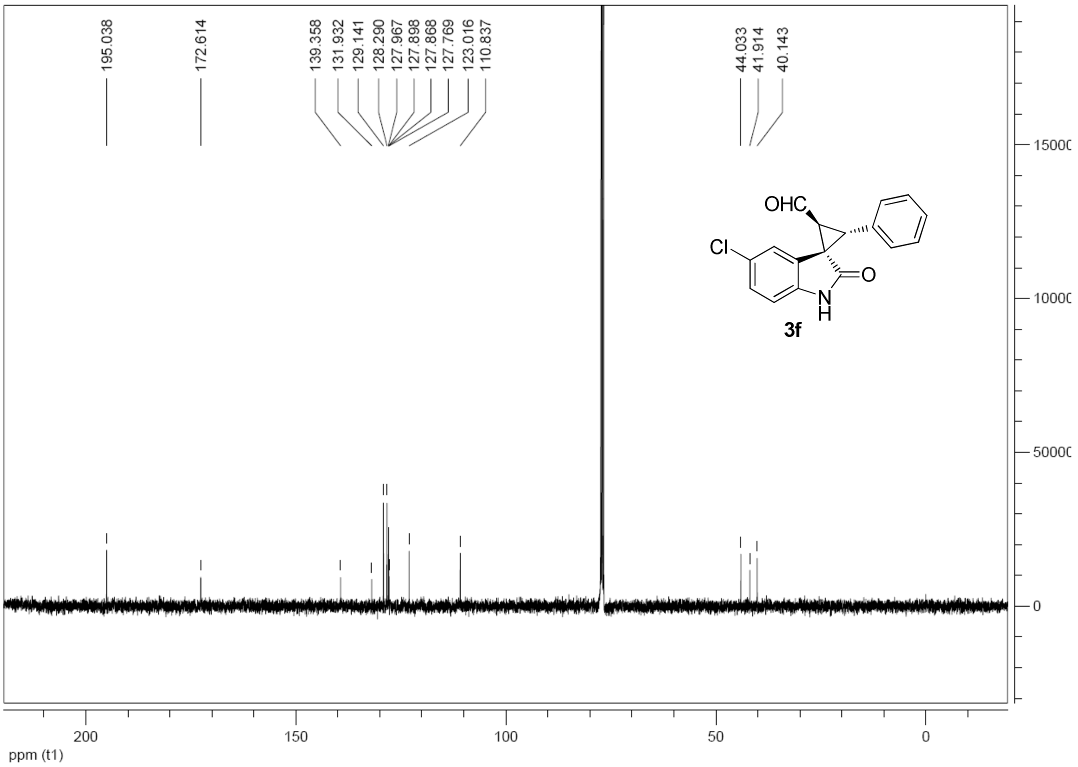

Supplementary Figure 15. ^{13}C NMR spectra of 3f

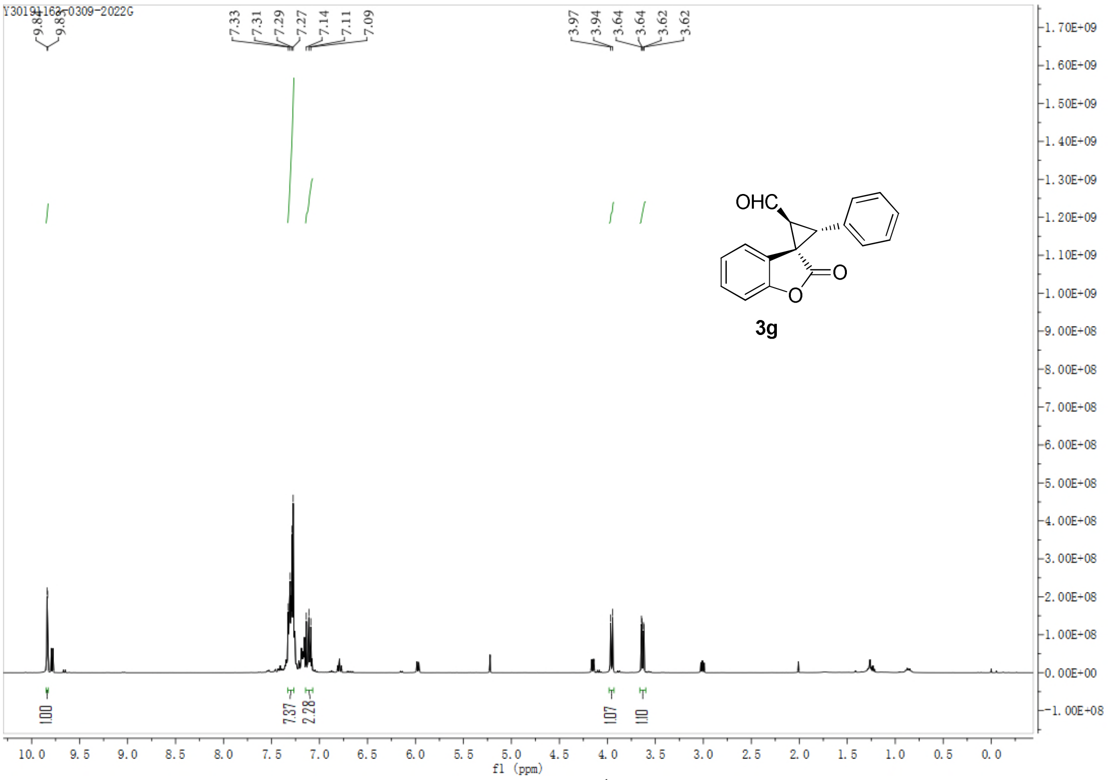

Supplementary Figure 16. ^{1}H NMR spectra of 3g

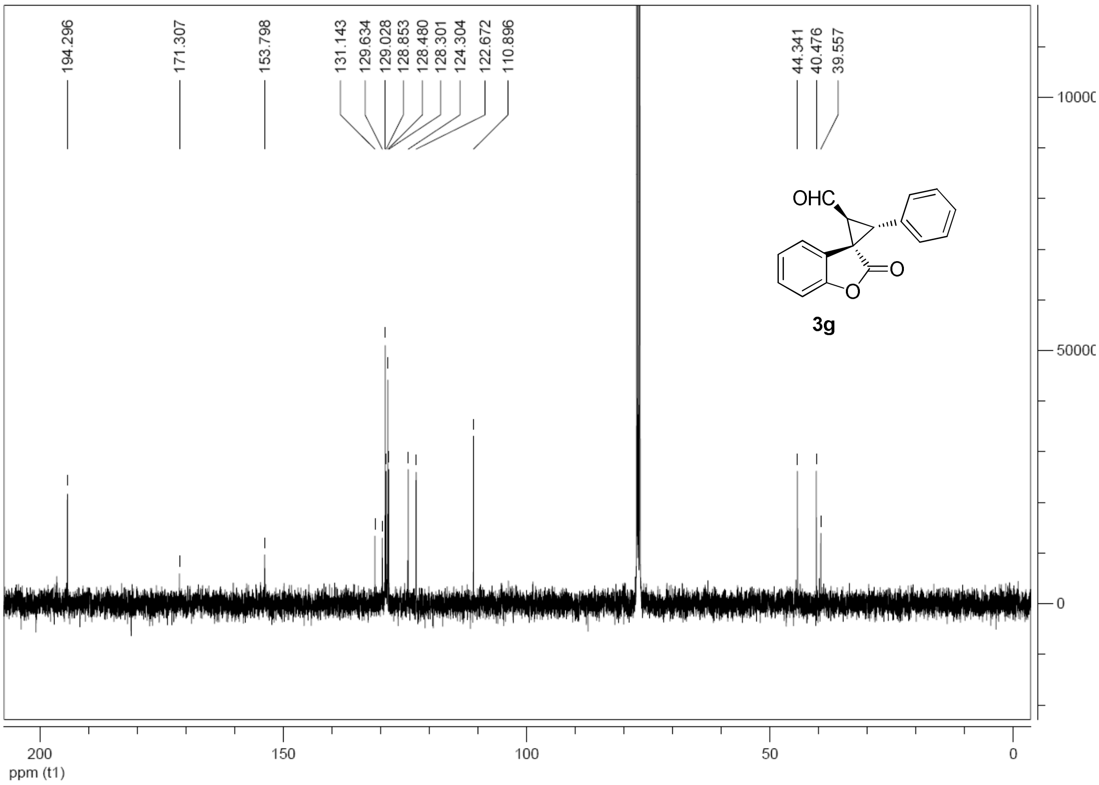

Supplementary Figure 17. ^{13}C NMR spectra of 3g

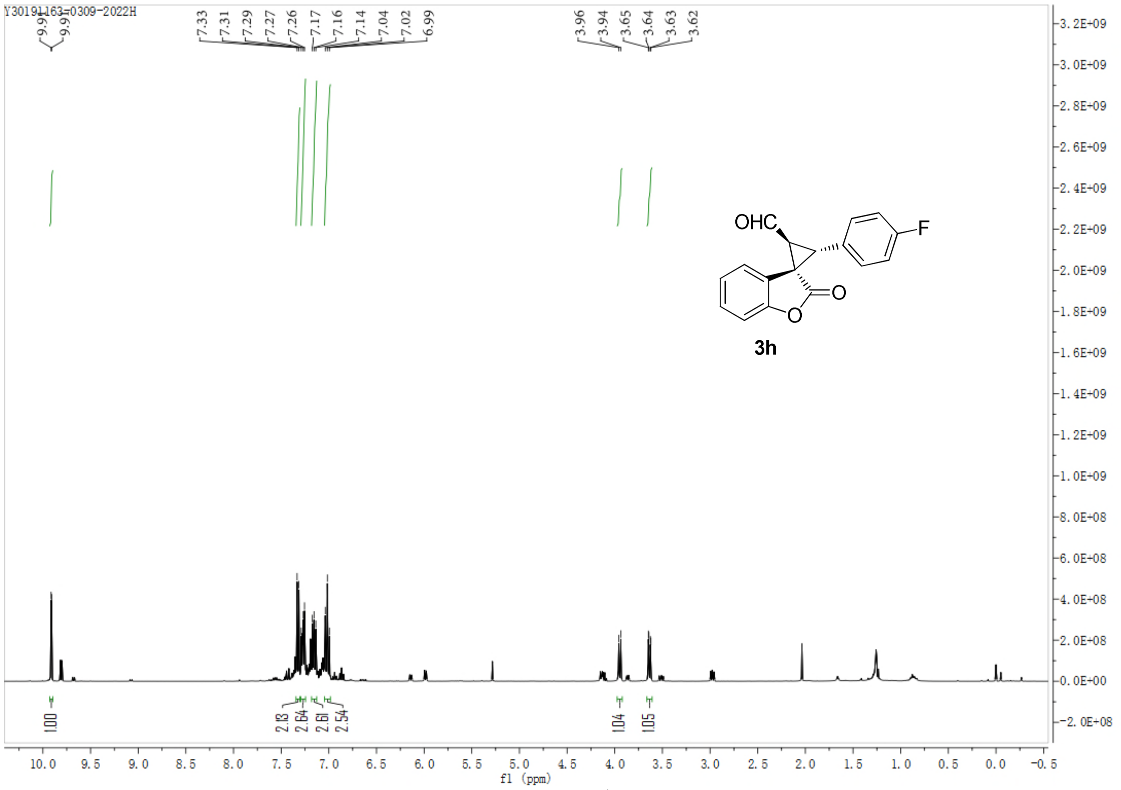

Supplementary Figure 18. ^{1}H NMR spectra of 3h

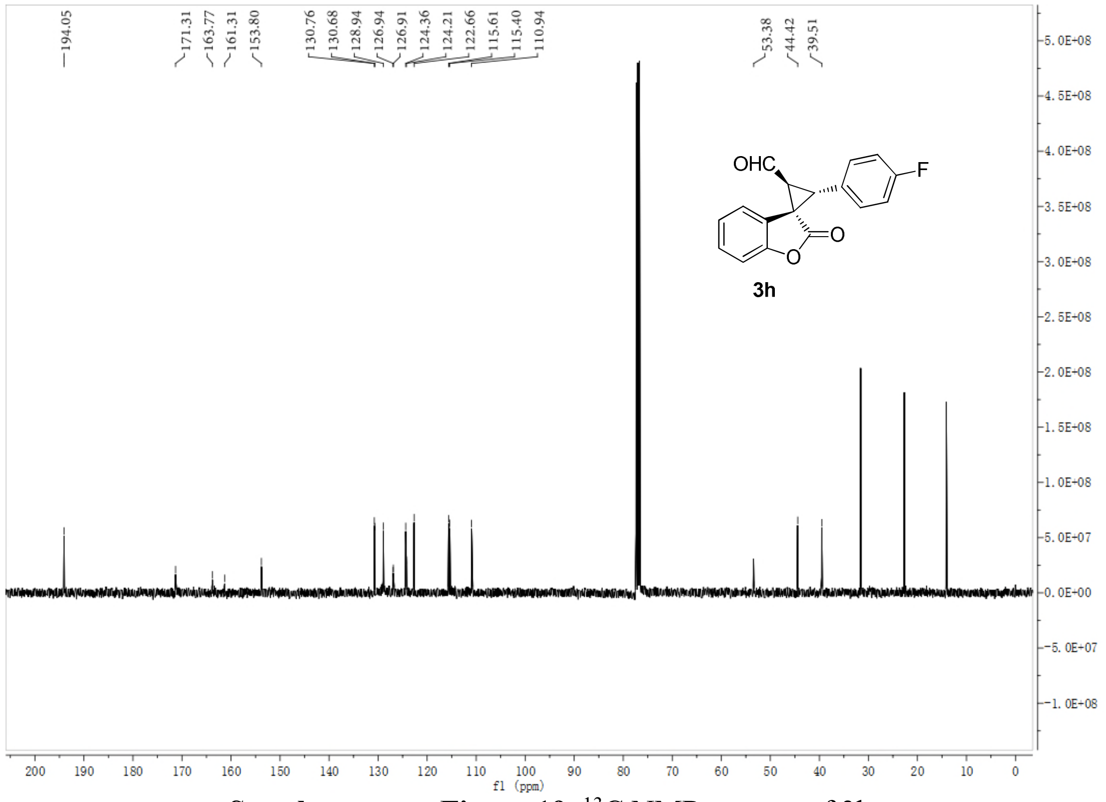

Supplementary Figure 19. ^{13}C NMR spectra of 3h

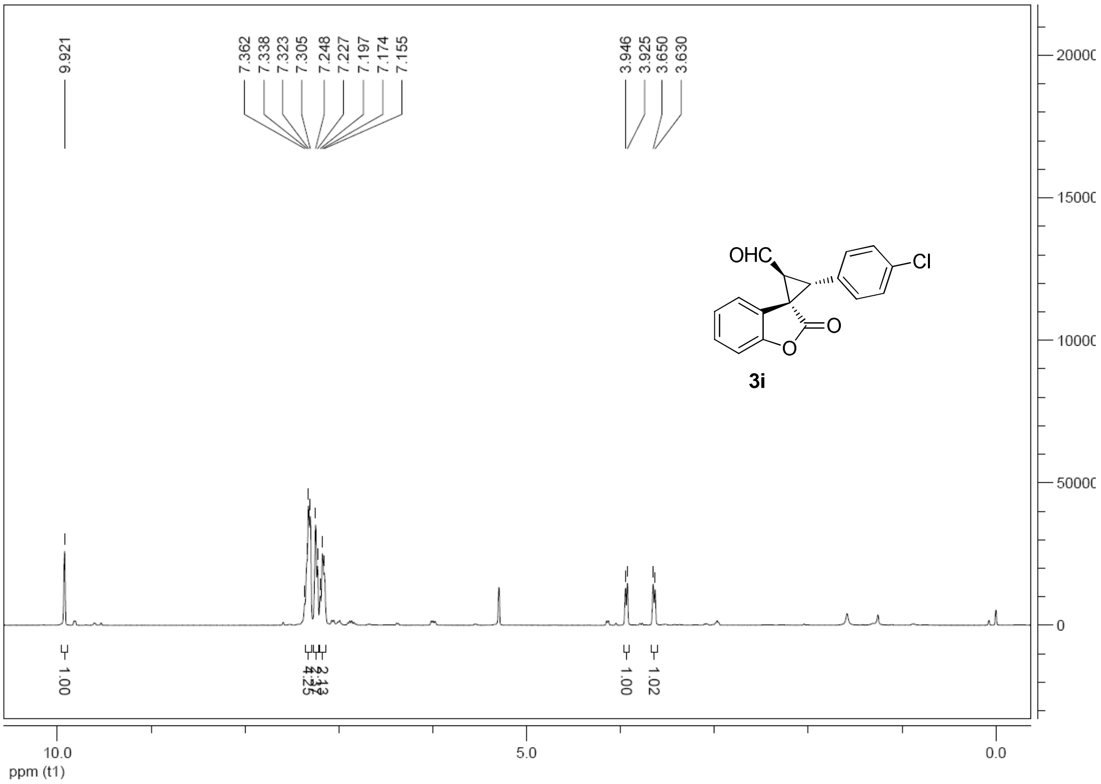

Supplementary Figure 20. ^{1}H NMR spectra of 3i

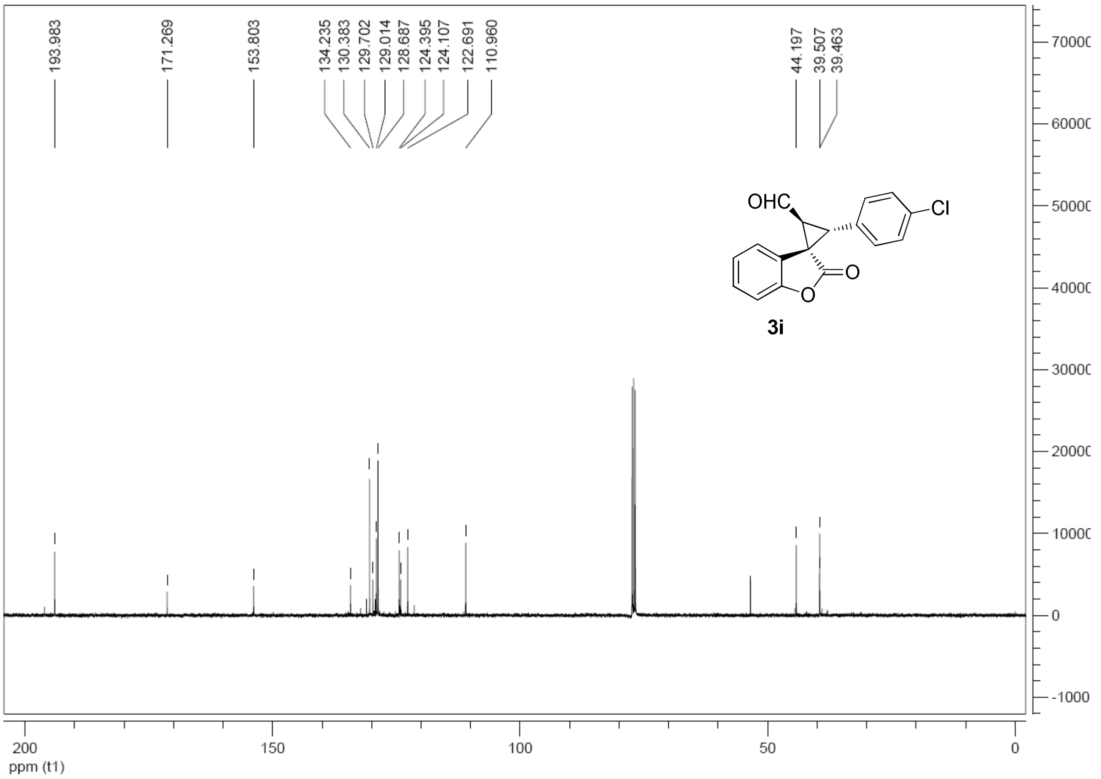

Supplementary Figure 21. ^{13}C NMR spectra of 3i

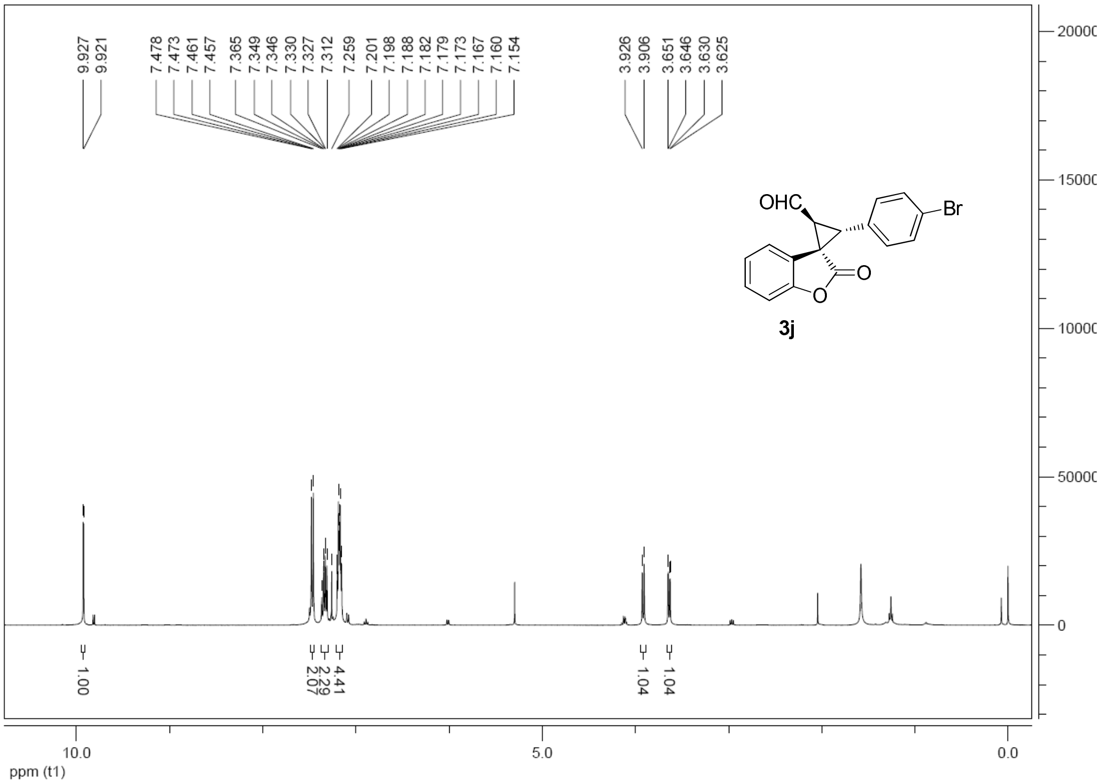

Supplementary Figure 22. ^{1}H NMR spectra of 3j

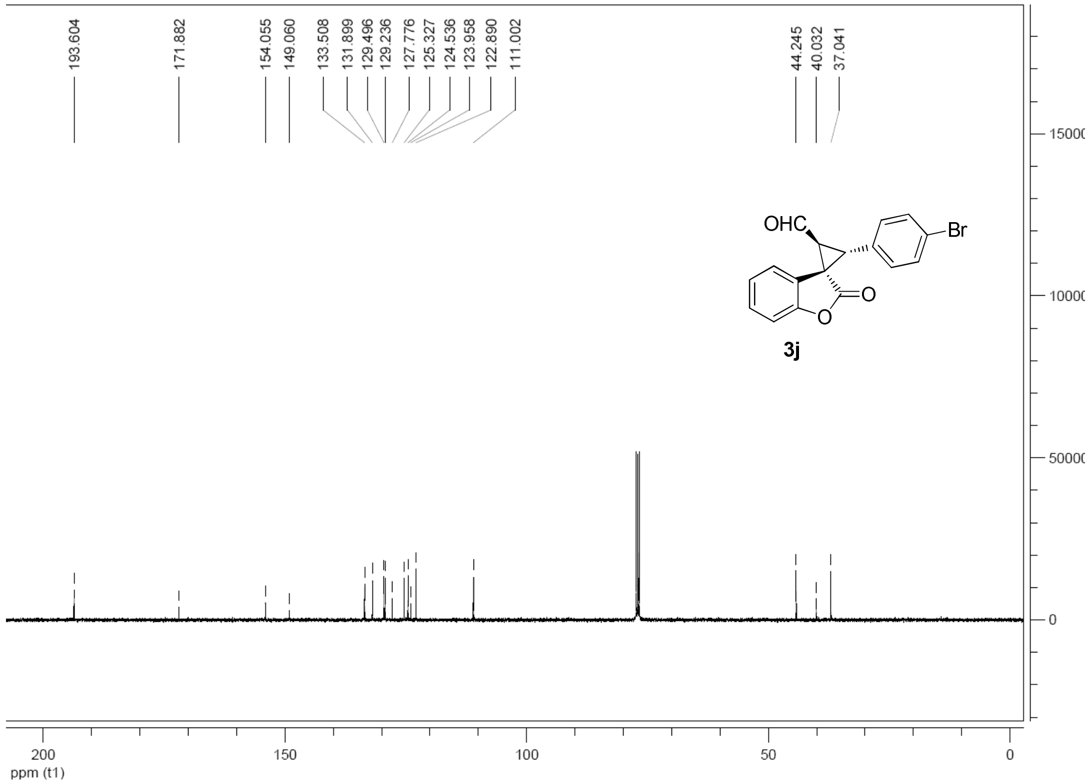

Supplementary Figure 23. ^{13}C NMR spectra of 3j

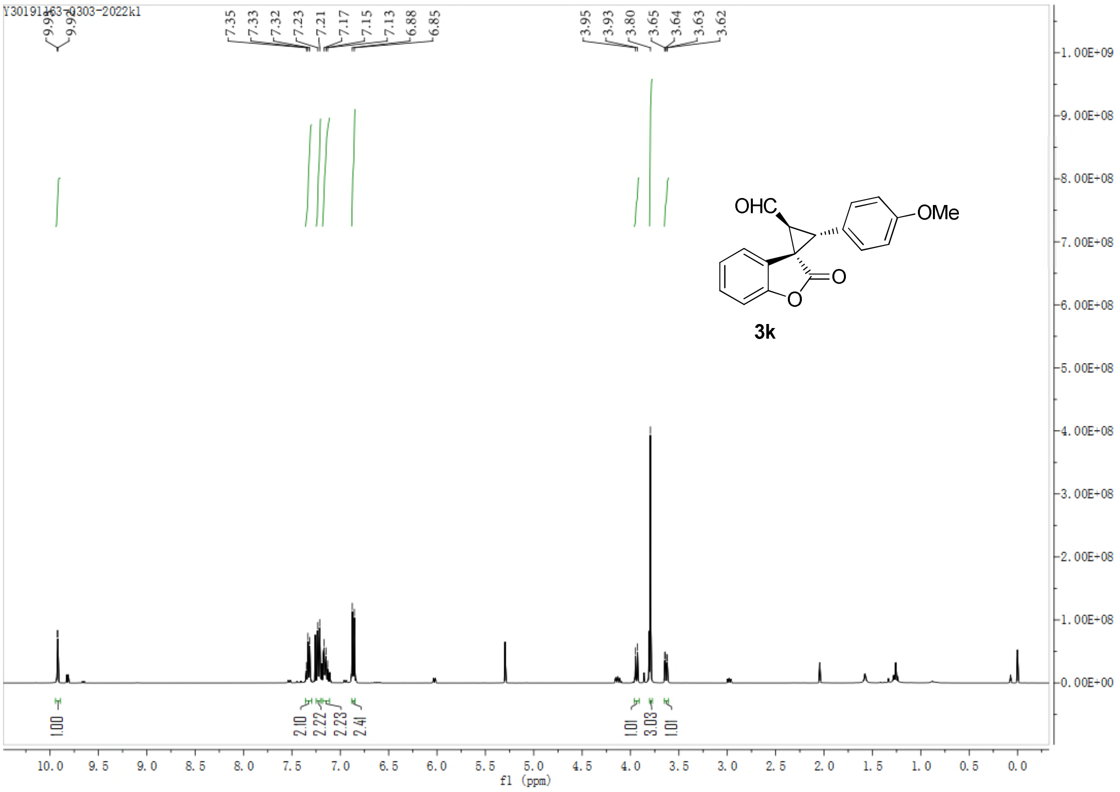

Supplementary Figure 24. ^{1}H NMR spectra of 3k

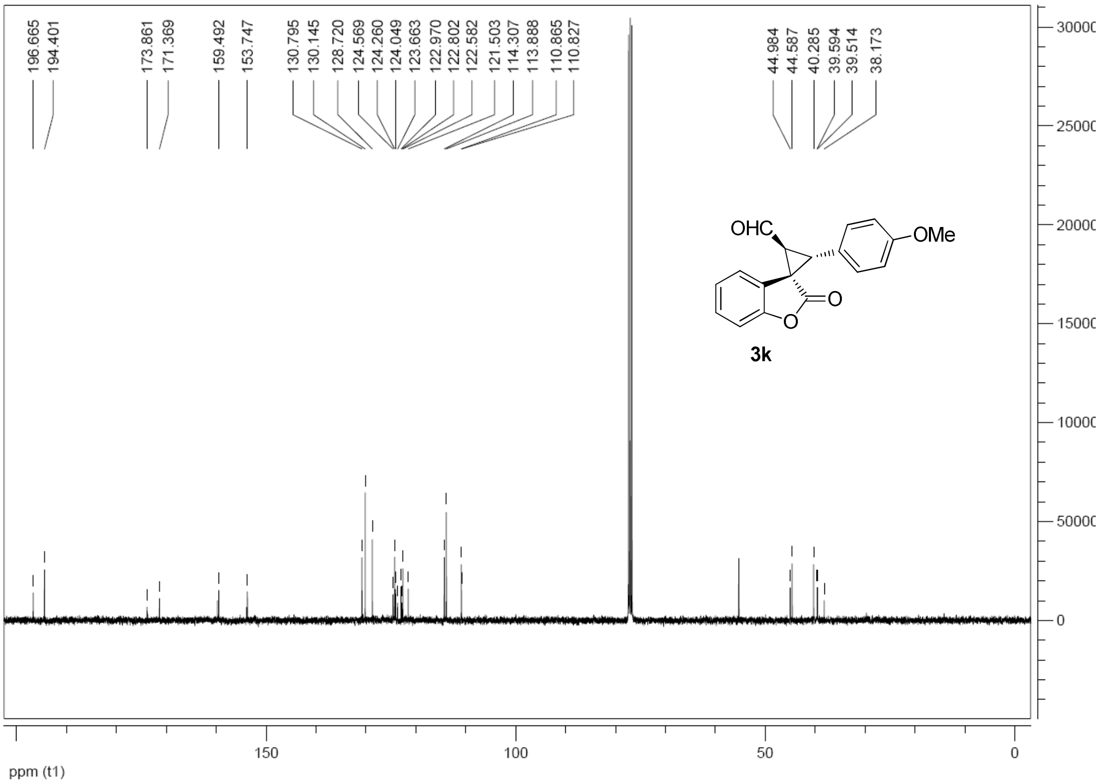

Supplementary Figure 25. ^{13}C NMR spectra of 3k

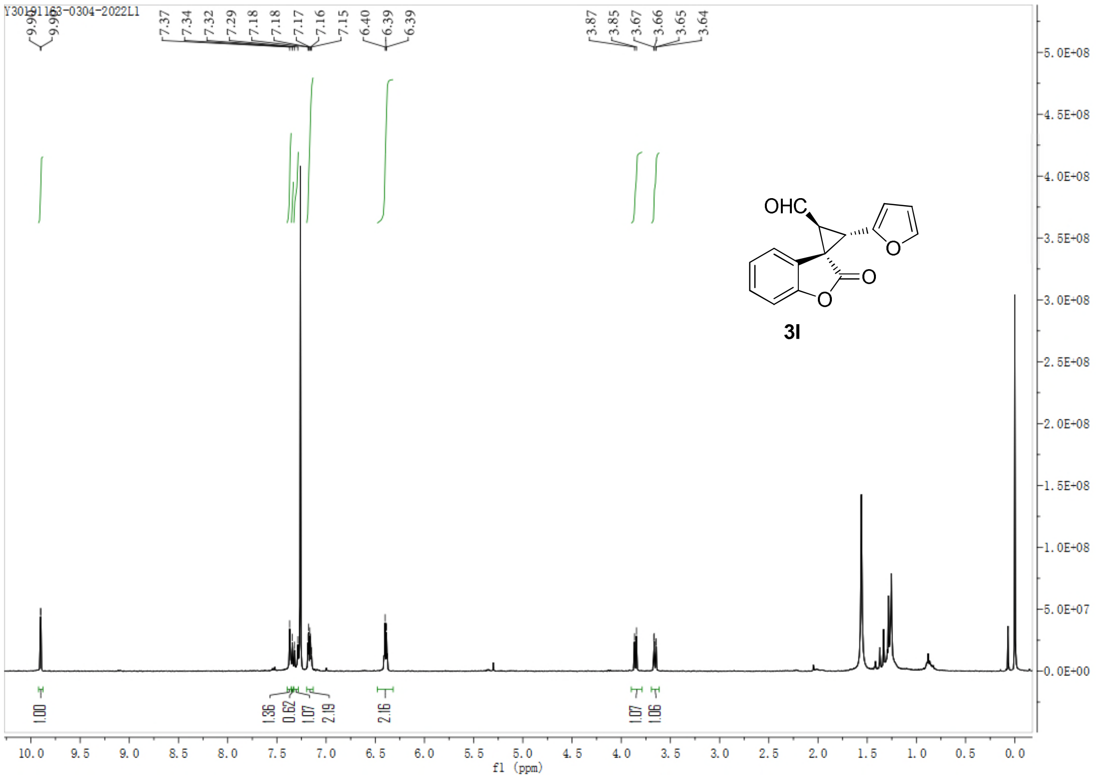

Supplementary Figure 26. ^{1}H NMR spectra of 3l

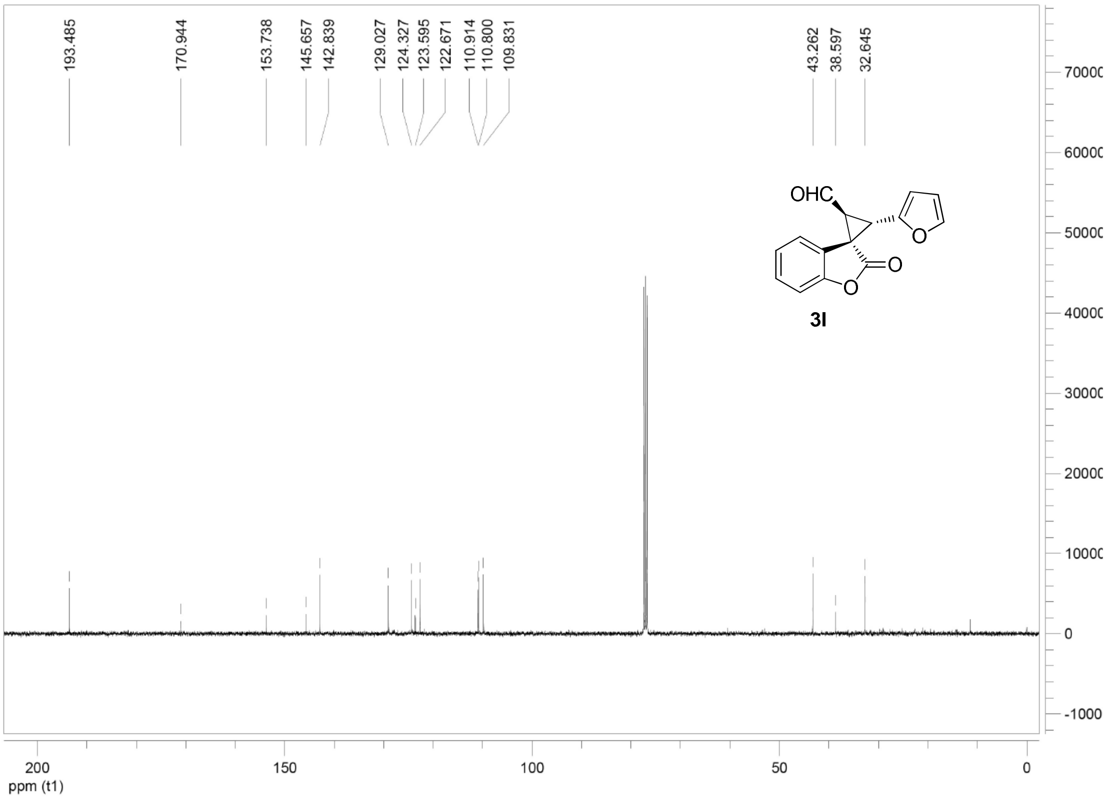

Supplementary Figure 27. ^{13}C NMR spectra of 3l

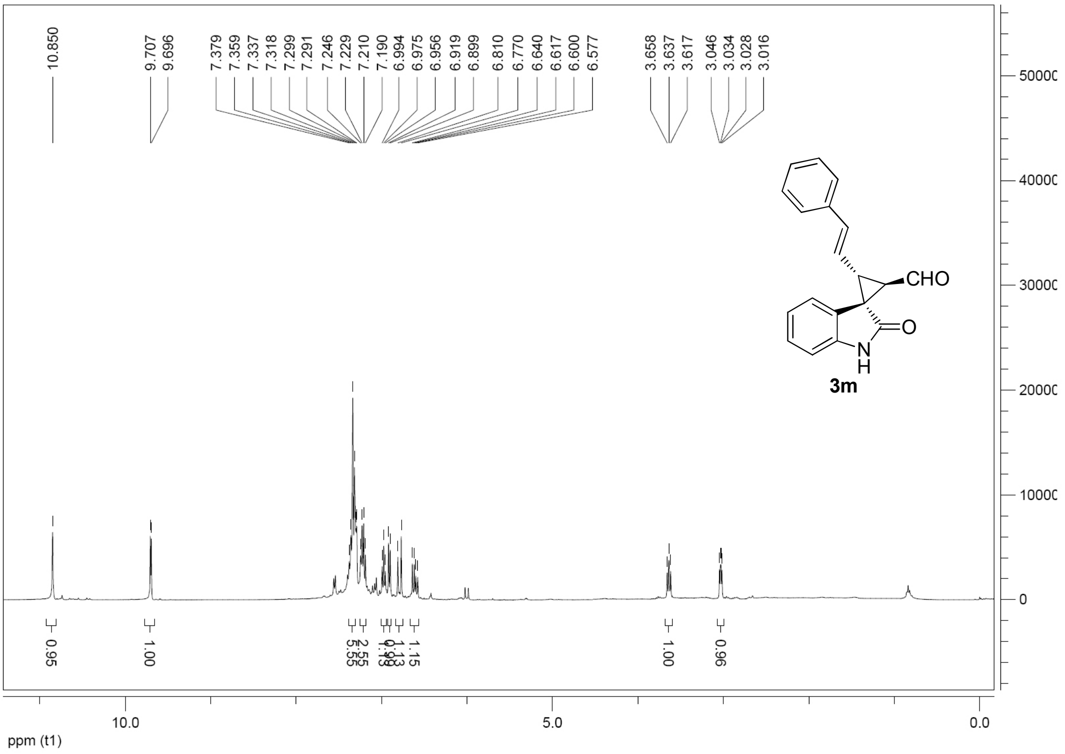

Supplementary Figure 28. ^{1}H NMR spectra of 3m

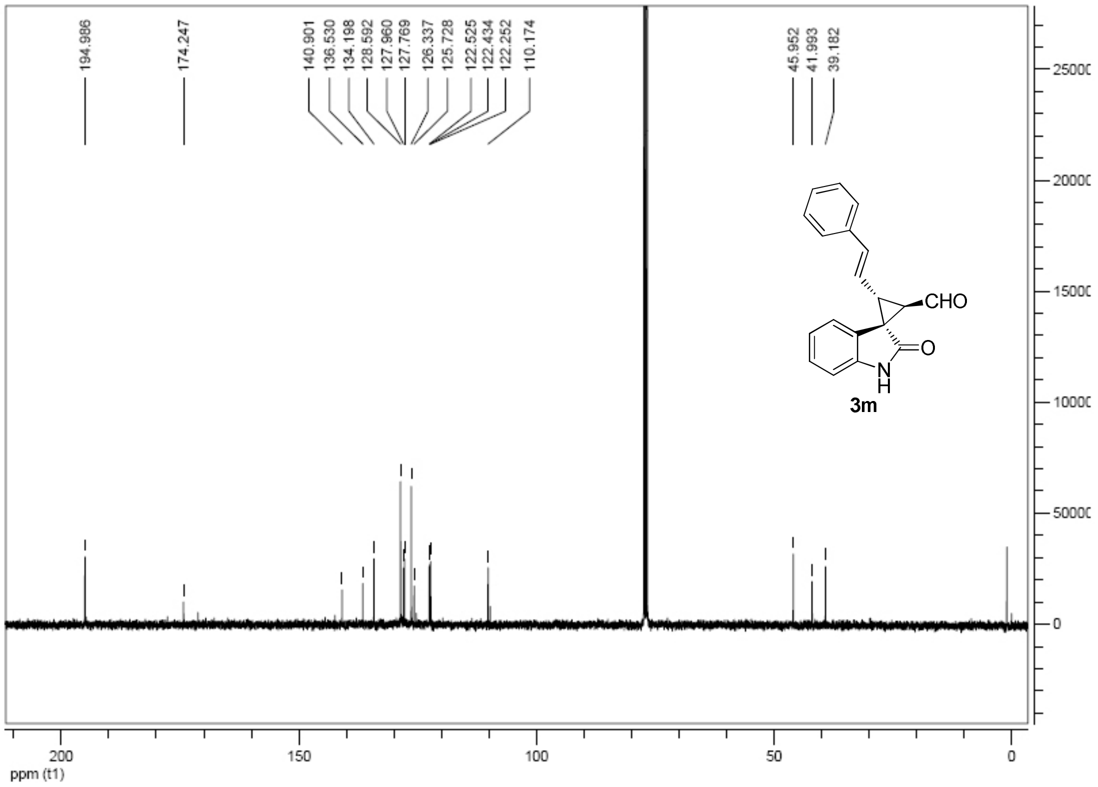

Supplementary Figure 29. ^{13}C NMR spectra of 3m

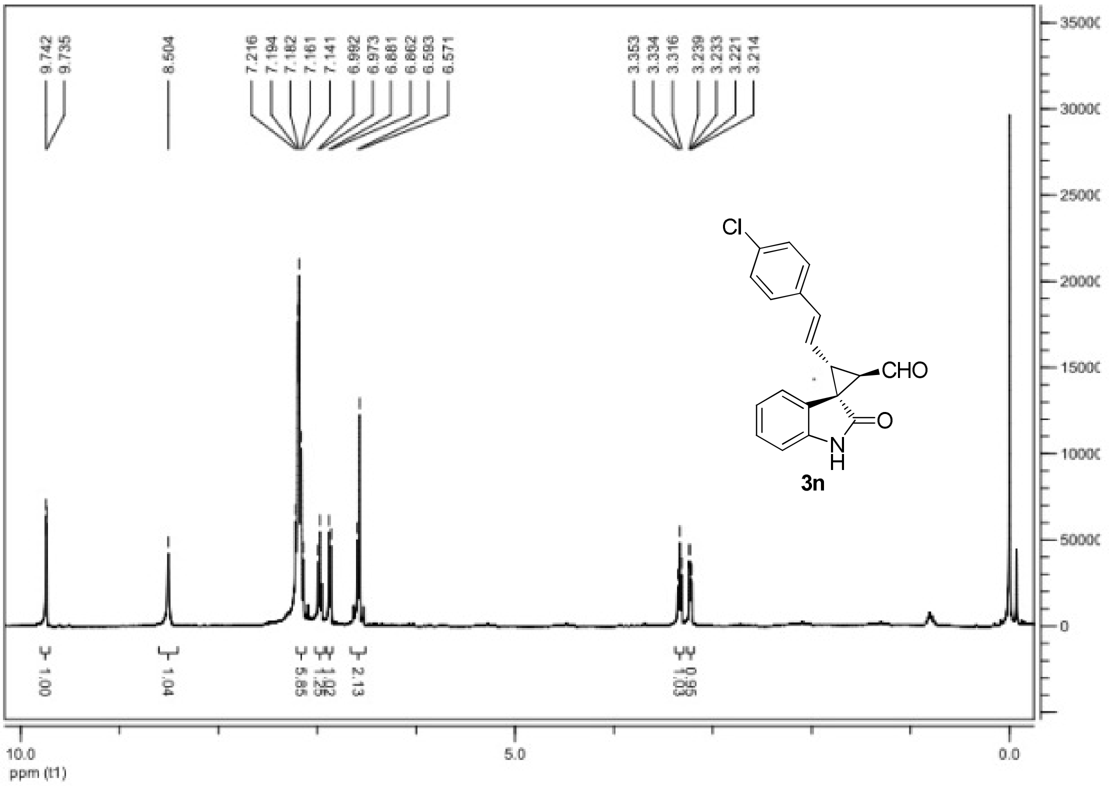

Supplementary Figure 30. ^{1}H NMR spectra of 3n

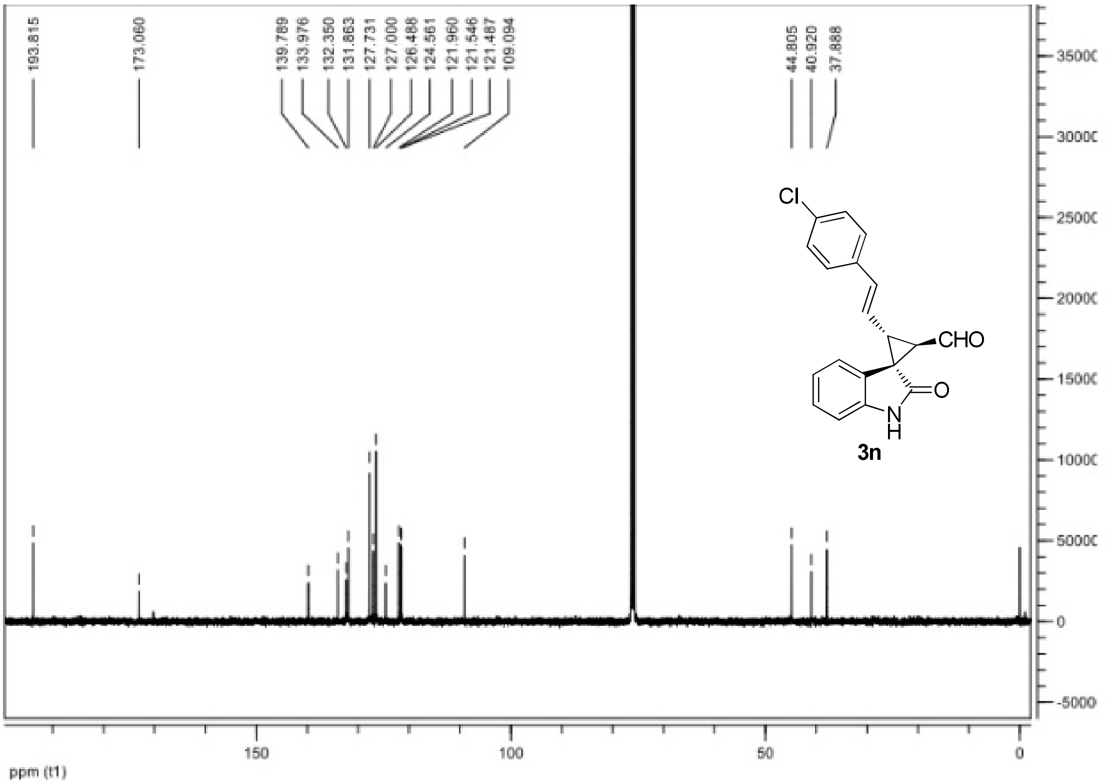

Supplementary Figure 31. ^{13}C NMR spectra of 3n

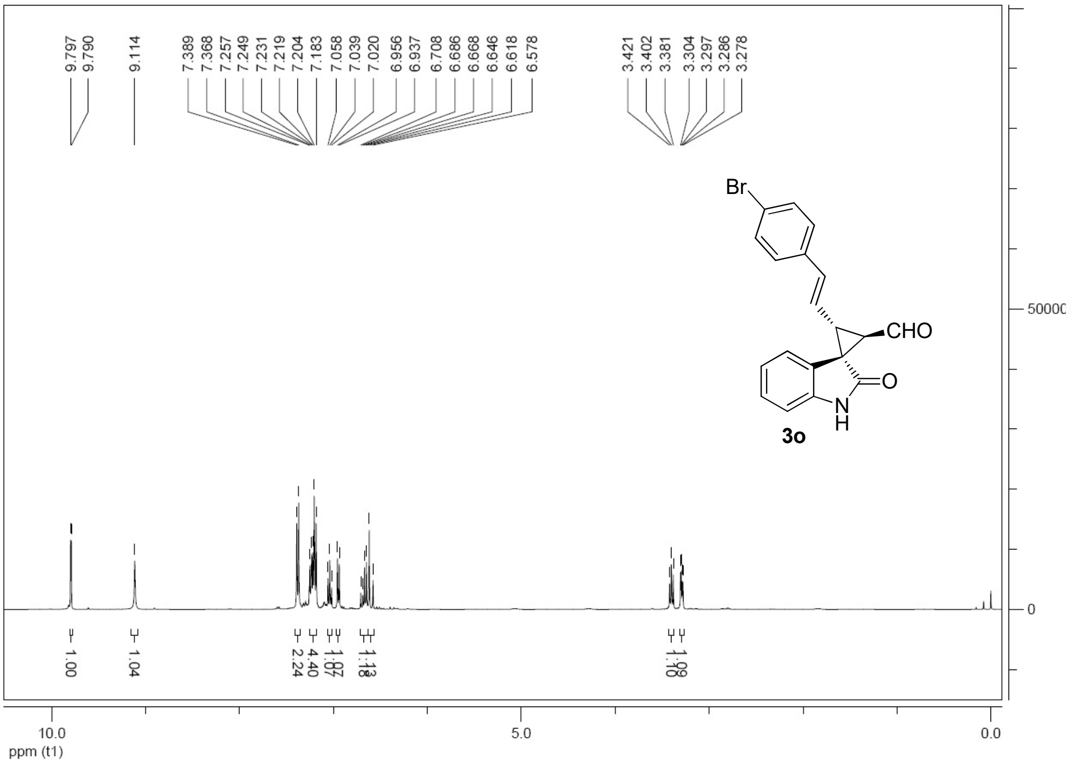

Supplementary Figure 32. ^{1}H NMR spectra of 3o

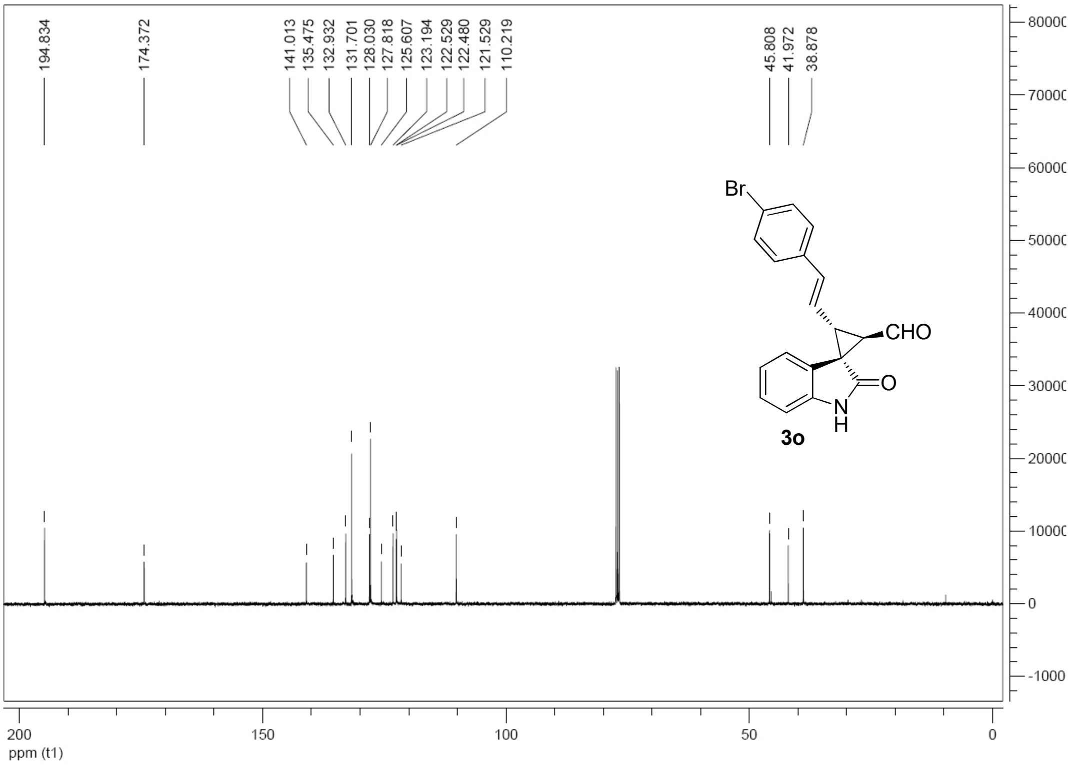

Supplementary Figure 33. ^{13}C NMR spectra of 3o

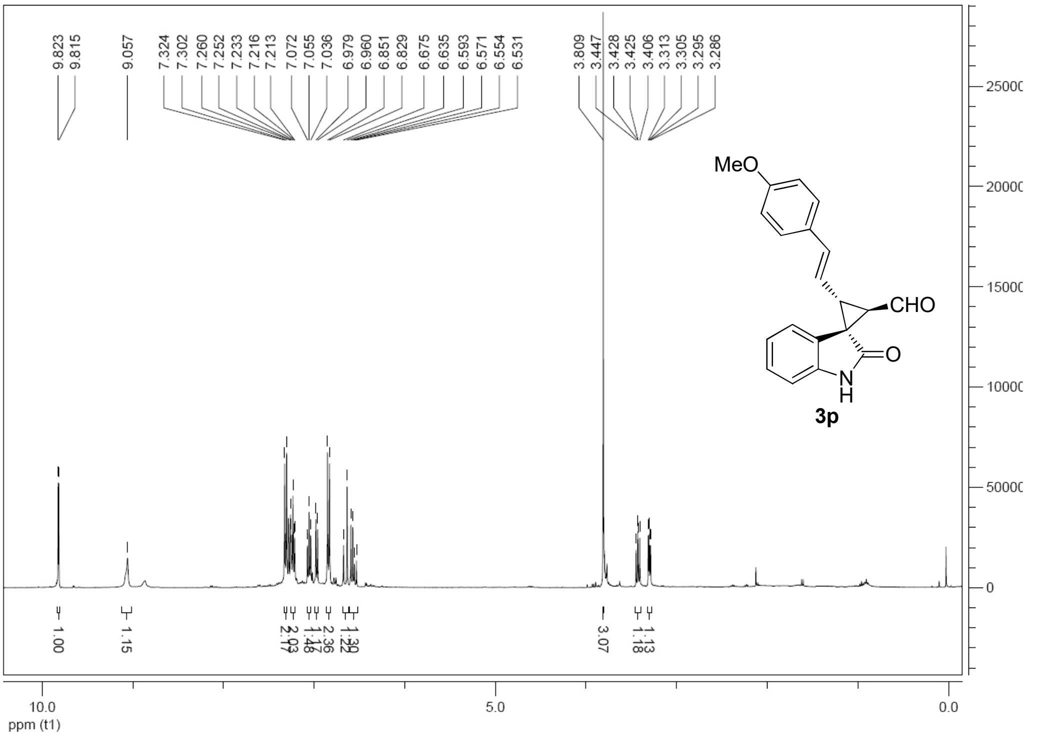

Supplementary Figure 34. ^{1}H NMR spectra of 3p

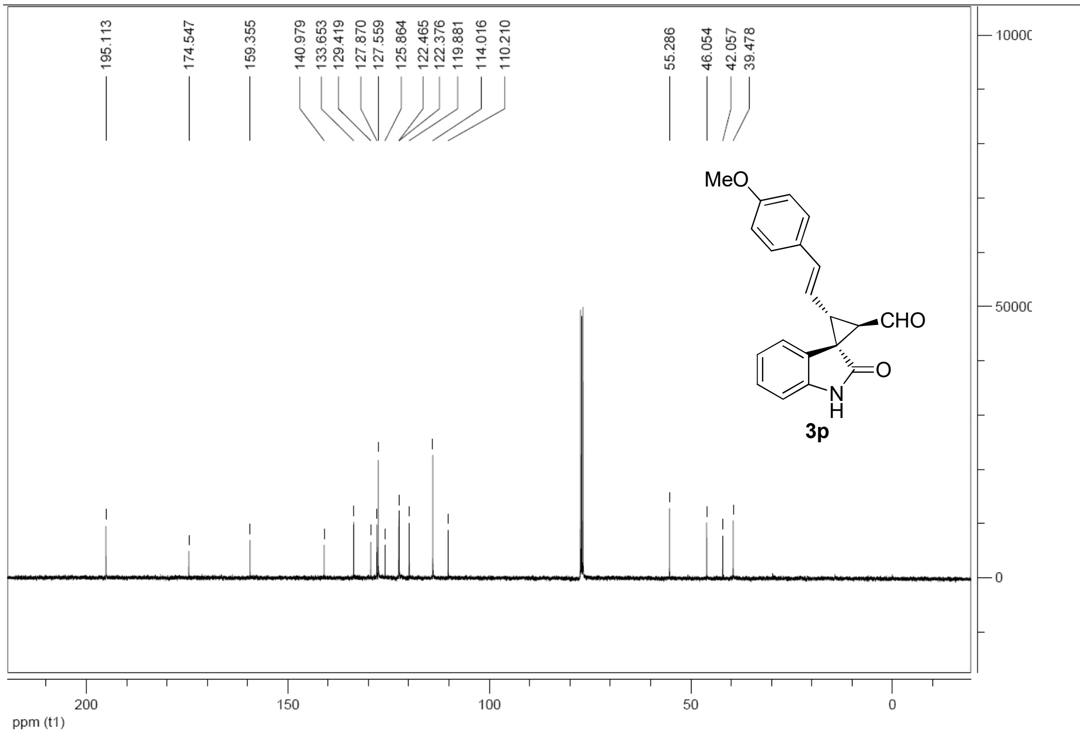

Supplementary Figure 35. ^{13}C NMR spectra of 3p

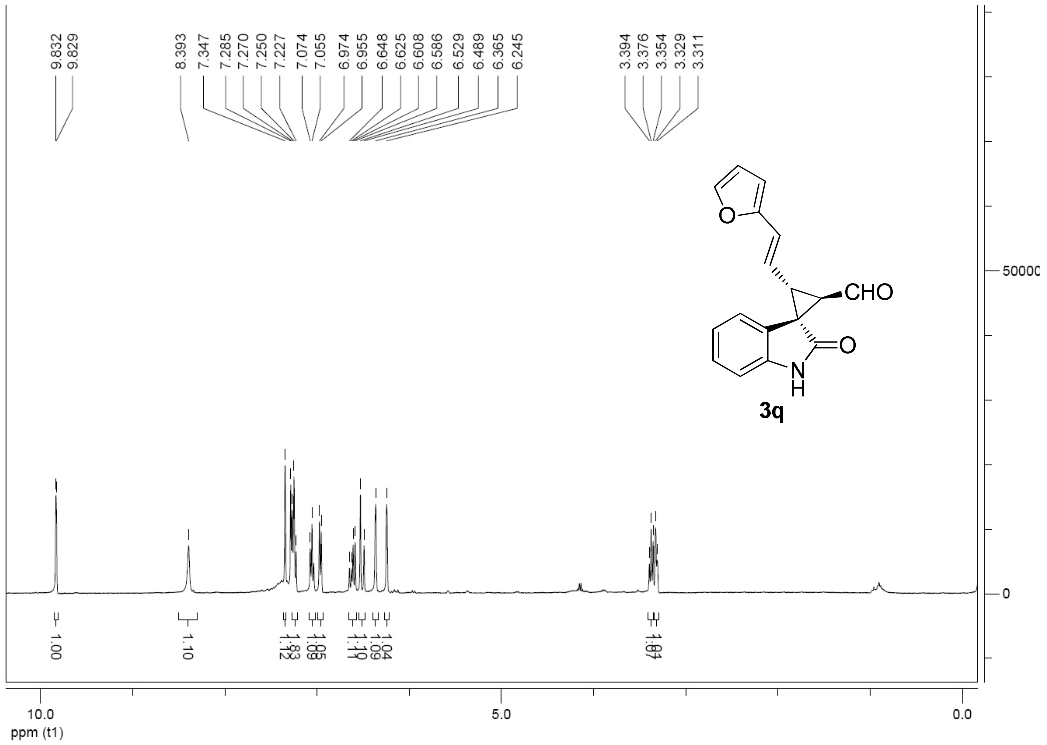

Supplementary Figure 36. ^{1}H NMR spectra of 3q

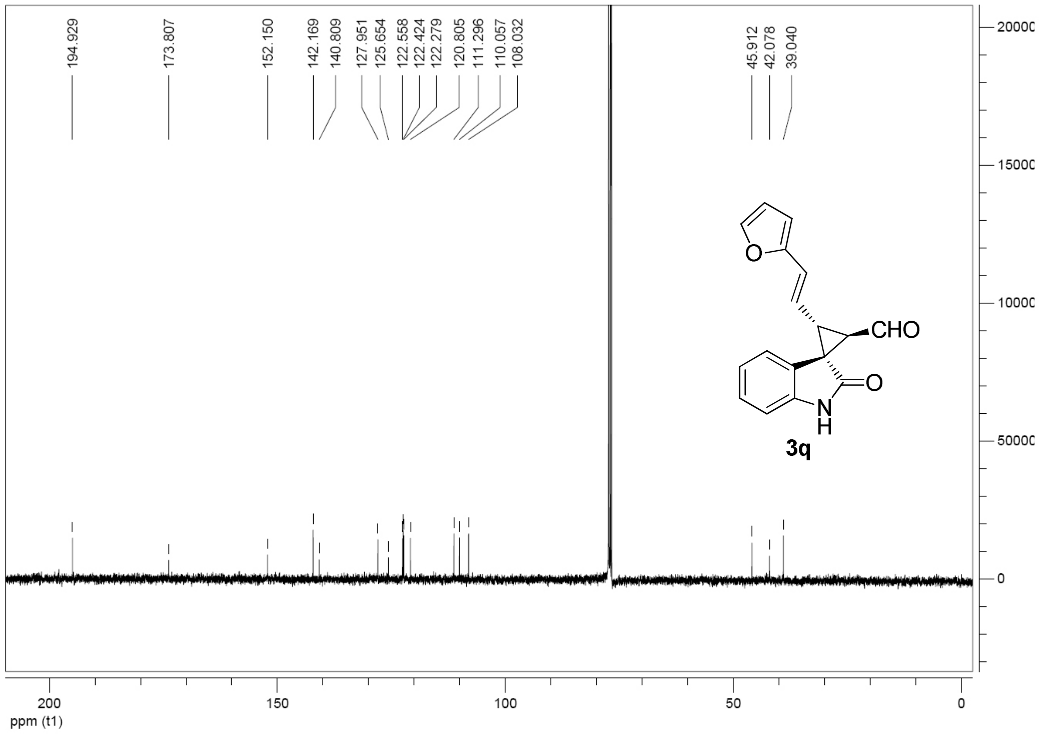

Supplementary Figure 37. ^{13}C NMR spectra of 3q

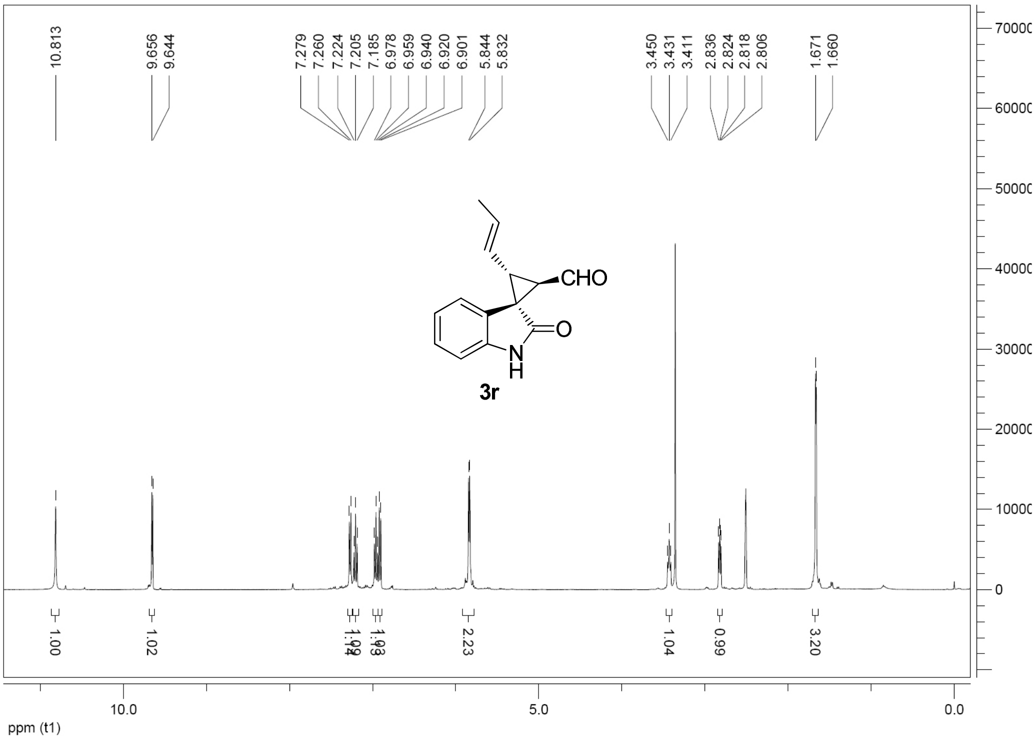

Supplementary Figure 38. ^{1}H NMR spectra of 3r

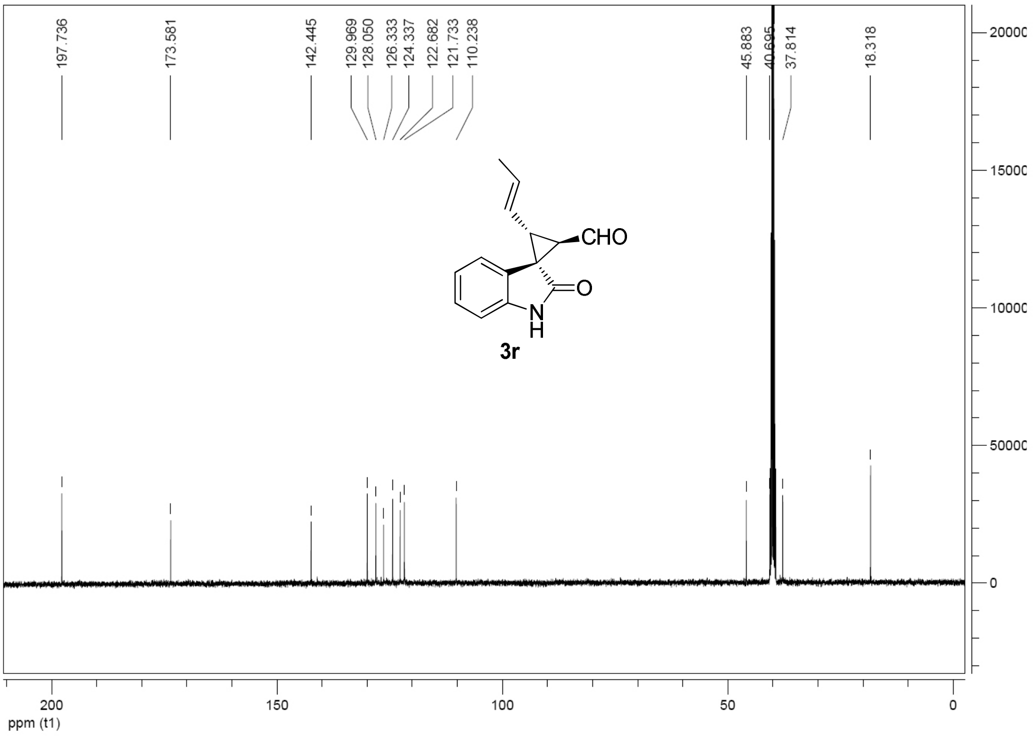

Supplementary Figure 39. ^{13}C NMR spectra of 3r

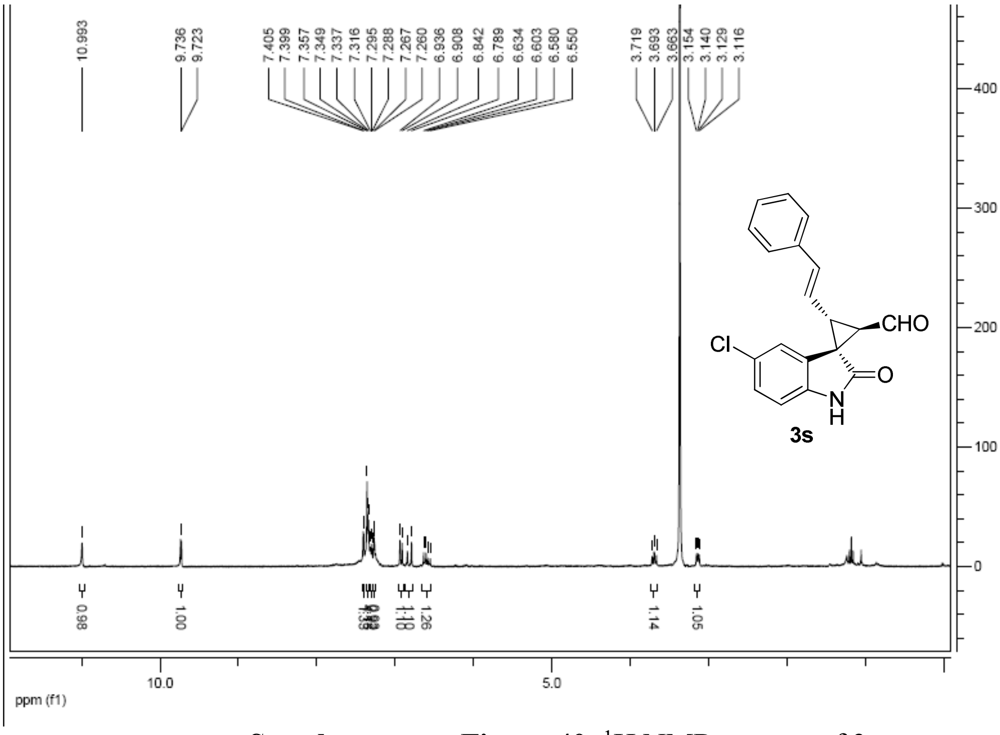

Supplementary Figure 40. ^{1}H NMR spectra of 3s

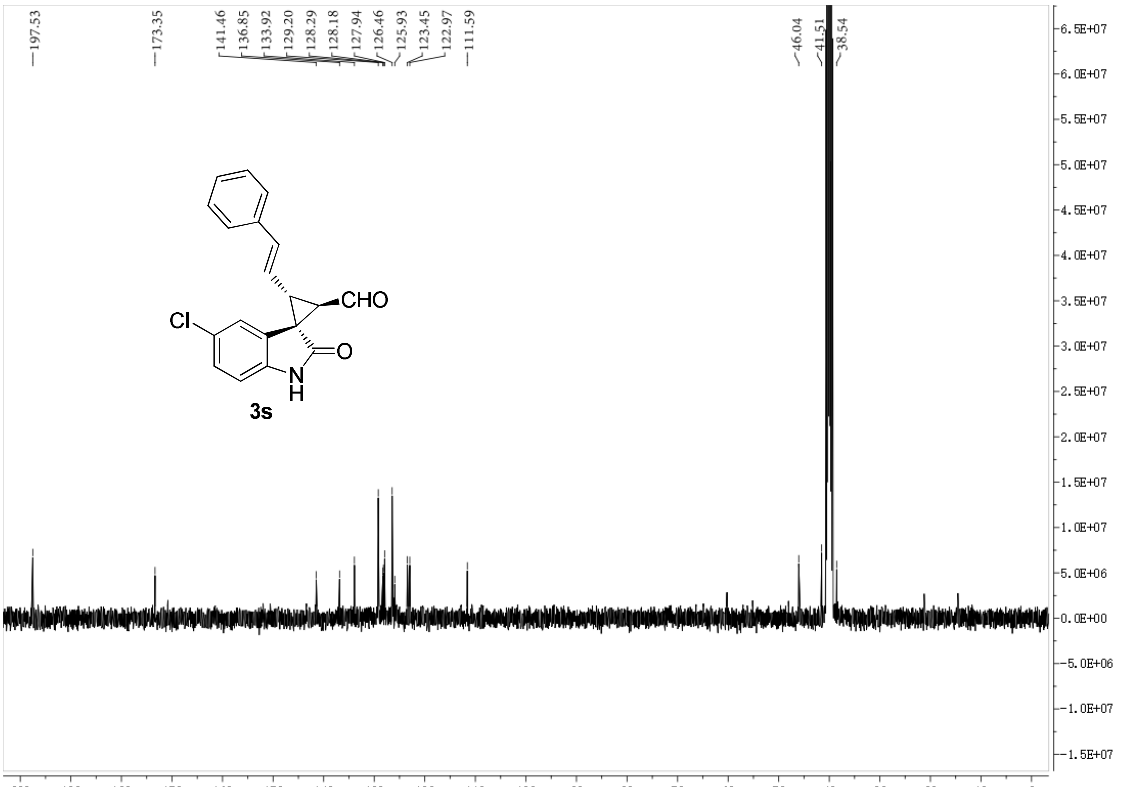

Supplementary Figure 41. ^{13}C NMR spectra of 3s

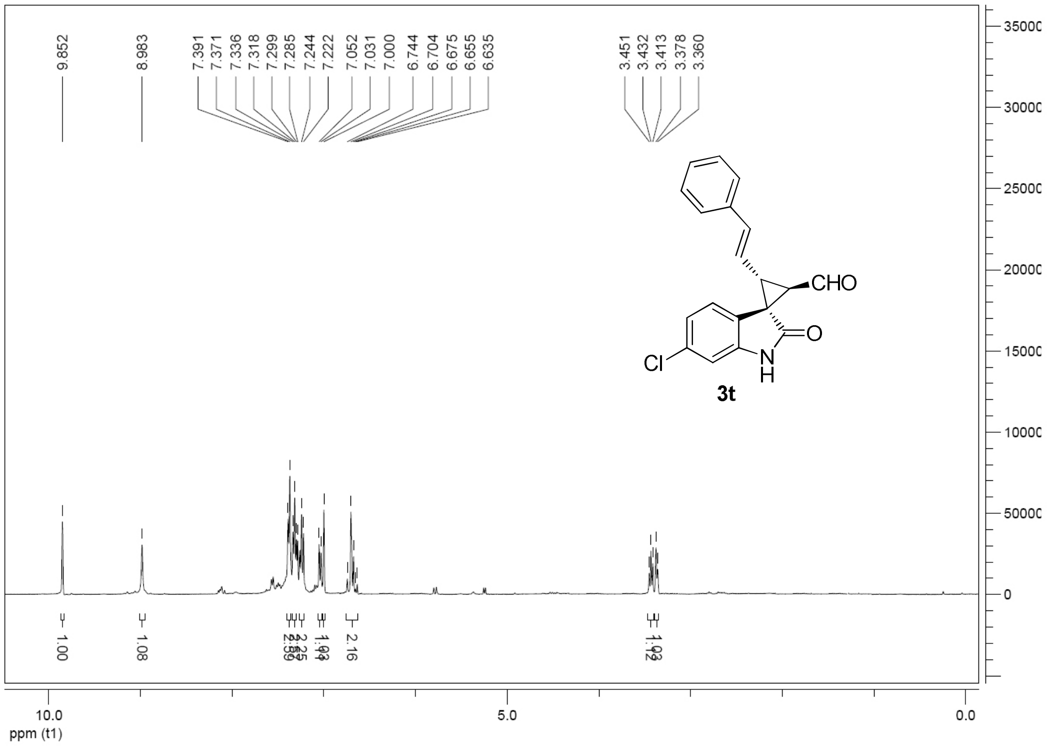

Supplementary Figure 42. ^{1}H NMR spectra of 3t

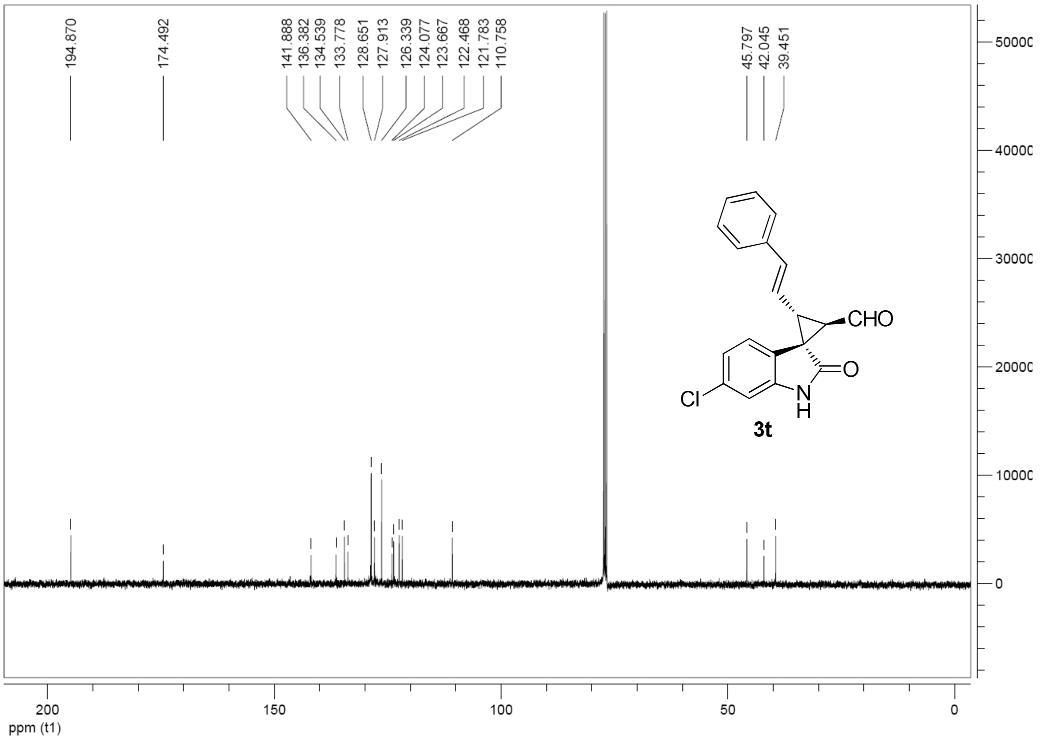

Supplementary Figure 43. ^{13}C NMR spectra of 3t

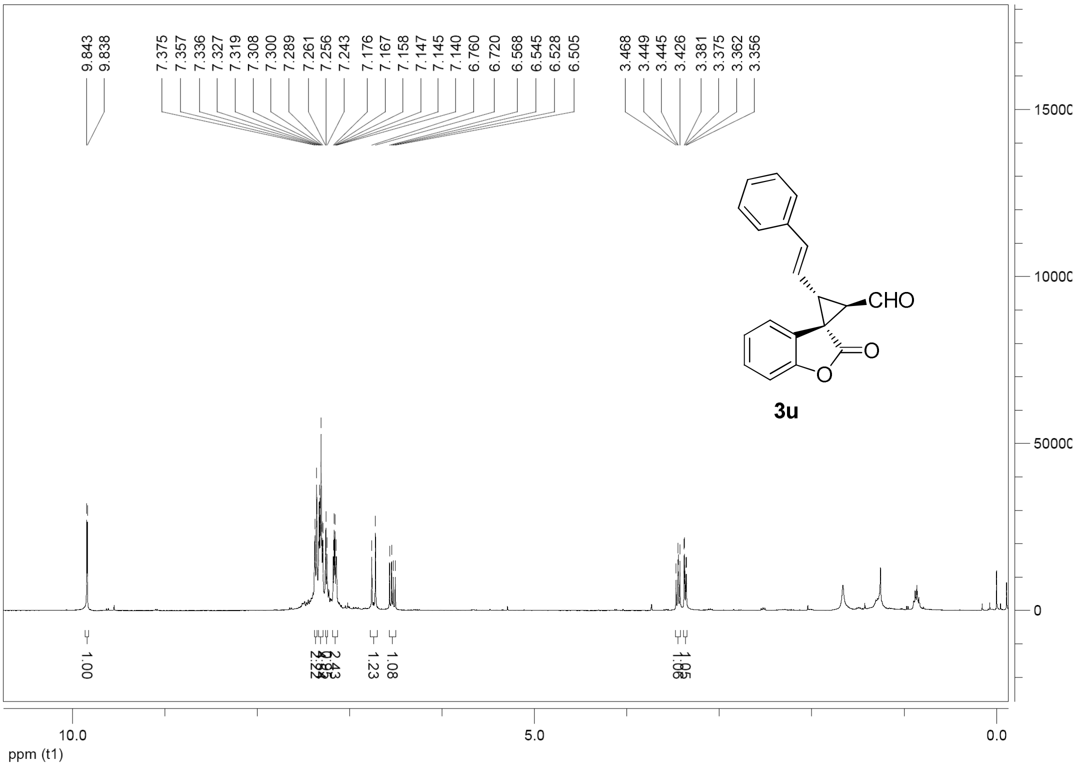

Supplementary Figure 44. ^{1}H NMR spectra of 3u

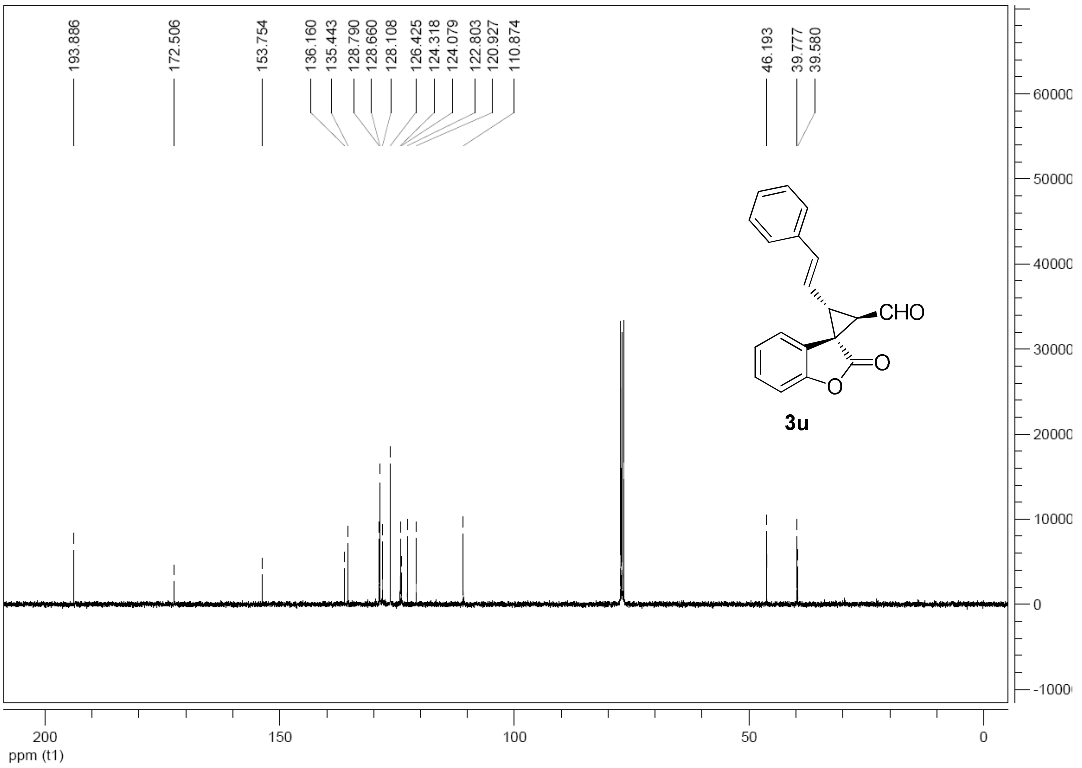

Supplementary Figure 45. ^{13}C NMR spectra of 3u

2.5. HPLC Spectra

All racemic samples of spirocyclopropyloxindoles **3** were prepared using a 1:1 mixture of (*R*,*S*)-diphenylprolinol trimethylsilyl ether (20mol%).

<Chromatogram>

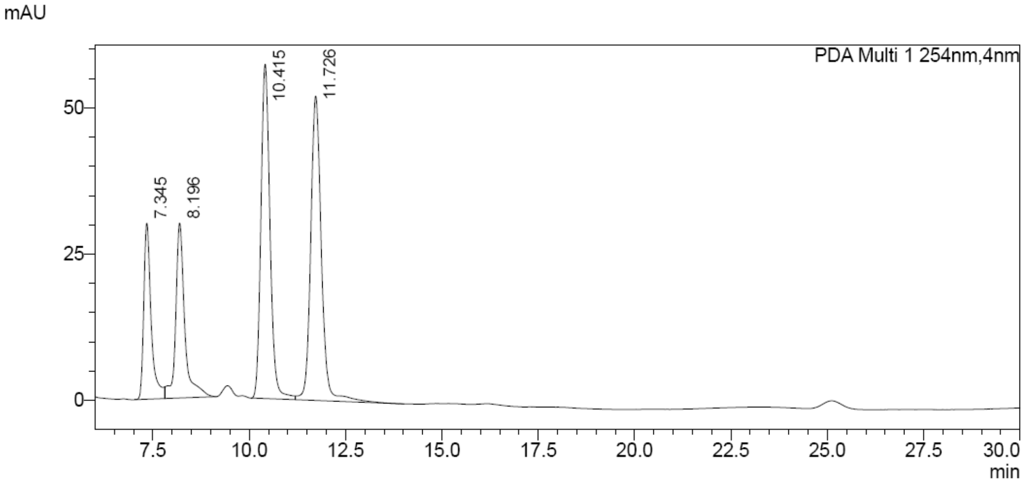

<Peak Table>

PDA Ch1 254nm

Peak#	Ret. Time	Area	Height	Conc.	Unit	Mark	Name
1	7.345	403361	29024	14.229	%		RT:7.345
2	8.196	487221	27549	17.188	%	V	RT:8.196
3	10.415	937410	56074	33.069	%		RT:10.415
4	11.726	1006736	50349	35.514	%	V	RT:11.726
Total		2834728	162996				

Supplementary Figure 46. HPLC spectra of rac-3a

<Chromatogram>

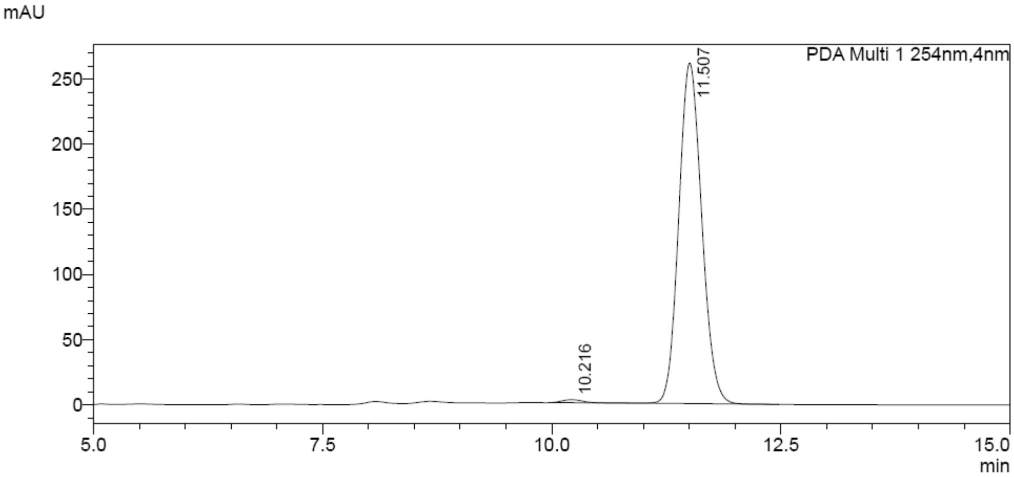

<Peak Table>

PDA Ch1 254nm

Peak#	Ret. Time	Area	Height	Conc.	Unit	Mark	Name
1	10.216	35215	2293	0.767	%		RT:10.216
2	11.507	4557478	245257	99.233	%		RT:11.507
Total		4592693	247550				

Supplementary Figure 47. HPLC spectra of 3a

<Chromatogram>

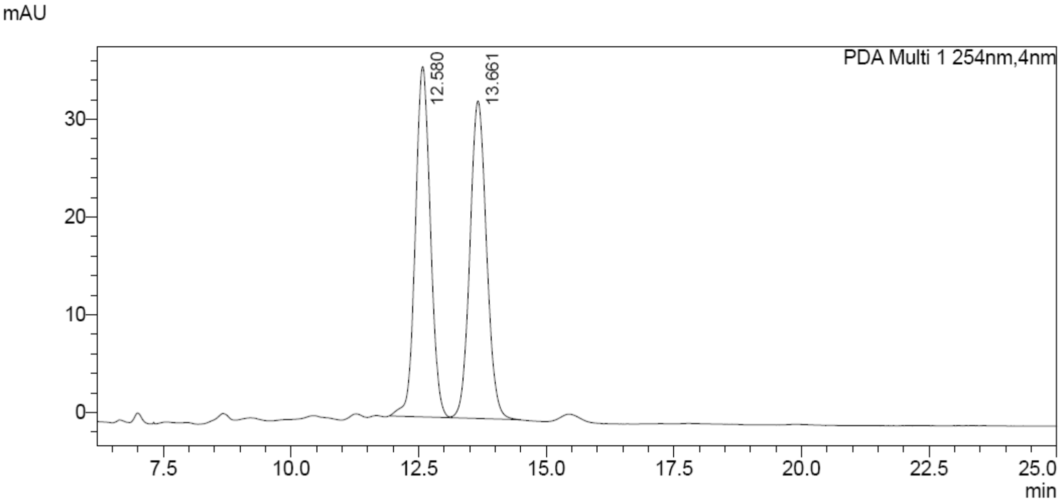

<Peak Table>

PDA Ch1 254nm

Peak#	Ret. Time	Area	Height	Conc.	Unit	Mark	Name
1	12.580	735032	35291	49.989	%		RT:12.580
2	13.661	735362	31502	50.011	%	V	RT:13.661
Total		1470394	66793				

Supplementary Figure 48. HPLC spectra of rac-3b

<Chromatogram>

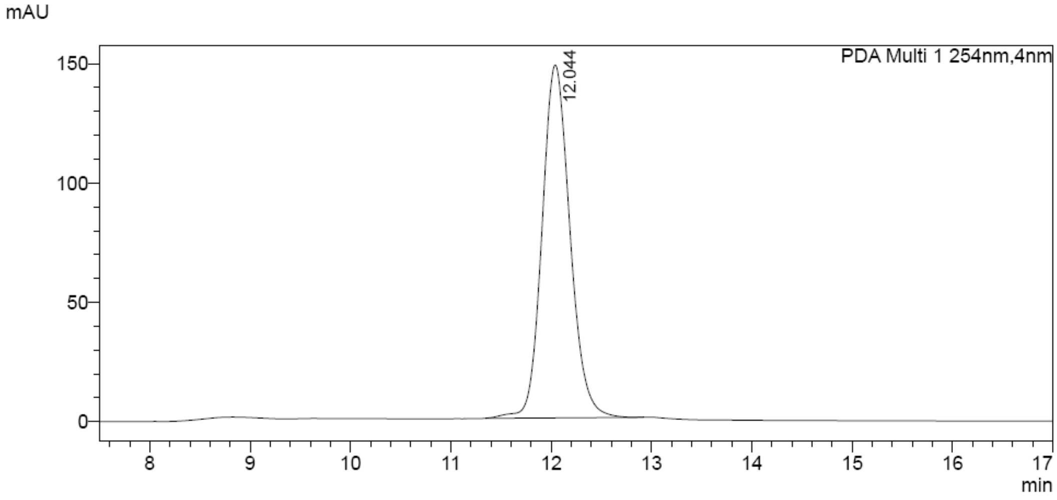

<Peak Table>

PDA Ch1 254nm

Peak#	Ret. Time	Area	Height	Conc.	Unit	Mark	Name
1	12.044	2867764	143380	100.000	%		RT:12.044
Total		2867764	143380				

Supplementary Figure 49. HPLC spectra of 3b

<Chromatogram>

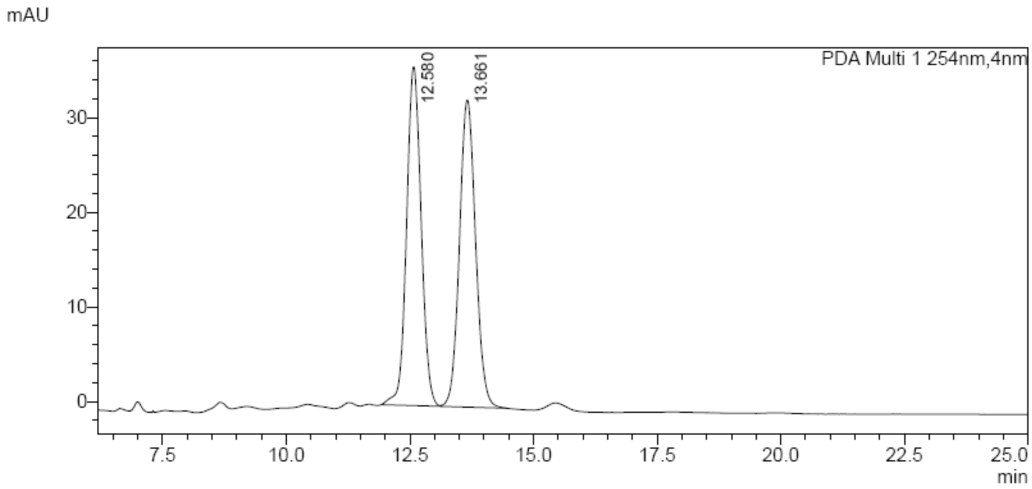

<Peak Table>

PDA Ch1 254nm

Peak#	Ret. Time	Area	Height	Conc.	Unit	Mark	Name
1	12.580	735032	35291	49.989	%		RT:12.580
2	13.661	735362	31502	50.011	%	V	RT:13.661
Total		1470394	66793				

Supplementary Figure 50. HPLC spectra of rac-3c

<Chromatogram>

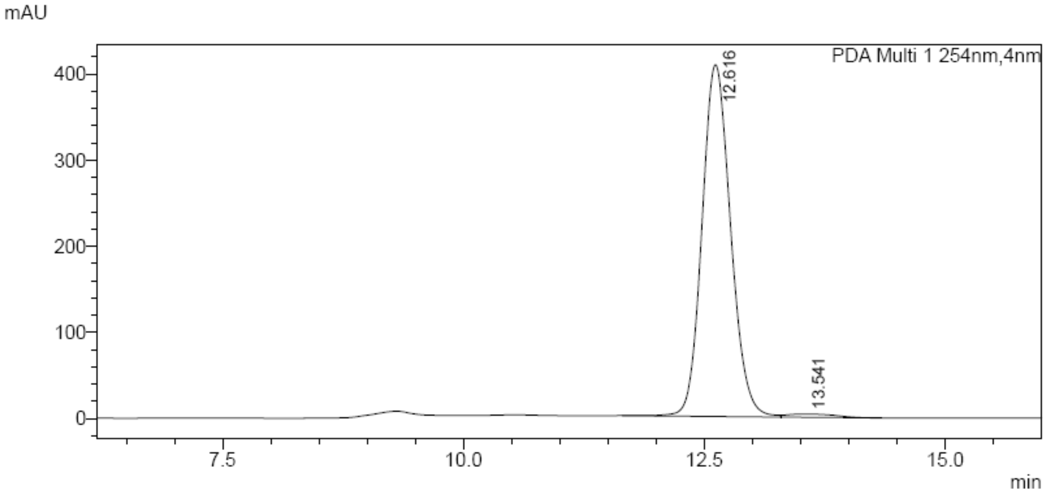

<Peak Table>

PDA Ch1 254nm

Peak#	Ret. Time	Area	Height	Conc.	Unit	Mark	Name
1	12.616	8434867	393842	98.515	%		RT:12.616
2	13.541	127116	3757	1.485	%	V	RT:13.541
Total		8561984	397600				

Supplementary Figure 51. HPLC spectra of 3c

<Chromatogram>

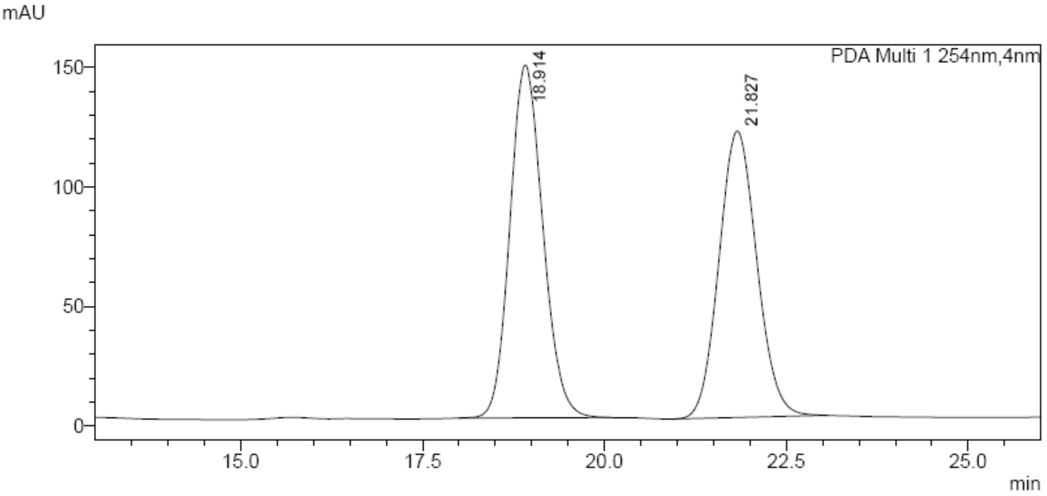

<Peak Table>

PDA Ch1 254nm

Peak#	Ret. Time	Area	Height	Conc.	Unit	Mark	Name
1	18.914	4664434	146221	51.770	%		RT:18.914
2	21.827	4345566	119039	48.230	%		RT:21.827
Total		9010001	265260				

Supplementary Figure 52. HPLC spectra of rac-3d

<Chromatogram>

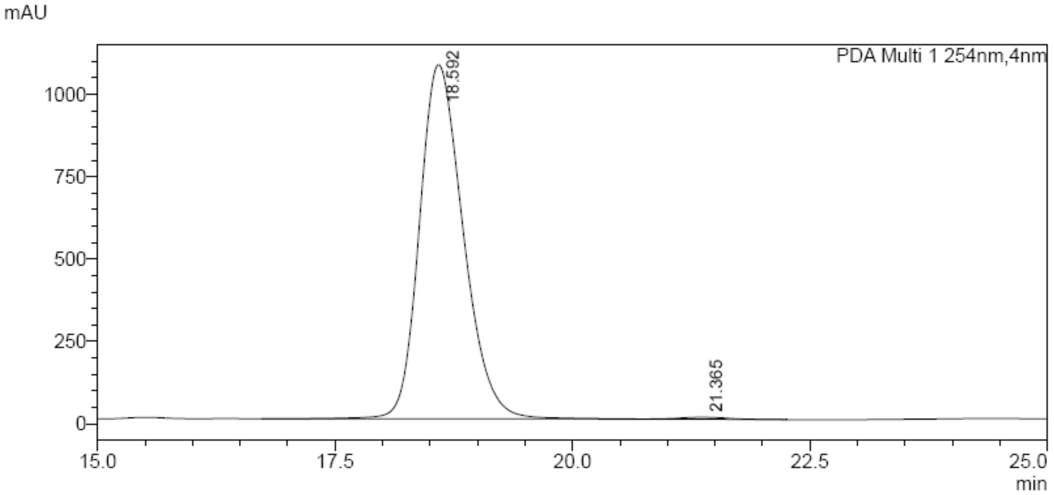

<Peak Table>

PDA Ch1 254nm

Peak#	Ret. Time	Area	Height	Conc.	Unit	Mark	Name
1	18.592	34484052	1055738	99.434	%		RT:18.592
2	21.365	196233	5847	0.566	%		RT:21.365
Total		34680285	1061586				

Supplementary Figure 53. HPLC spectra of 3d

<Chromatogram>

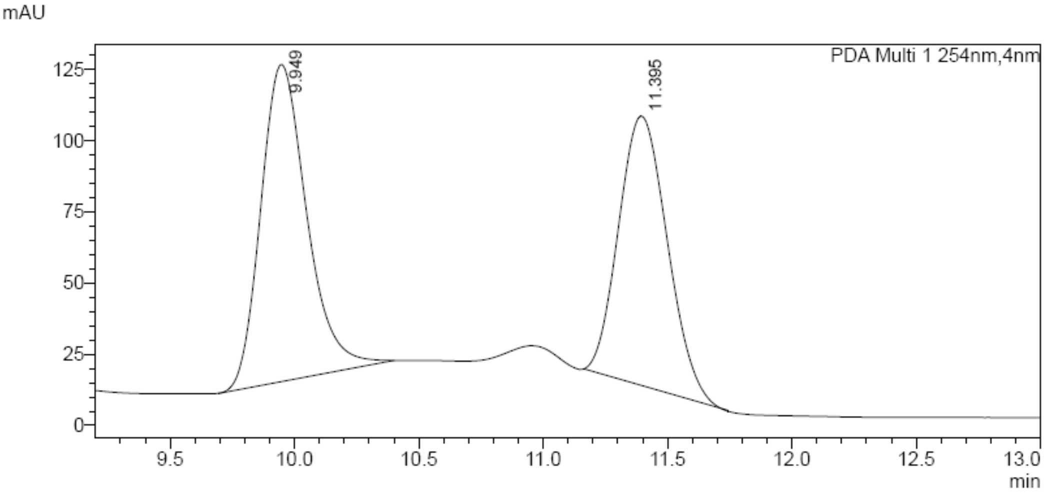

<Peak Table>

PDA Ch1 254nm

Peak#	Ret. Time	Area	Height	Conc.	Unit	Mark	Name
1	9.949	1436434	110447	52.792	%		RT:9.948
2	11.395	1284479	93199	47.208	%		RT:11.395
Total		2720913	203646				

Supplementary Figure 54. HPLC spectra of rac-3e

<Chromatogram>

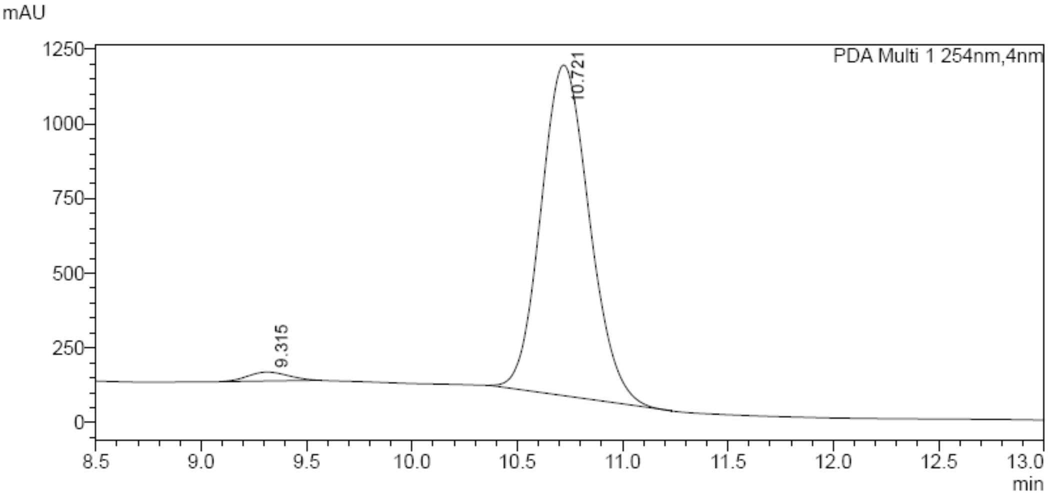

<Peak Table>

PDA Ch1 254nm

Peak#	Ret. Time	Area	Height	Conc.	Unit	Mark	Name
1	9.315	399804	29450	2.203	%		RT:9.315
2	10.721	17749510	1097361	97.797	%		RT:10.721
Total		18149314	1126811				

Supplementary Figure 55. HPLC spectra of 3e

<Chromatogram>

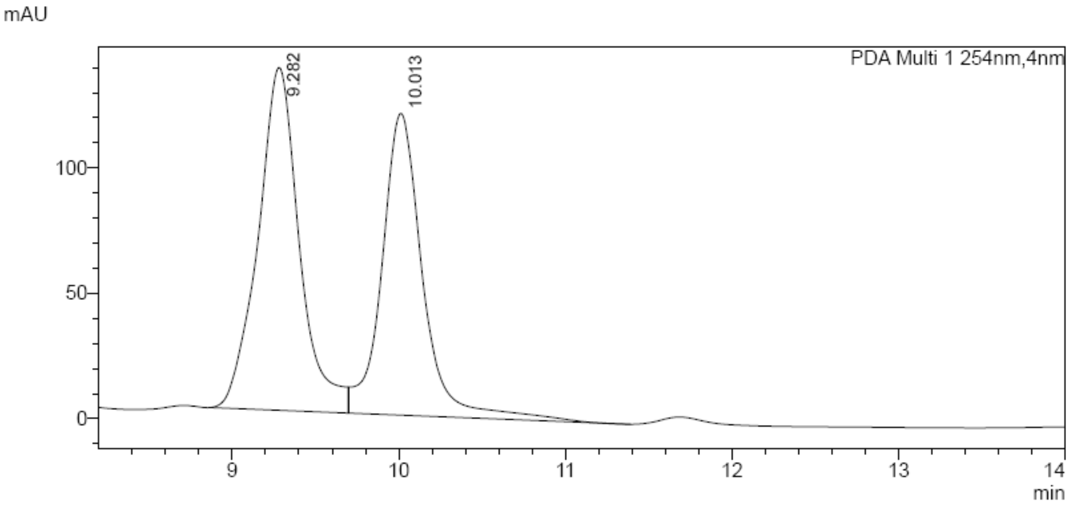

<Peak Table>

PDA Ch1 254nm

Peak#	Ret. Time	Area	Height	Conc.	Unit	Mark	Name
1	9.282	2359449	135101	53.589	%		RT:9.282
2	10.013	2043448	119597	46.411	%	V	RT:10.013
Total		4402897	254698				

Supplementary Figure 56. HPLC spectra of rac-3f

<Chromatogram>

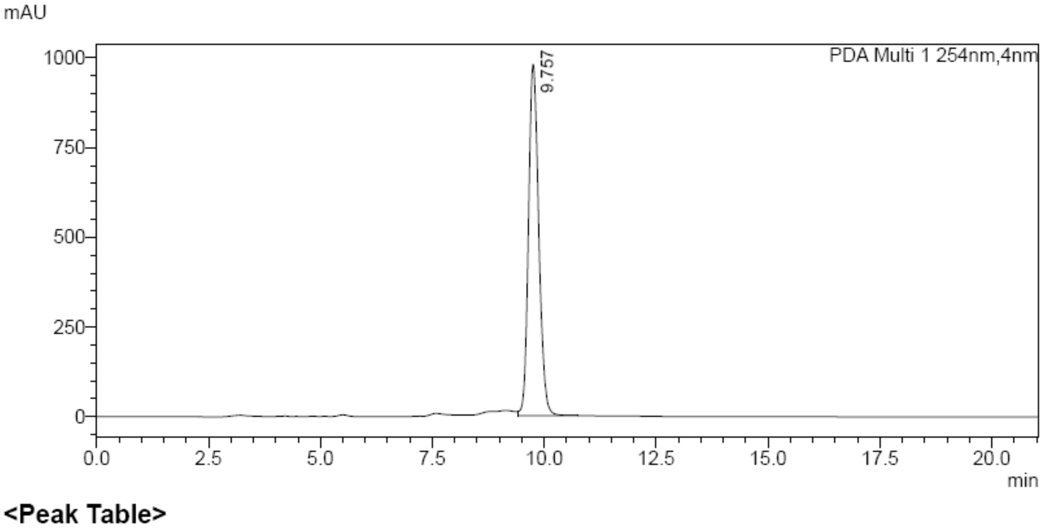

<Peak Table>

PDA Ch1 254nm

Peak#	Ret. Time	Area	Height	Conc.	Unit	Mark	Name
1	9.757	15612732	958500	100.000	%	V	RT:9.757
Total		15612732	958500				

Supplementary Figure 57. HPLC spectra of 3f

<Chromatogram>

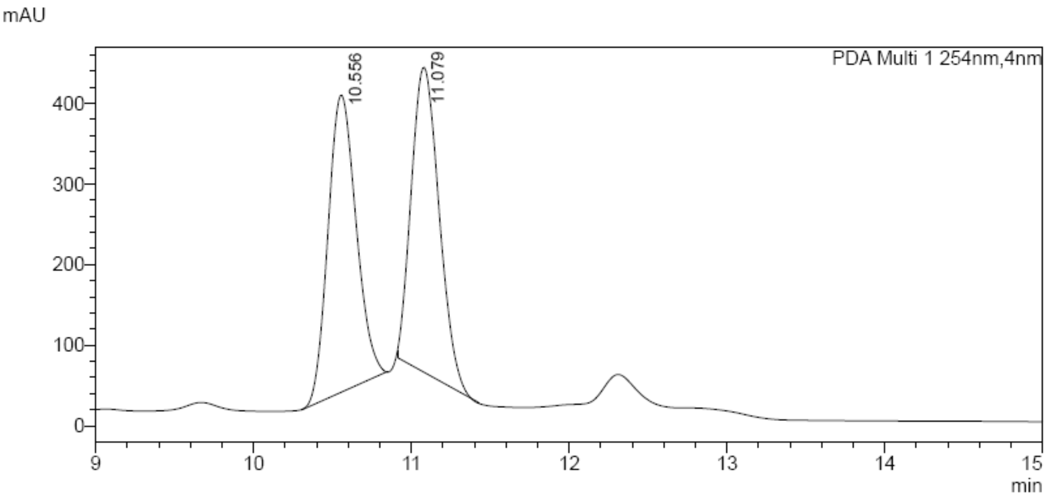

<Peak Table>

PDA Ch1 254nm

Peak#	Ret. Time	Area	Height	Conc.	Unit	Mark	Name
1	10.556	4578091	365757	49.820	%		RT:10.556
2	11.079	4611189	371061	50.180	%		RT:11.079
Total		9189280	736818				

Supplementary Figure 58. HPLC spectra of rac-3g

<Chromatogram>

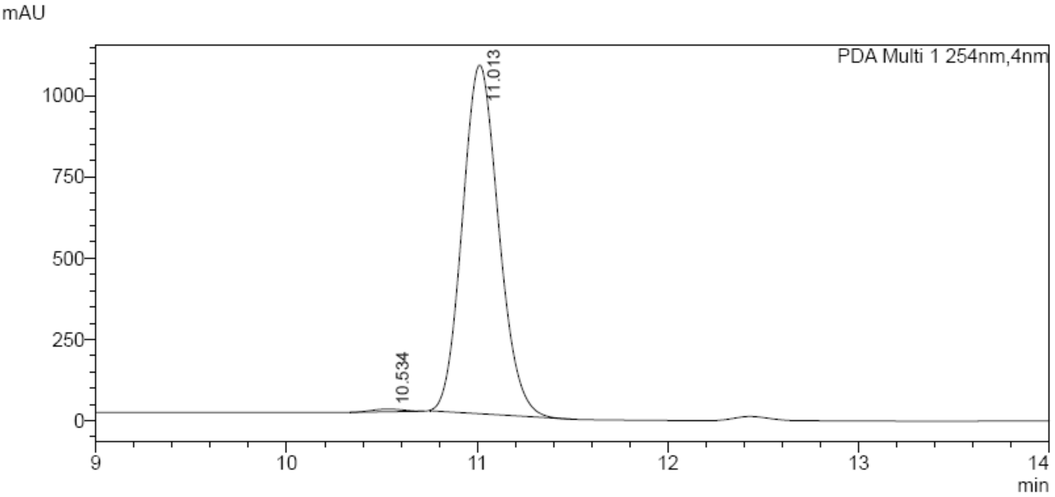

<Peak Table>

PDA Ch1 254nm

Peak#	Ret. Time	Area	Height	Conc.	Unit	Mark	Name
1	10.534	94745	8200	0.661	%		RT:10.534
2	11.013	14230158	1051206	99.339	%		RT:11.013
Total		14324903	1059406				

Supplementary Figure 59. HPLC spectra of 3g

<Chromatogram>

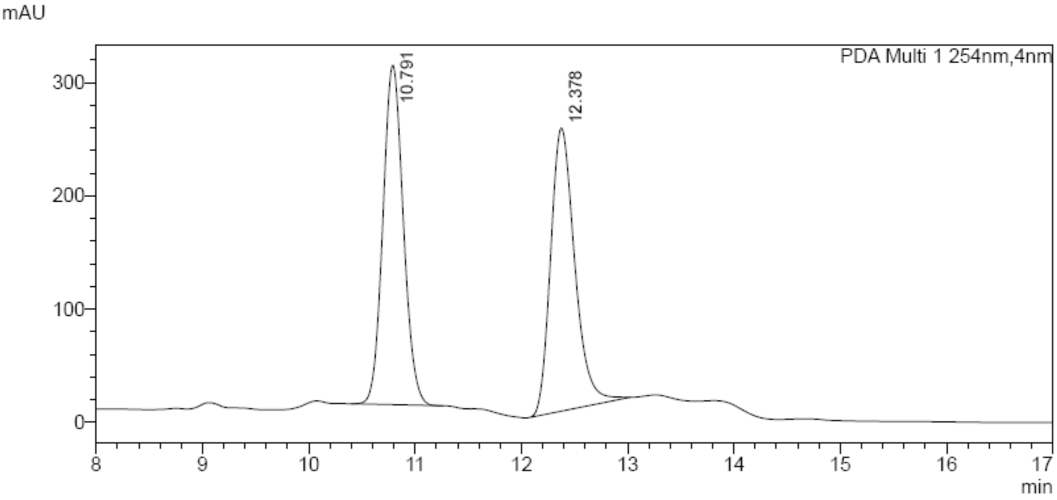

<Peak Table>

PDA Ch1 254nm

Peak#	Ret. Time	Area	Height	Conc.	Unit	Mark	Name
1	10.791	4009783	294257	50.302	%		RT:10.791
2	12.378	3961648	249031	49.698	%		RT:12.378
Total		7971431	543289				

Supplementary Figure 60. HPLC spectra of rac-3h

<Chromatogram>

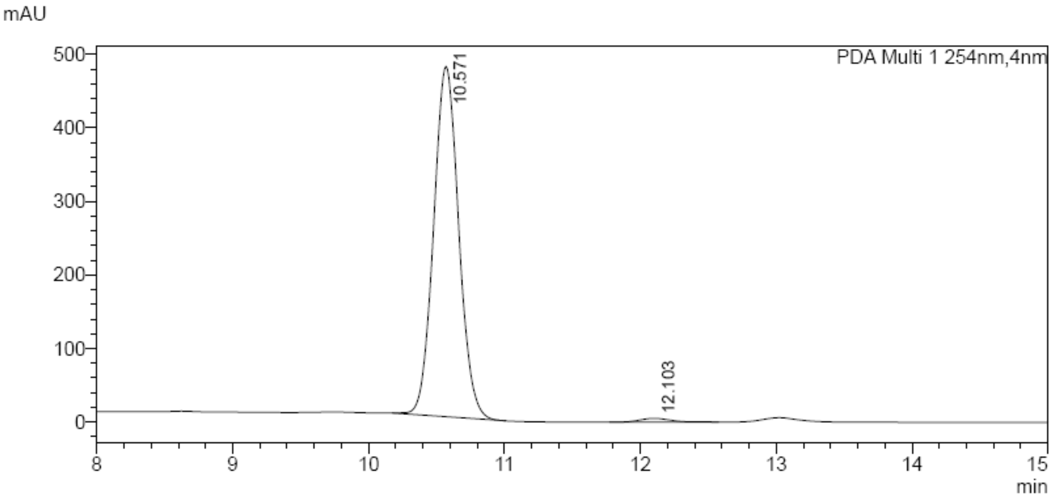

<Peak Table>

PDA Ch1 254nm

Peak#	Ret. Time	Area	Height	Conc.	Unit	Mark	Name
1	10.571	6102323	469848	98.710	%		RT:10.571
2	12.103	79778	5037	1.290	%		RT:12.103
Total		6182101	474885				

Supplementary Figure 61. HPLC spectra of 3h

<Chromatogram>

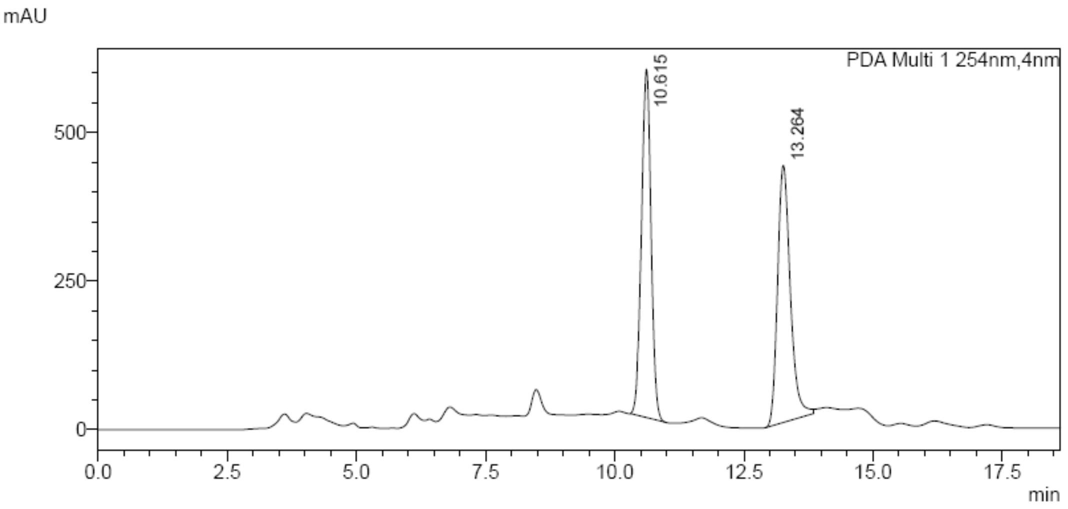

<Peak Table>

PDA Ch1 254nm

Peak#	Ret. Time	Area	Height	Conc.	Unit	Mark	Name
1	10.615	7570306	583158	50.456	%		RT:10.615
2	13.264	7433563	430929	49.544	%		RT:13.264
Total		15003869	1014087				

Supplementary Figure 62. HPLC spectra of rac-3i

<Chromatogram>

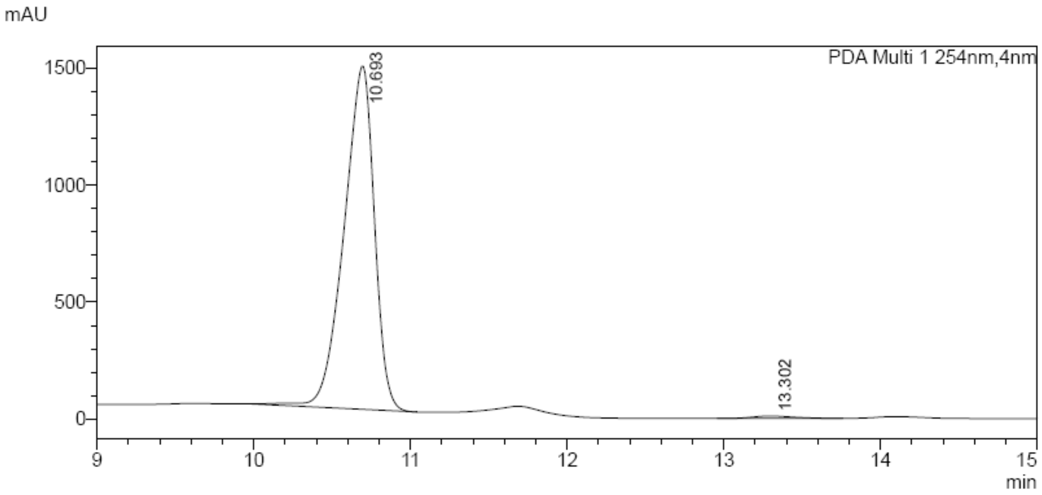

<Peak Table>

PDA Ch1 254nm

Peak#	Ret. Time	Area	Height	Conc.	Unit	Mark	Name
1	10.693	19783881	1431525	99.064	%		RT:10.693
2	13.302	186998	10360	0.936	%		RT:13.302
Total		19970879	1441885				

Supplementary Figure 63. HPLC spectra of 3i

<Chromatogram>

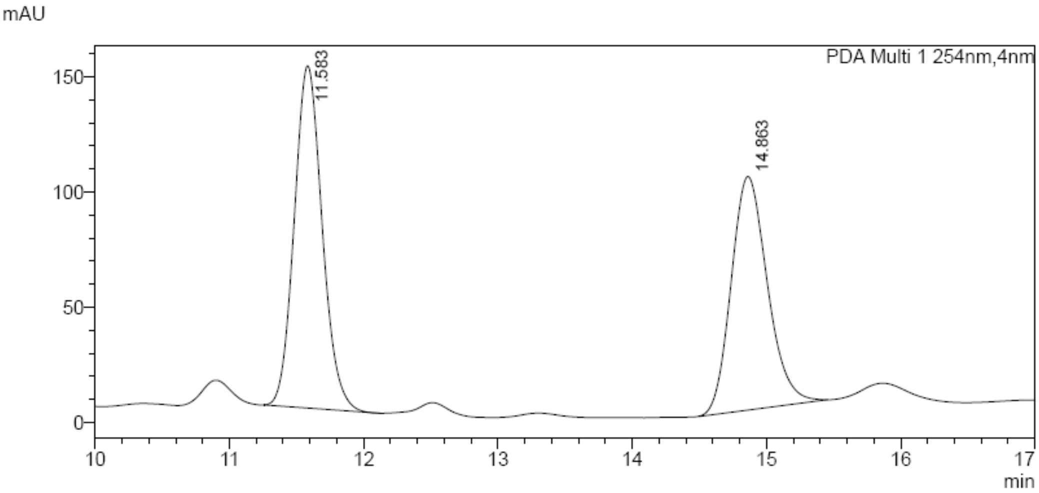

<Peak Table>

PDA Ch1 254nm

Peak#	Ret. Time	Area	Height	Conc.	Unit	Mark	Name
1	11.583	2150179	147083	53.040	%		RT:11.583
2	14.863	1903673	101001	46.960	%		RT:14.863
Total		4053852	248085				

Supplementary Figure 64. HPLC spectra of rac-3j

<Chromatogram>

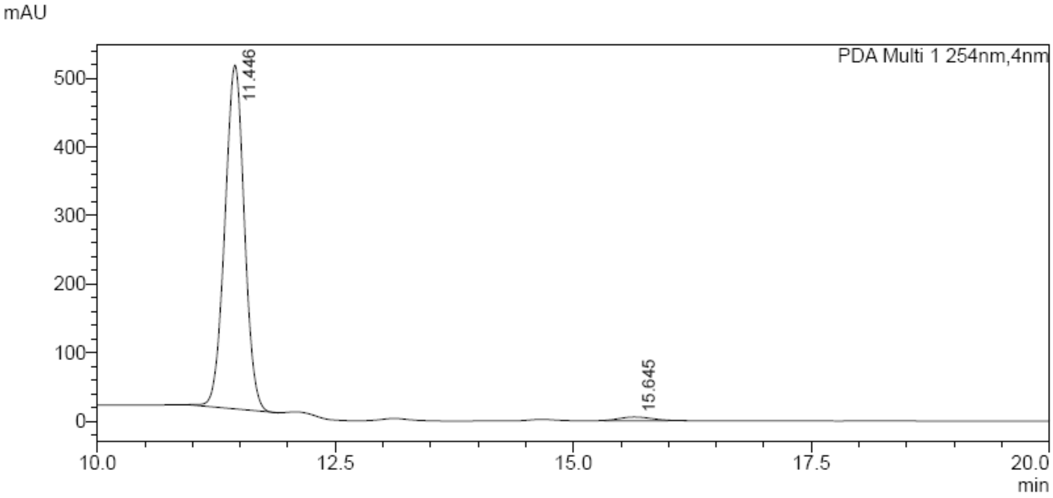

<Peak Table>

PDA Ch1 254nm

Peak#	Ret. Time	Area	Height	Conc.	Unit	Mark	Name
1	11.446	7155167	499622	98.252	%		RT:11.445
2	15.645	127329	5332	1.748	%	V	RT:15.645
Total		7282496	504954				

Supplementary Figure 65. HPLC spectra of 3j

<Chromatogram>

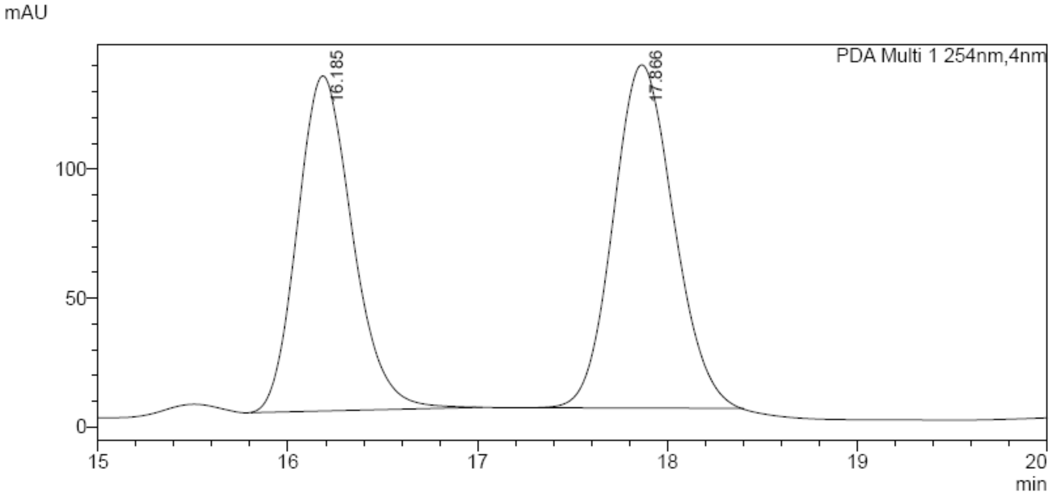

<Peak Table>

PDA Ch1 254nm

Peak#	Ret. Time	Area	Height	Conc.	Unit	Mark	Name
1	16.185	2628940	129634	47.072	%		RT:16.185
2	17.866	2956019	132454	52.928	%		RT:17.866
Total		5584959	262088				

Supplementary Figure 66. HPLC spectra of rac-3k

<Chromatogram>

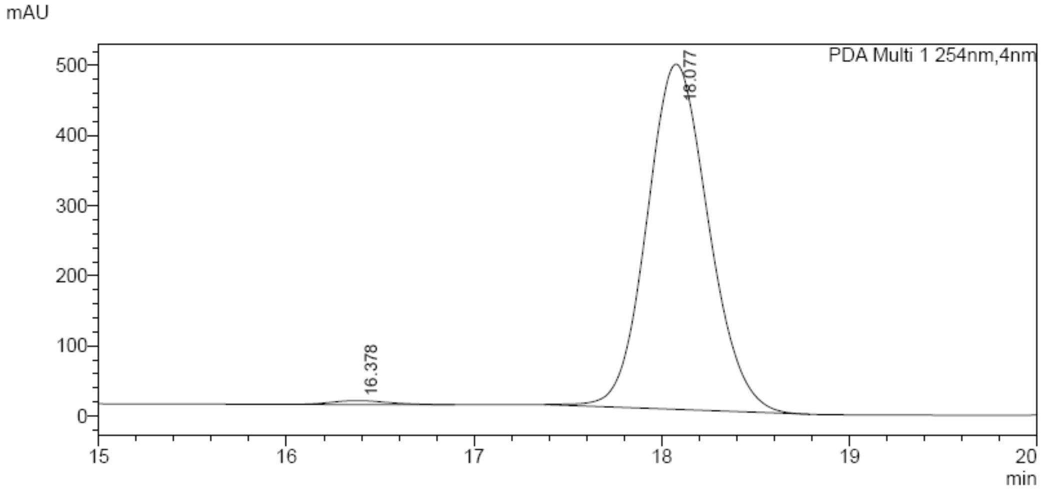

<Peak Table>

PDA Ch1 254nm

Peak#	Ret. Time	Area	Height	Conc.	Unit	Mark	Name
1	16.378	111926	5524	0.984	%		RT:16.378
2	18.077	11259083	491105	99.016	%		RT:18.077
Total		11371009	496629				

Supplementary Figure 67. HPLC spectra of 3k

<Chromatogram>

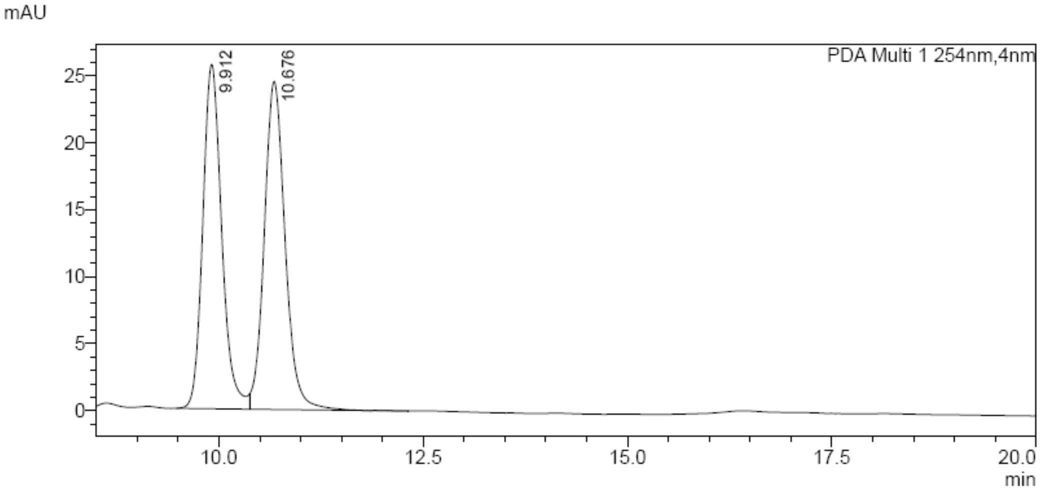

<Peak Table>

PDA Ch1 254nm

Peak#	Ret. Time	Area	Height	Conc.	Unit	Mark	Name
1	9.912	415494	24970	48.950	%		RT:9.912
2	10.676	433314	23155	51.050	%	V	RT:10.676
Total		848808	48125				

Supplementary Figure 68. HPLC spectra of rac-3l

<Chromatogram>

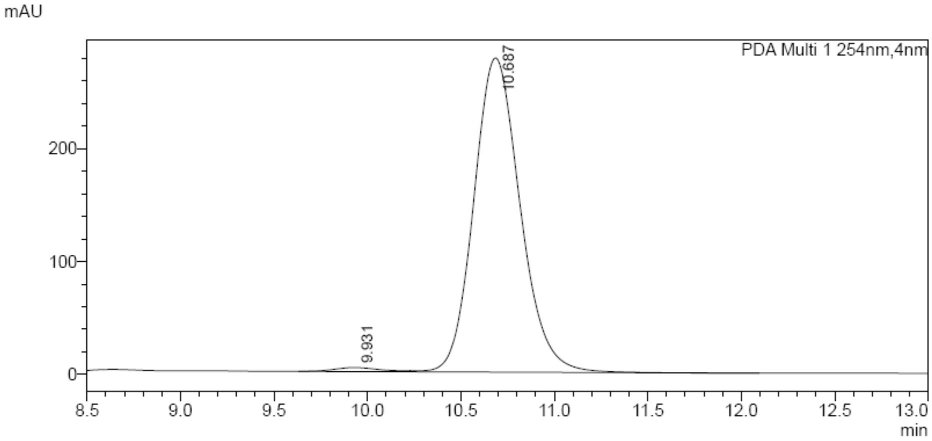

<Peak Table>

PDA Ch1 254nm

Peak#	Ret. Time	Area	Height	Conc.	Unit	Mark	Name
1	9.931	61200	3364	1.256	%		RT:9.931
2	10.687	4811197	261534	98.744	%	V	RT:10.687
Total		4872397	264899				

Supplementary Figure 69. HPLC spectra of 3l

<Chromatogram>

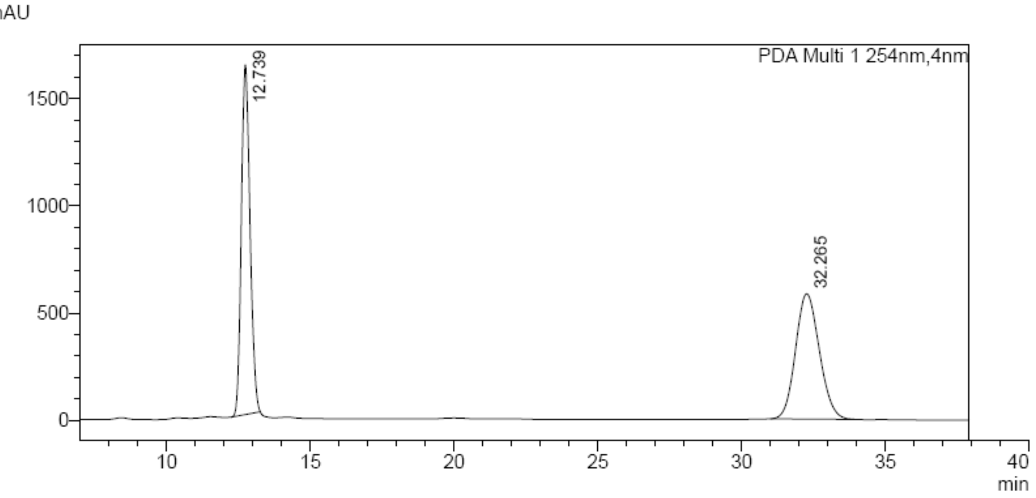

<Peak Table>

PDA Ch1 254nm

Peak#	Ret. Time	Area	Height	Conc.	Unit	Mark	Name
1	12.739	34299857	1630831	50.435	%		RT:12.739
2	32.265	33708314	584585	49.565	%		RT:32.265
Total		68008170	2215416				

Supplementary Figure 70. HPLC spectra of rac-3m

<Chromatogram>

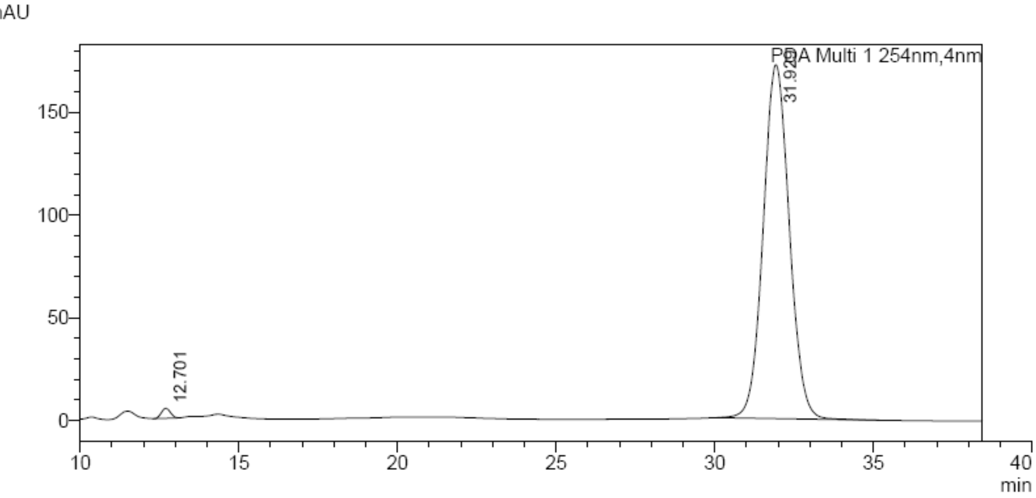

<Peak Table>

PDA Ch1 254nm

Peak#	Ret. Time	Area	Height	Conc.	Unit	Mark	Name
1	12.701	97247	4905	0.976	%		RT:12.701
2	31.929	9869048	172114	99.024	%		RT:31.929
Total		9966295	177019				

Supplementary Figure 71. HPLC spectra of 3m

<Chromatogram>

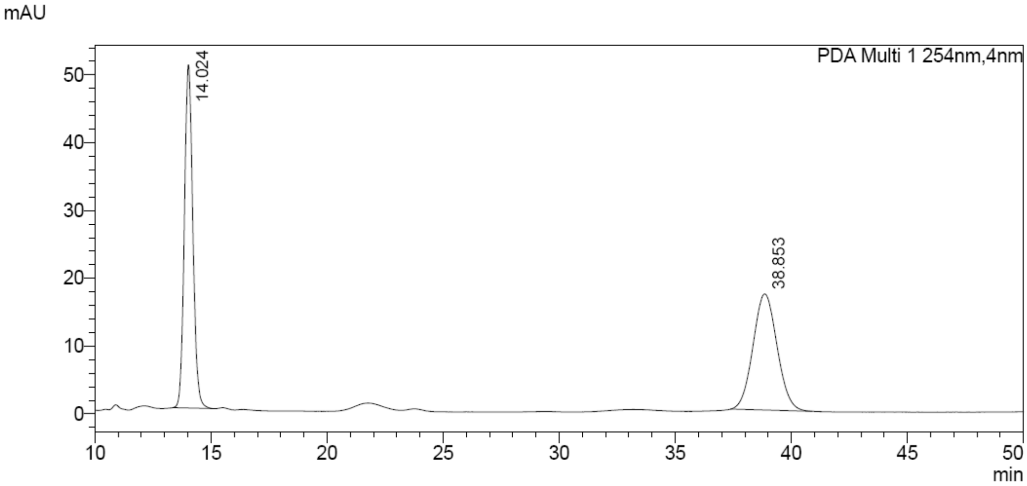

<Peak Table>

PDA Ch1 254nm

Peak#	Ret. Time	Area	Height	Conc.	Unit	Mark	Name
1	14.024	1284227	50643	50.970	%		RT:14.024
2	38.853	1235352	17111	49.030	%		RT:38.853
Total		2519578	67755				

Supplementary Figure 72. HPLC spectra of rac-3n

<Chromatogram>

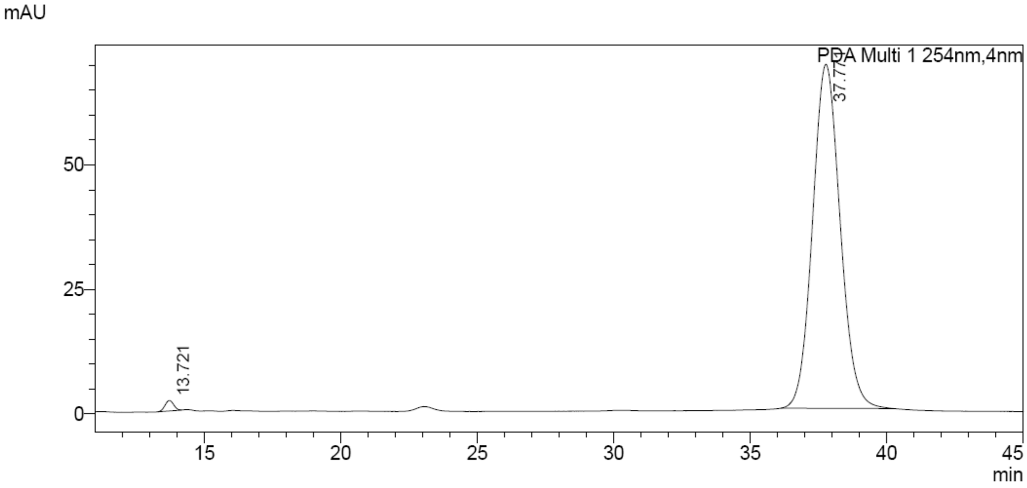

<Peak Table>

PDA Ch1 254nm

Peak#	Ret. Time	Area	Height	Conc.	Unit	Mark	Name
1	13.721	46183	2102	0.942	%		RT:13.721
2	37.771	4857376	69060	99.058	%		RT:37.771
Total		4903559	71163				

Supplementary Figure 73. HPLC spectra of 3n

<Chromatogram>

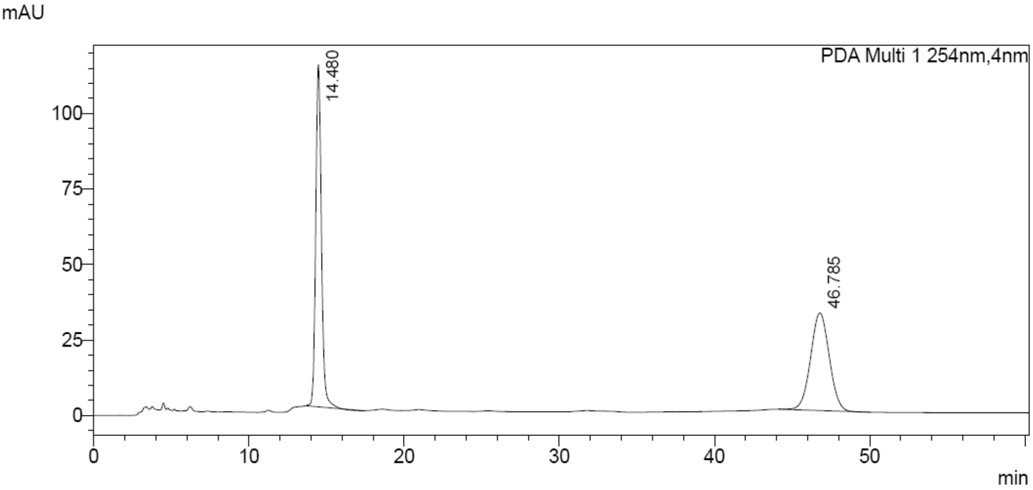

<Peak Table>

PDA Ch1 254nm

Peak#	Ret. Time	Area	Height	Conc.	Unit	Mark	Name
1	14.480	2869375	81305	51.535	%		RT:14.480
2	46.785	2698416	31915	48.465	%		RT:46.785
Total		5567792	113219				

Supplementary Figure 74. HPLC spectra of rac-3o

<Chromatogram>

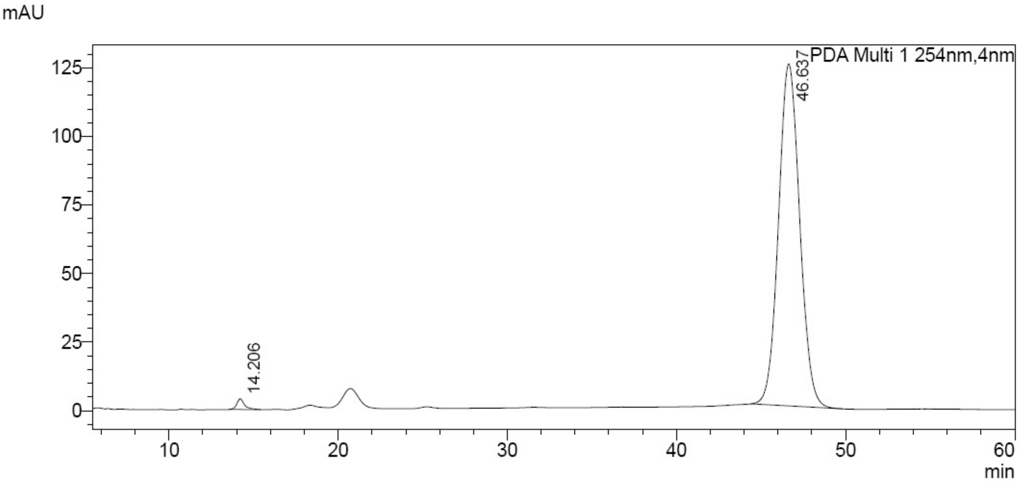

<Peak Table>

PDA Ch1 254nm

Peak#	Ret. Time	Area	Height	Conc.	Unit	Mark	Name
1	14.206	124533	3905	1.133	%		RT:14.206
2	46.637	10867213	124718	98.867	%		RT:46.637
Total		10991745	128623				

Supplementary Figure 75. HPLC spectra of 3o

<Chromatogram>

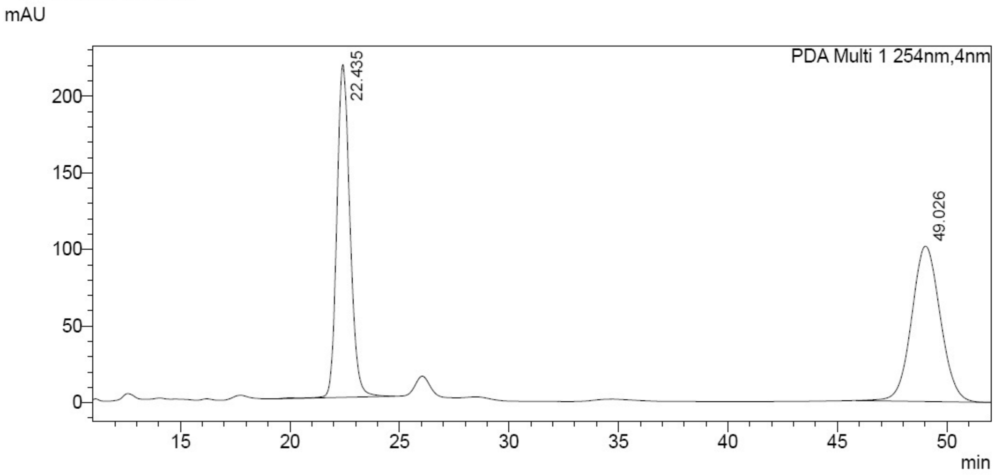

<Peak Table>

PDA Ch1 254nm

Peak#	Ret. Time	Area	Height	Conc.	Unit	Mark	Name
1	22.435	9047144	208157	49.501	%		RT:22.435
2	49.026	9229560	99861	50.499	%		RT:49.026
Total		18276704	308018				

Supplementary Figure 76. HPLC spectra of rac-3p

<Chromatogram>

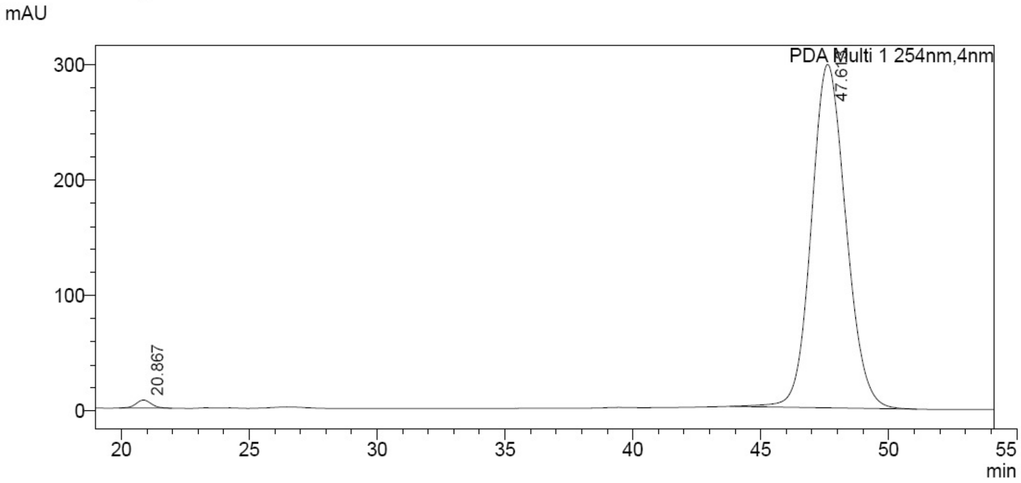

<Peak Table>

PDA Ch1 254nm

Peak#	Ret. Time	Area	Height	Conc.	Unit	Mark	Name
1	20.867	284215	6900	1.004	%		RT:20.867
2	47.613	28037123	297712	98.996	%		RT:47.613
Total		28321338	304612				

Supplementary Figure 77. HPLC spectra of 3p

<Chromatogram>

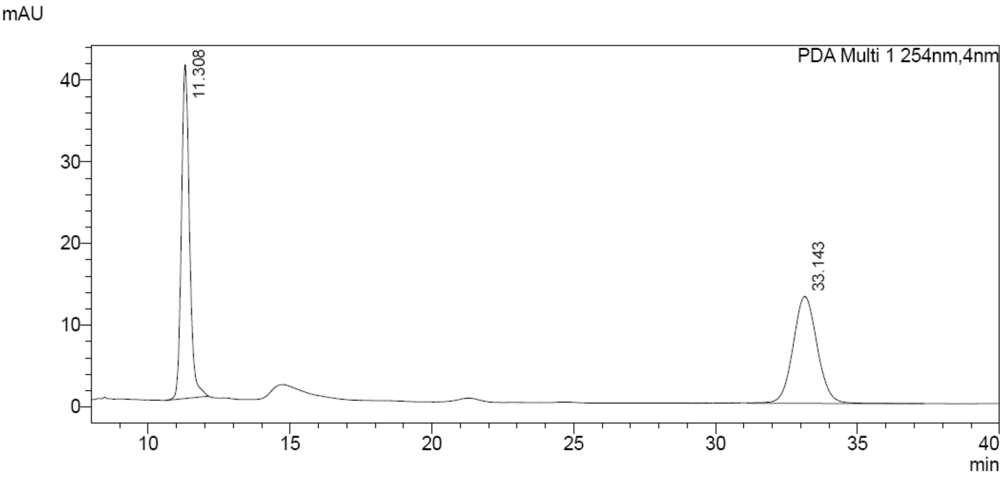

<Peak Table>

PDA Ch1 254nm

Peak#	Ret. Time	Area	Height	Conc.	Unit	Mark	Name
1	11.308	806274	40812	50.999	%		RT:11.308
2	33.143	774673	13062	49.001	%	V	RT:33.143
Total		1580948	53874				

Supplementary Figure 78. HPLC spectra of rac-3q

<Chromatogram>

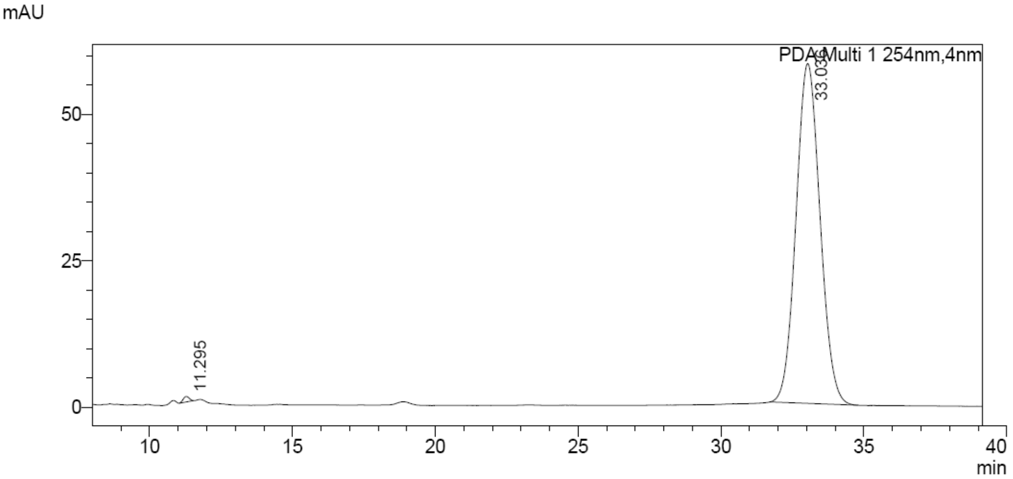

<Peak Table>

PDA Ch1 254nm

Peak#	Ret. Time	Area	Height	Conc.	Unit	Mark	Name
1	11.295	13725	925	0.403	%		RT:11.295
2	33.036	3388659	57972	99.597	%		RT:33.035
Total		3402384	58897				

Supplementary Figure 79. HPLC spectra of 3q

<Chromatogram>

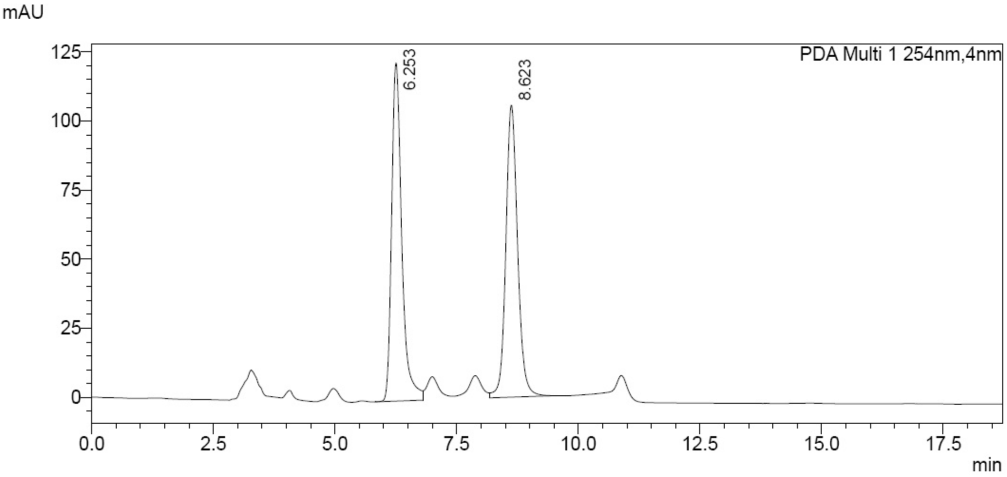

<Peak Table>

PDA Ch1 254nm

Peak#	Ret. Time	Area	Height	Conc.	Unit	Mark	Name
1	6.253	1775759	121912	49.983	%		RT:6.253
2	8.623	1776981	105166	50.017	%	V	RT:8.623
Total		3552740	227078				

Supplementary Figure 80. HPLC spectra of rac-3r

<Chromatogram>

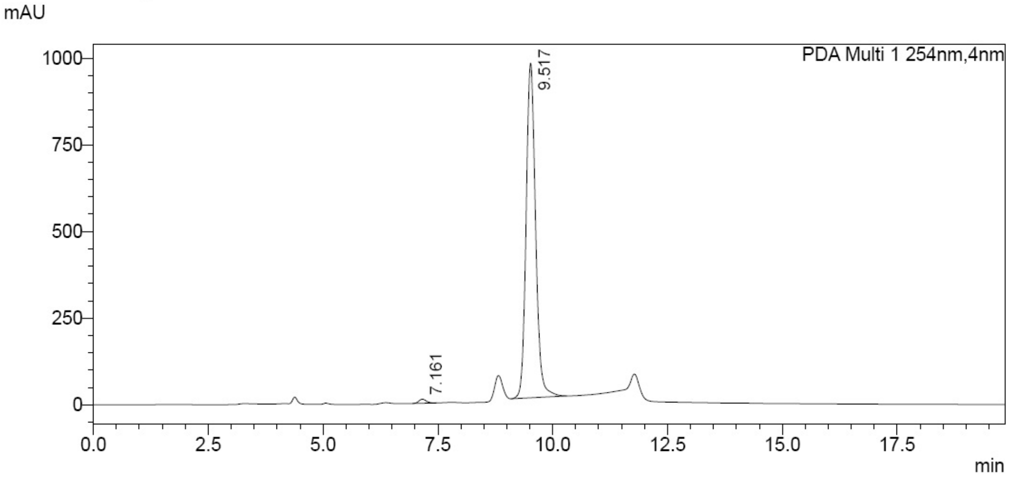

<Peak Table>

PDA Ch1 254nm

Peak#	Ret. Time	Area	Height	Conc.	Unit	Mark	Name
1	7.161	130890	11599	0.938	%		RT:7.161
2	9.517	13827312	960334	99.062	%		RT:9.517
Total		13958202	971933				

Supplementary Figure 81. HPLC spectra of 3r

<Chromatogram>

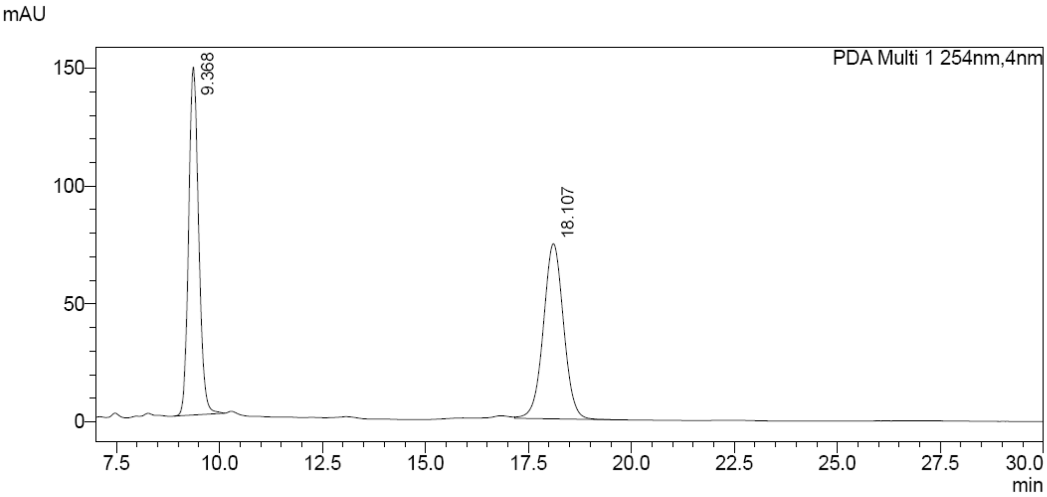

<Peak Table>

PDA Ch1 254nm

Peak#	Ret. Time	Area	Height	Conc.	Unit	Mark	Name
1	9.368	2502985	147547	49.694	%		RT:9.368
2	18.107	2533813	74226	50.306	%	V	RT:18.107
Total		5036798	221773				

Supplementary Figure 82. HPLC spectra of rac-3s

<Chromatogram>

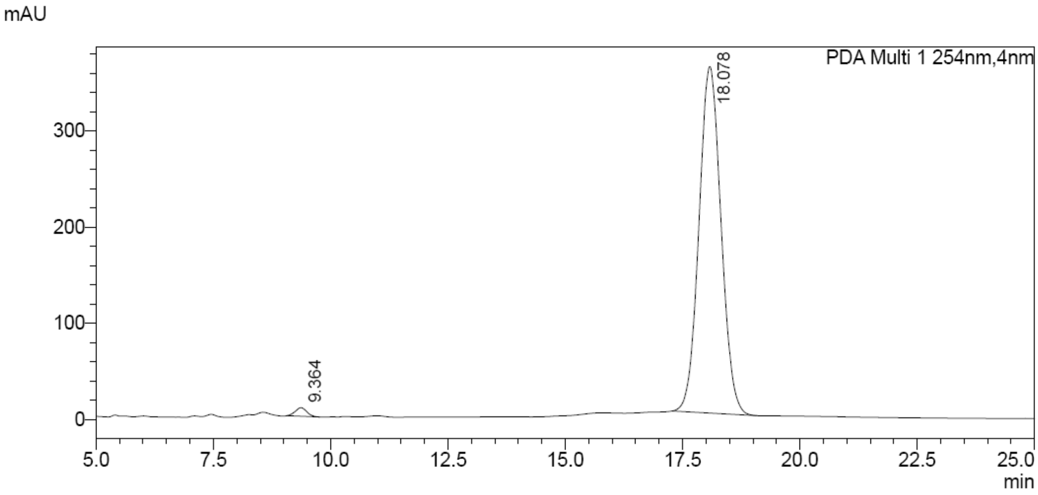

<Peak Table>

PDA Ch1 254nm

Peak#	Ret. Time	Area	Height	Conc.	Unit	Mark	Name
1	9.364	150359	8862	1.259	%		RT:9.364
2	18.078	11795761	360522	98.741	%		RT:18.078
Total		11946120	369384				

Supplementary Figure 83. HPLC spectra of 3s

<Chromatogram>

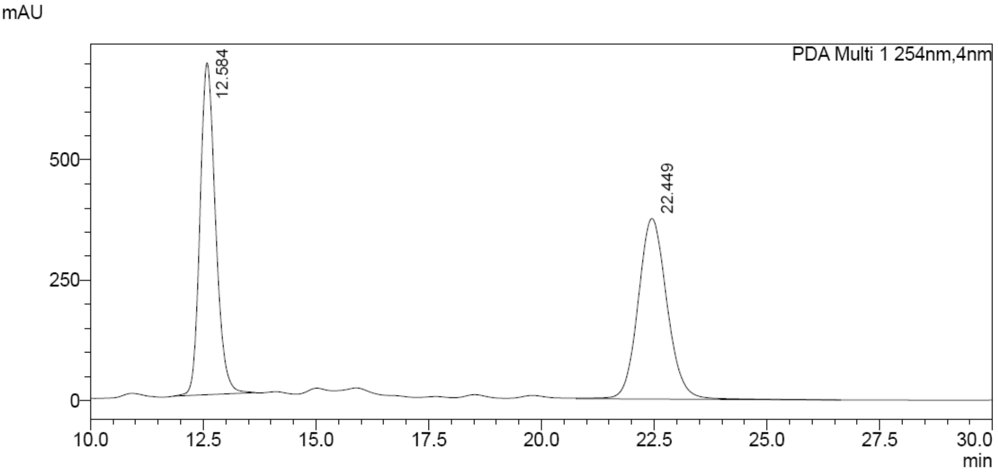

<Peak Table>

PDA Ch1 254nm

Peak#	Ret. Time	Area	Height	Conc.	Unit	Mark	Name
1	12.584	16491438	687383	49.664	%		RT:12.584
2	22.449	16714338	374063	50.336	%	V	RT:22.449
Total		33205775	1061446				

Supplementary Figure 84. HPLC spectra of rac-3t

<Chromatogram>

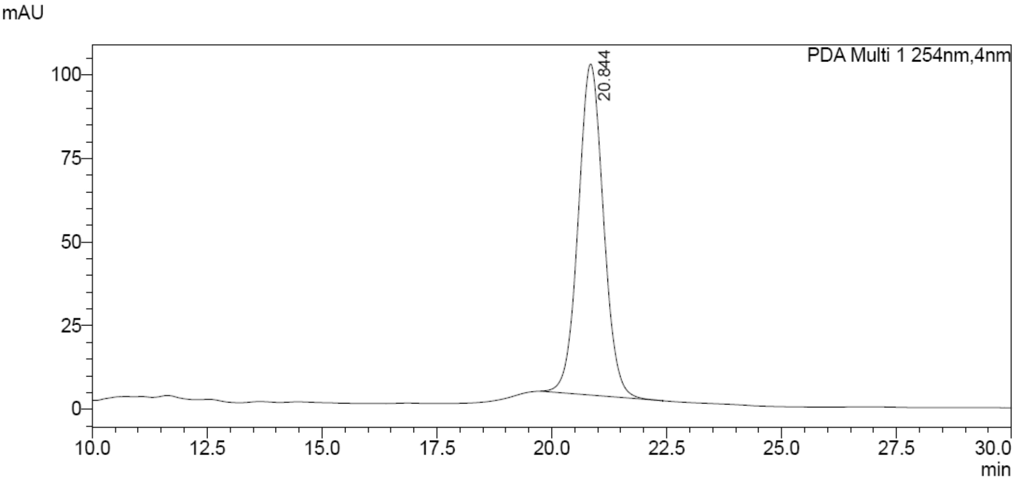

<Peak Table>

PDA Ch1 254nm

Peak#	Ret. Time	Area	Height	Conc.	Unit	Mark	Name
1	20.844	3774227	98689	100.000	%		RT:20.844
Total		3774227	98689				

Supplementary Figure 85. HPLC spectra of 3t

<Chromatogram>

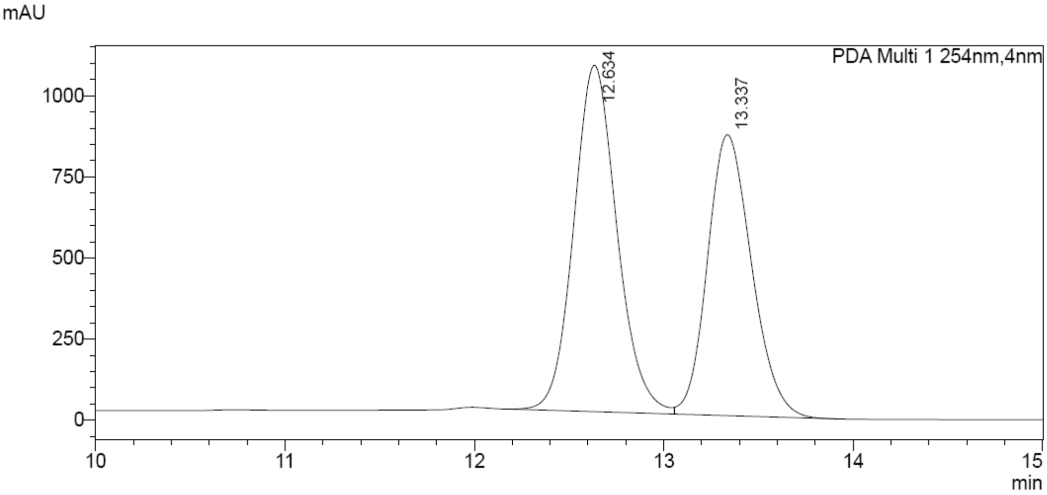

<Peak Table>

PDA Ch1 254nm

Peak#	Ret. Time	Area	Height	Conc.	Unit	Mark	Name
1	12.634	16957739	1063813	54.744	%		RT:12.634
2	13.337	14018414	863336	45.256	%	V	RT:13.336
Total		30976153	1927150				

Supplementary Figure 86. HPLC spectra of rac-3u

<Chromatogram>

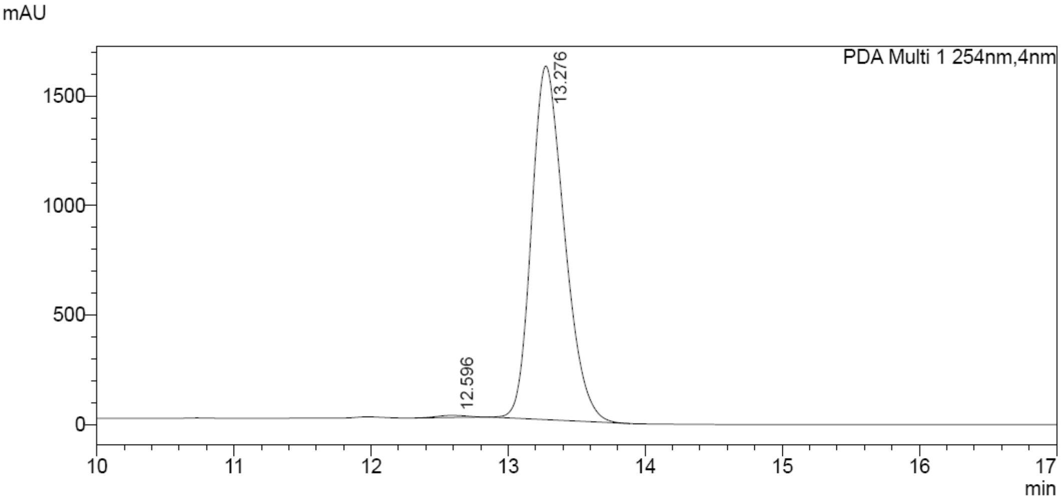

<Peak Table>

PDA Ch1 254nm

Peak#	Ret. Time	Area	Height	Conc.	Unit	Mark	Name
1	12.596	145511	10024	0.538	%		RT:12.596
2	13.276	26902181	1605664	99.462	%		RT:13.276
Total		27047692	1615688				

Supplementary Figure 87. HPLC spectra of 3u

Supplementary References

1. Kammel, R & Hyausek, J. Synthesis and rearrangement of substituted S-(1-benzofuran-2(3H)-one-3-yl) isothiouronium-bromides. *ECSOC*, **2013**, 1-30(2013).
